# Dynamic chromatin organization and regulatory interactions in human endothelial cell differentiation

**DOI:** 10.1016/j.stemcr.2022.11.003

**Published:** 2022-12-08

**Authors:** Kris G. Alavattam, Katie A. Mitzelfelt, Giancarlo Bonora, Paul A. Fields, Xiulan Yang, Han Sheng Chiu, Lil Pabon, Alessandro Bertero, Nathan J. Palpant, William S. Noble, Charles E. Murry

**Affiliations:** 1 Department of Laboratory Medicine and Pathology, University of Washington, 1959 NE Pacific Street, Seattle, WA 98195, USA; 2 Center for Cardiovascular Biology, University of Washington, 850 Republican Street, Brotman Building, Seattle, WA 98109, USA; 3 Institute for Stem Cell and Regenerative Medicine, University of Washington, 850 Republican Street, Seattle, WA 98109, USA; 4 Department of Genome Sciences, University of Washington, William H. Foege Hall, 3720 15th Avenue NE, Seattle, WA 98195, USA; 5 Institute for Molecular Bioscience, The University of Queensland, Brisbane, QLD 4072, Australia; 6 Centre for Cardiac and Vascular Biology, The University of Queensland, Brisbane, QLD 4072, Australia; 7 Sana Biotechnology, Seattle, WA 98102, USA; 8 School of Biomedical Sciences, The University of Queensland, Brisbane, QLD 4072, Australia; 9 Paul G. Allen School of Computer Science and Engineering, University of Washington, Seattle, WA 98195, USA; 10 Department of Medicine/Cardiology, University of Washington, 1959 NE Pacific Street, Seattle, WA 98195, USA; 11 Department of Bioengineering, University of Washington, 1959 NE Pacific Street, Seattle, WA 98195, USA

**Keywords:** endothelium, cardiomyocytes, differentiation, genome organization, chromatin, transcription

## Abstract

Vascular endothelial cells are a mesoderm-derived lineage with many essential functions, including angiogenesis and coagulation. The gene-regulatory mechanisms underpinning endothelial specialization are largely unknown, as are the roles of chromatin organization in regulating endothelial cell transcription. To investigate the relationships between chromatin organization and gene expression, we induced endothelial cell differentiation from human pluripotent stem cells and performed Hi-C and RNA-sequencing assays at specific time points. Long-range intrachromosomal contacts increase over the course of differentiation, accompanied by widespread heteroeuchromatic compartment transitions that are tightly associated with transcription. Dynamic topologically associating domain boundaries strengthen and converge on an endothelial cell state, and function to regulate gene expression. Chromatin pairwise point interactions (DNA loops) increase in frequency during differentiation and are linked to the expression of genes essential to vascular biology. Chromatin dynamics guide transcription in endothelial cell development and promote the divergence of endothelial cells from cardiomyocytes.

## Highlights

• In endothelial differentiation, compartment transitions regulate gene expression
• New TAD boundaries and chromatin loop anchors coordinate endothelial activation
• Frequent, growing chromatin loops accompany essential endothelial gene expression
• Cardiomyocytes evidence a markedly different topology versus endothelial cells

## Teaser

Alavattam, Mitzelfelt, and colleagues reveal that long-range *cis* contacts increase in endothelial cell differentiation, as do hetero-euchromatic compartment transitions, which are tightly associated with transcription. Specification occurs alongside new TAD boundaries and chromatin loop anchors, features that distinguish endothelial cells from cardiomyocytes, which evidence a dissimilar topology. Multiple forms of chromatin organization guide transcription in differentiation, driving endothelial divergence from cardiomyocytes.

## Introduction

Endothelial cells, a mesoderm-derived cell population, line the entirety of the circulatory system. Their functions are complex and critical, including angiogenesis, blood clotting, barrier function, vasomotor function, and fluid/nutrient filtration. Endothelial cell dysfunction is a prominent feature of many pathological conditions, including nearly all cardiovascular diseases (Rajendran et al., 2013). Complex transcriptional changes mediate both endothelial cell development and dysfunction (De Val and Black, 2009; Xu, 2014). It remains largely unknown what brings about such changes in gene expression.

The role of 3D chromatin organization in gene expression is an active area of study. However, to date, few studies have examined the genome organization of endothelial cells (Niskanen et al., 2018; Rao et al., 2014), and none have made use of a model for endothelial cell differentiation in which all samples are clonal, having been differentiated from the same source. To fill this gap, we performed a modified version of a previously developed protocol to induce endocardial-like endothelial differentiation from human pluripotent stem cells (hPSCs; Palpant et al., 2015, 2017) and, taking advantage of *in situ* DNase Hi-C (Ramani et al., 2016), a form of high-throughput chromosome conformation capture with sequencing, we investigated global chromatin organization at specific time points in endothelial cell development. In performing RNA sequencing (RNA-seq), we correlated and contextualized Hi-C data with endothelial cell gene expression. Our results show that dynamic changes in chromatin organization, including genomic compartmentalization and topologically associating domains, are associated with changes in transcription. DNA loops (pairwise point interactions) are associated with the expression of essential genes in euchromatic genomic compartments. Altogether, this study provides a comprehensive look at dynamic 3D chromatin organization during human endothelial cell development and uncovers important relationships between 3D chromatin organization and transcription.

## Results

### Long-range *cis* contacts increase during endothelial cell differentiation

**Figure 1:**
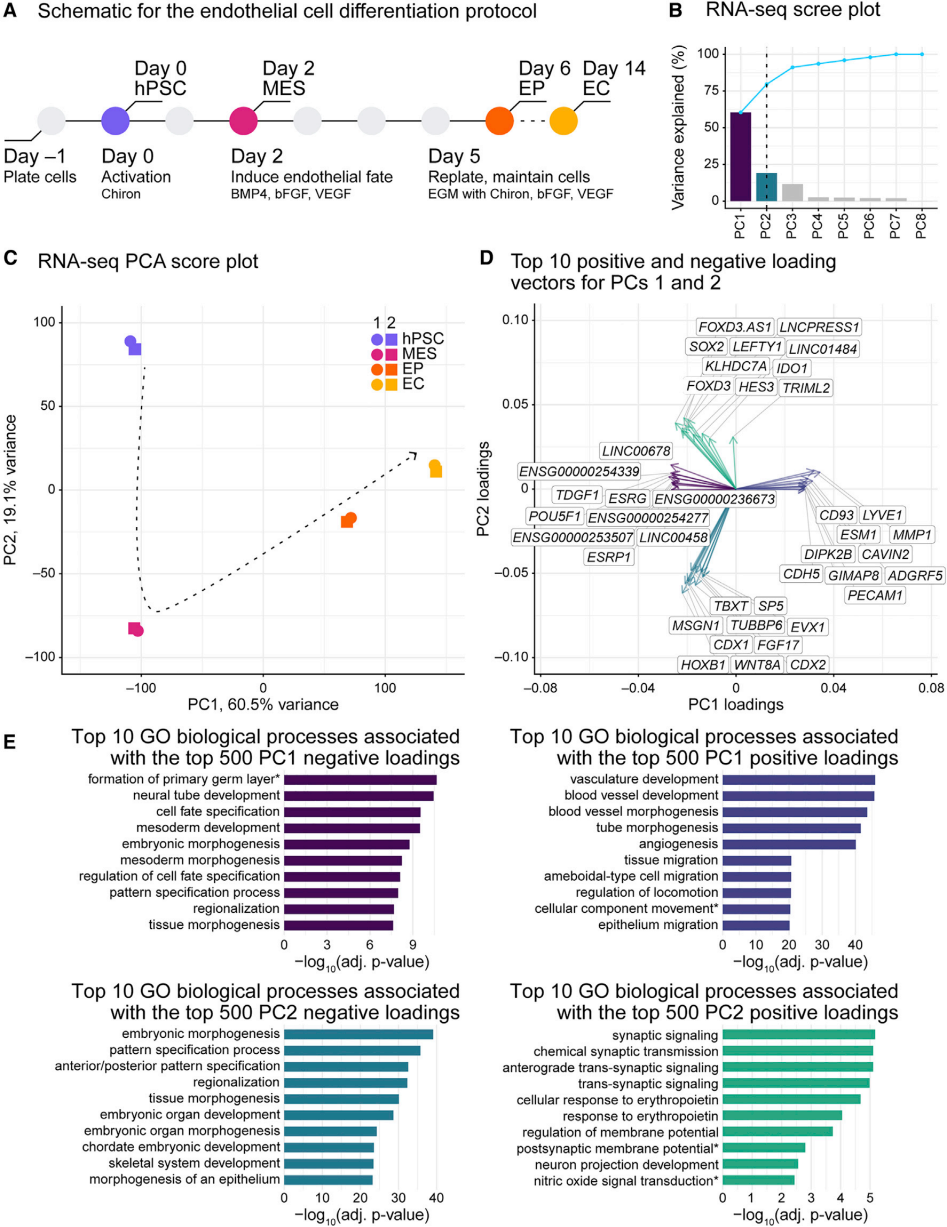
Transcription dynamics reveal epithelial-to-mesenchymal and mesenchymal-to-epithelial transitions in endothelial cell differentiation (A) Schematic of the endothelial cell differentiation protocol. hPSC, human pluripotent stem cells; MES, mesoderm cells; EP, endothelial progenitor cells; EC, endothelial cells; BMP4, bone morphogenetic protein 4; bFGF, basic fibroblast growth factor; VEGF, vascular endothelial growth factor; EGM, endothelial cell growth medium. (B) Bar chart showing proportions of variance explained for each principal component (PC) from principal component analysis (PCA) of RNA-seq data sampled from endothelial cell differentiation. Dashed black line, retained PCs—PCs 1 and 2—computed from Horn’s parallel analysis (Horn, 1965); solid blue line, cumulative proportion of explained variance. (C) PCA score plot for normalized RNA-seq data from endothelial cell differentiation with respect to PCs 1 and 2. Arrow, differentiation trajectory. (D) PCA loading plot showing the top 10 positive and negative loading vectors (genes) for each axis (PCs 1 and 2). (E) Bar charts depicting adjusted p values for the top 10 Gene Ontology (GO) biological process terms for the top 500 PC1 negative loading vectors (top left), the top 500 PC1 positive loading vectors (top right), the top 500 PC2 negative loading vectors (bottom left), and the top 500 PC2 positive loading vectors (bottom right). The p values are from hypergeometric tests with Bonferroni corrections. Terms with asterisks have been abbreviated: “formation of primary germ layer” is “cell fate commitment involved in formation of primary germ layer,” “cellular component movement” is “regulation of cellular component movement,” “postsynaptic membrane potential” is “regulation of postsynaptic membrane potential,” and “nitric oxide signal transduction” is “nitric oxide-mediated signal transduction.” See also Figure S1, Datasets S1 and S2, and Note S1.

To study the dynamics and functional significance of 3D chromatin organization in endothelial cell development, we modified and performed a stepwise protocol to induce endocardial-like endothelial differentiation from hPSCs (Palpant et al., 2015, 2017). Using the RUES2 embryonic stem cell line (Figure S1A), we recapitulated key signaling events in endothelial cell development. First, we induced the mesodermal lineage through activation of the WNT signaling pathway; then, we directed the cells to an endothelial fate through the addition of bone morphogenetic protein 4 (BMP4), basic fibroblast growth factor (bFGF), and vascular endothelial growth factor (VEGF; Figure 1A). We took samples across a differentiation time course: day 0, pluripotent cells (hPSCs); day 2, mesodermal cells (MESs); day 6, endothelial progenitor cells (EPs); and day 14, endothelial cells (ECs; Figure 1A). We obtained high-purity cell populations as determined by flow cytometry with antibodies raised against two EC markers, CD34 and CD31: EPs were >75% pure and ECs were >90% pure (Figures S1B and S1C).

To assess the quality of the time course samples, we prepared and analyzed bulk RNA-seq datasets from two independent differentiations for each time point (Dataset S1). To evaluate relationships among the RNA-seq datasets, we performed principal component analysis (PCA). Horn’s parallel analysis (Horn, 1965) revealed that, of the eight principal components (PCs), the first two are significant (Figure 1B); PC1 accounted for 60.5% of variance, and PC2 accounted for 19.1% (Figures 1B and 1C). A score plot for PCs 1 and 2 revealed the tight clustering of biological replicates and, in the separation of time points, an apparent EC developmental trajectory in which PC1 separated hPSCs and MESs from EPs and ECs, while PC2 separated mesenchymal cells (MESs) from epithelial cells (hPSCs, EPs, and ECs) (Figure 1C). Consistent with this, analysis of component loading vectors revealed the presence and enrichment of genes associated with endothelial development along the PC1 positive axis, formation of the embryonic primary germ layer on the PC1 negative axis, neural signaling and projection on the PC2 positive axis, and embryonic development and anterior/posterior pattern specification along the PC2 negative axis (Figures 1D and 1E and Dataset S2). Results from additional gene expression analyses were consistent with endothelial specification (Figures S1D–S1F, Dataset S2, and Note S1). Together with functional assays (Palpant et al., 2015, 2017) and the developmental trajectory uncovered by PCA, these results confirm cell identities and indicate that our differentiation protocol recapitulates the epithelial-to-mesenchymal and subsequent mesenchymal-to-epithelial transitions that occur in endothelial development.

To examine changes in chromatin organization over the course of EC differentiation, we prepared and analyzed *in situ* DNase Hi-C (Ramani et al., 2016) datasets from the same two independent differentiations for each time point. Various quality control checks revealed the data are of high quality (Figure S2, Dataset S1, and Note S2). Thus, for subsequent analyses of chromatin organization, we pooled replicates to increase the sequencing depth for each cell type.

**Figure 2:**
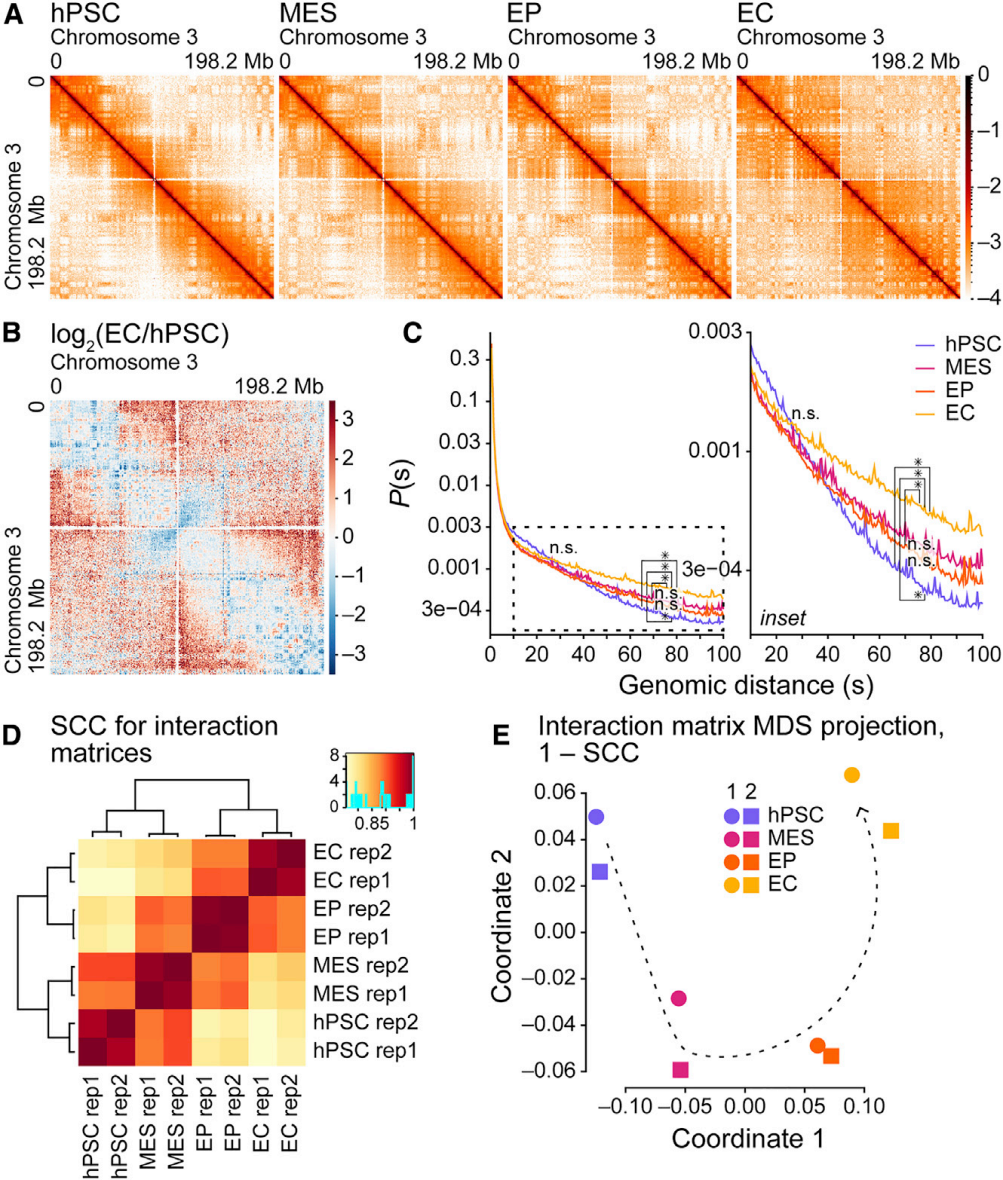
Long-range *cis* contacts increase during endothelial cell differentiation (A) Heatmaps of normalized Hi-C interaction frequencies (500-kb resolution, chromosome 3) in hPSCs, MESs, EPs, and ECs. (B) Log_2_ ratios of normalized Hi-C interaction frequencies (500-kb resolution, chromosome 3) for ECs and hPSCs. Red, interactions increased in ECs; blue, interactions increased in hPSCs. (C) Left: *cis* interaction frequency probabilities *P* stratified by distance *s* (Mb) over 0–100 Mb for Hi-C samples (500-kb resolution, autosomes). Inset, right: *P* stratified by *s* over 30–100 Mb. The p values at *s* of 30 and 75 Mb from pairwise t tests between samples (two independent replicates each) are shown; when adjusted with Benjamini-Hochberg *post hoc* tests, ^∗^p <0.05, n.s. (not significant). Adjusted p values at 30 Mb are n.s. (D) Hierarchically clustered heatmap of HiCRep (Yang et al., 2017) stratum-adjusted correlation coefficients (SCCs) for Hi-C sample replicates (500-kb resolution, autosomes). (E) Multidimensional scaling (MDS) projection of SCCs for Hi-C sample replicates (500-kb resolution); similarity measure: 1 − SCC. Arrow: differentiation trajectory. See also Figure S2, Dataset S1, and Note S2.

Next, we surveyed features of chromatin organization across EC differentiation. At all time points, chromosome-wide contact maps displayed patterns indicative of local and longer-range chromatin interactions (Figures 2A and 2B). These included an abundance of “near” *cis* interactions in hPSCs (e.g., strong interactions along the diagonal of the hPSC panel) that spread outward with differentiation, increasing the numbers of “far” *cis* interactions. Next, we examined the *cis* chromatin contact probability *P*(*s*) for pairs of genomic loci stratified by distance *s* (Lieberman-Aiden et al., 2009). Consistent with the *cis* contact maps, the proportion of long-range contacts increased over differentiation: hPSCs had the lowest proportion of long-range contacts >30 Mb; MESs and EPs had similar, higher, proportions of long-range contacts >30 Mb; and ECs exhibited the highest proportion of contacts >30 Mb (Figure 2C). These data reveal that long-range *cis* contacts increase over the course of EC differentiation.

To assess the influence of *cis* chromatin interactions as differentiation progress, we performed hierarchical clustering of stratum-adjusted correlation coefficients (SCCs) for *cis* interactions (Yang et al., 2017), clustering paired replicates while separating the datasets into two groups: an “early” group composed of hPSCs and MESs, and a “later” group composed of EPs and ECs (Figure 2D). Next, we performed multidimensional scaling (MDS) (Kruskal and Wish, 1977) of *cis* interaction maps using 1 − SCC as a measure of similarity. MDS paired replicates and arranged the samples in order of time point, revealing an apparent EC differentiation trajectory (Figure 2E) that resembles the epithelial-to-mesenchymal and mesenchymal-to-epithelial transitions captured by PCA of RNA-seq data (Figures 1B–1E). Together, these results indicate that changes in chromatin organization—including a gross increase in long-range *cis* chromatin contacts—are a key feature of EC differentiation, separating and ordering datasets by time point.

### Dynamic compartmentalization reflects endothelial cell-specific changes in gene expression

Given these findings, we sought to understand how chromatin organization changes across differentiation and the functional significance of such changes. We investigated three forms of chromatin organization: genomic compartments, topologically associating domains, and peaks of elevated contact frequency referred to as “pairwise point interactions.” Pairwise point interactions are thought to represent DNA “loops”; however, we avoid using the term “loop” for its multiple meanings and interpretations as described in a recent review of chromosome organization (Mirny et al., 2019).

**Figure 3:**
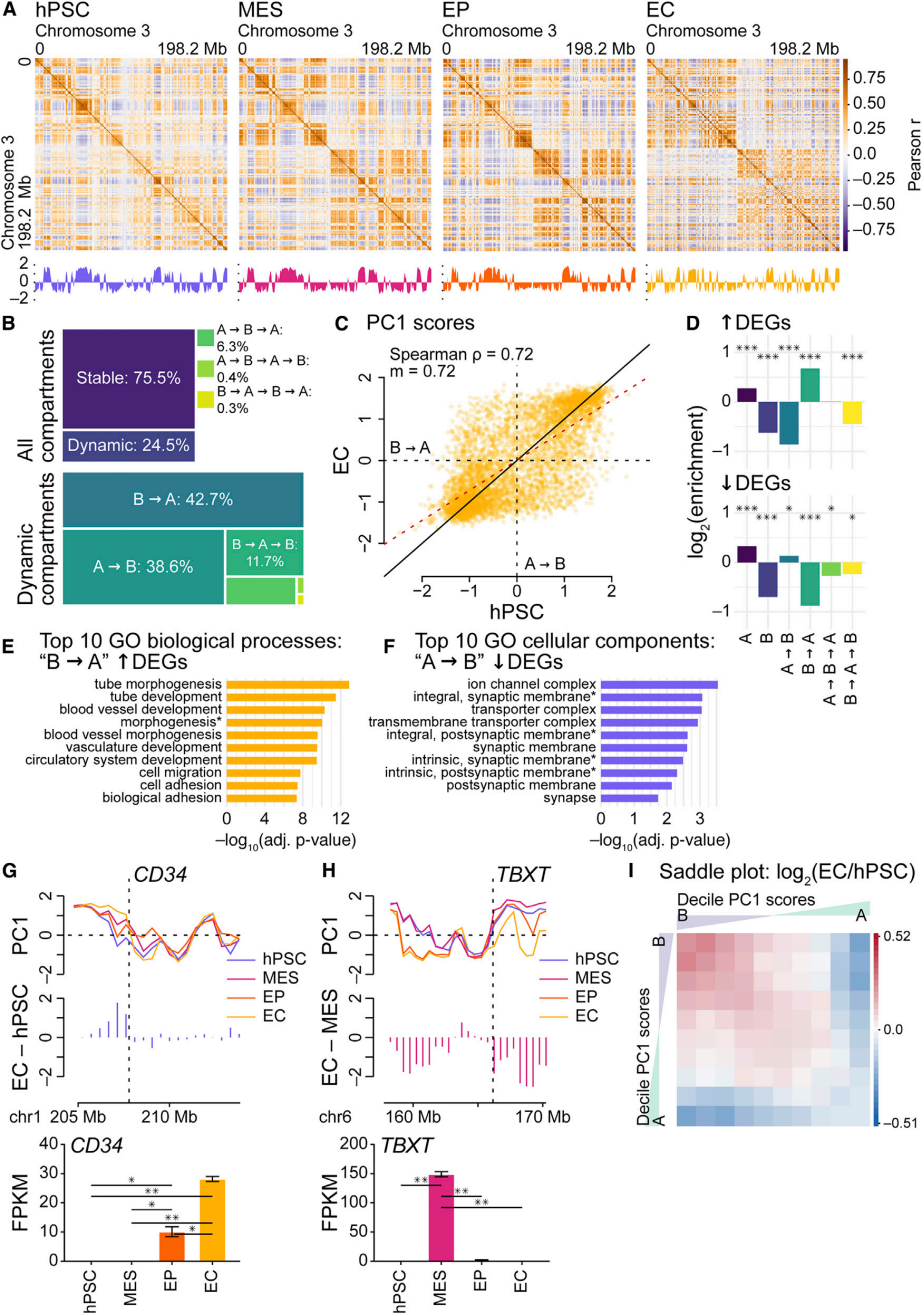
Dynamic compartmentalization reflects endothelial cell-specific changes in gene expression (A) Top: Pearson correlation coefficient matrices for normalized Hi-C interaction frequencies (500-kb resolution, chromosome 3) for endothelial cell samples. Bottom: principal component 1 (PC1) from principal component analysis. (B) Top: tree map showing proportions of stable and dynamic genomic compartments in differentiation. Bottom: tree map showing, for dynamic genomic compartments, the proportions of types of compartment switches. (C) Scatterplot comparing PC1 scores from hPSC and EC Hi-C samples (500-kb resolution, autosomes). ρ, Spearman correlation coefficient; m, regression slope; red dashed line, regression line; black solid line, x = y. (D) Bar charts depicting log_2_ enrichment of up- (top) and downregulated (bottom) differentially expressed genes (DEGs) with respect to stable and dynamic genomic compartments (500-kb resolution, autosomes). Log_2_ values, observed/gene density. DEGs were called via DESeq2 analysis (Love et al., 2014), EC versus hPSC (adjusted p < 0.05, absolute log_2_ fold change >1). Enrichment significantly different via chi-square tests with Yates corrections: ^∗^p < 0.05, ^∗∗∗^p < 0.001. (E and F) Bar charts depicting adjusted p values for the top 10 GO biological process or cellular component terms for genes located within bins (500-kb resolution, autosomes) that undergo (E) B-to-A and (F) A-to-B compartment transitions in endothelial specification. The p values are from hypergeometric tests with Bonferroni corrections. Terms with asterisks have been abbreviated: in (E), “morphogenesis” is “anatomical structure formation involved in morphogenesis,” and in (F), “integral, synaptic membrane” is “integral component of synaptic membrane,” “integral, postsynaptic membrane” is “integral component of postsynaptic membrane,” “intrinsic, synaptic membrane” is “intrinsic component of synaptic membrane,” and “intrinsic, postsynaptic membrane” is “intrinsic component of postsynaptic membrane.” (G and H) Top: line plots for PC1 scores at and within the vicinity of (G) *CD34* (chr1: 207.88–207.91 Mb) and (H) *TBXT* (chr6: 166.16–166.17 Mb) for Hi-C samples (500-kb resolution). Middle: bar charts indicating change in PC1 scores via the subtraction of (G) hPSC and (H) MES PC1 scores from EC PC1 scores. Bottom: bar charts for RNA-seq expression levels (FPKM) of (G) *CD34* and (H) *TBXT* in differentiation. The p values are from pairwise t tests between samples (two independent replicates each); when adjusted with Benjamini-Hochberg *post hoc* tests: ^∗^p < 0.05, ^∗∗^p < 0.01. Bar, mean; error bars, standard error of the mean. (I) Log_2_ ratio of “saddle plots,” 10 × 10 decile-binned matrices quantifying the strength of ranked PC1 scores, for Hi-C samples (500-kb resolution, autosomes); red, interactions higher in ECs; blue, interactions higher in hPSCs. See also Figure S3 and Dataset S2.

To begin, we focused on genomic compartments, the “plaid” patterns of chromatin interactions evident in Hi-C heatmaps (Lieberman-Aiden et al., 2009). Genomic compartments represent at least two alternating states of chromatin, A and B, and each state preferentially interacts with loci in the same state. A compartments are associated with higher gene expression in euchromatin, while B compartments are associated with gene silencing in heterochromatin. To segregate genomic bins (500-kb resolution) into A/B compartments, we computed the PC1s of Pearson correlation-transformed contact matrices. We identified genomic compartments in all samples (Figure 3A). To assess changes in compartmentalization as ECs differentiate, we analyzed the proportions of stable and dynamic compartments. We found that approximately 25% of compartments are dynamic, undergoing one or more compartment switches across time points (Figures 3B, 3C, and S3A). Hierarchical clustering of Spearman correlation coefficients for PC1 scores paired and ordered replicates (Figure S3B); likewise, MDS paired replicates while revealing a differentiation trajectory similar to the other trajectories (Figures S3C, 1C, and 2E). Substantial numbers of compartment transitions take place in EC specialization, making compartment dynamism another key feature of EC differentiation.

In a study of differentiating cardiomyocytes (Bertero et al., 2019)—which share their origin in cardiogenic mesoderm with ECs (Palpant et al., 2015, 2017)—we found that compartment switches coincide with transcriptional regulation. To understand the influence of genomic compartmentalization on the dynamic transcriptomes of EC differentiation (Figures 1D, 1E, and S1D–S1F and Dataset S2), we calculated the enrichment of differentially expressed genes (DEGs; EC versus hPSC) with respect to stable and dynamic compartments (Figure 3D). Gene Ontology (GO) analyses (Ashburner et al., 2000; Chen et al., 2009; Gene Ontology Consortium, 2021) show that regions undergoing B-to-A transitions are enriched for DEGs upregulated in ECs, and these are associated with numerous functions in endothelial specification (Figure 3E and Dataset S2). Compartment changes are also associated with gene repression; e.g., in regions undergoing A-to-B transitions, DEGs downregulated in ECs are associated with neuronal development and function, genes that are suppressed in differentiation (Figure 3F and Dataset S2). Consistent with the enrichment of EC genes in B-to-A regions, the EC marker *CD34* is subject to a transition before elevated expression in EPs and ECs (Figure 3G). Similarly, the mesodermal marker *TBXT* is associated with an A-to-B transition, coincident with downregulation from MES to EP (Figure 3H). Thus, as with differentiating cardiomyocytes (Bertero et al., 2019), dynamic genomic compartmentalization is an important regulator of endothelial transcription.

Given that, during specification, long-range *cis* interactions increase (Figures 2A–2C) and 25% of the genome undergoes compartment transitions (Figures 3B, 3C, and S3A), we examined large-scale changes in genomic compartment strength across EC differentiation. We generated “saddle plots,” which quantify the strength of compartment segregation, for hPSCs and ECs. We noted that stronger *cis* contacts occur between homotypic regions in comparison to heterotypic regions (Figures 3I and S3D); in differentiation, the strength of *cis* interactions between B compartments increased, while the strength between A compartments decreased. To evaluate the effects of strengthened B compartments, we compared gene expression distributions to background distributions. Observed expression distributions in stable and dynamic B compartment regions are significantly lower than background distributions (Figure S3E), indicating that—consistent with the differentiation of other tissues (Bertero et al., 2019; Bonev et al., 2017)—dynamic, strengthening B compartments repress transcription in endothelial specification.

### Increasingly strengthened topologically associating domains regulate endothelial cell-specific gene expression

Next, we shifted focus to a different form of chromatin organization: topologically associating domains (TADs). TADs comprise local “neighborhoods” of increased chromatin contact frequency and are often delimited by insulator sequences (Dixon et al., 2012; Nora et al., 2012). Because TADs are thought to regulate gene expression (Dixon et al., 2012, 2015; Nora et al., 2012), we evaluated TAD features across EC differentiation. Using the insulation score approach to call TAD boundaries (Crane et al., 2015), we identified TADs in all samples taken across EC differentiation (Figure S4A).

**Figure 4:**
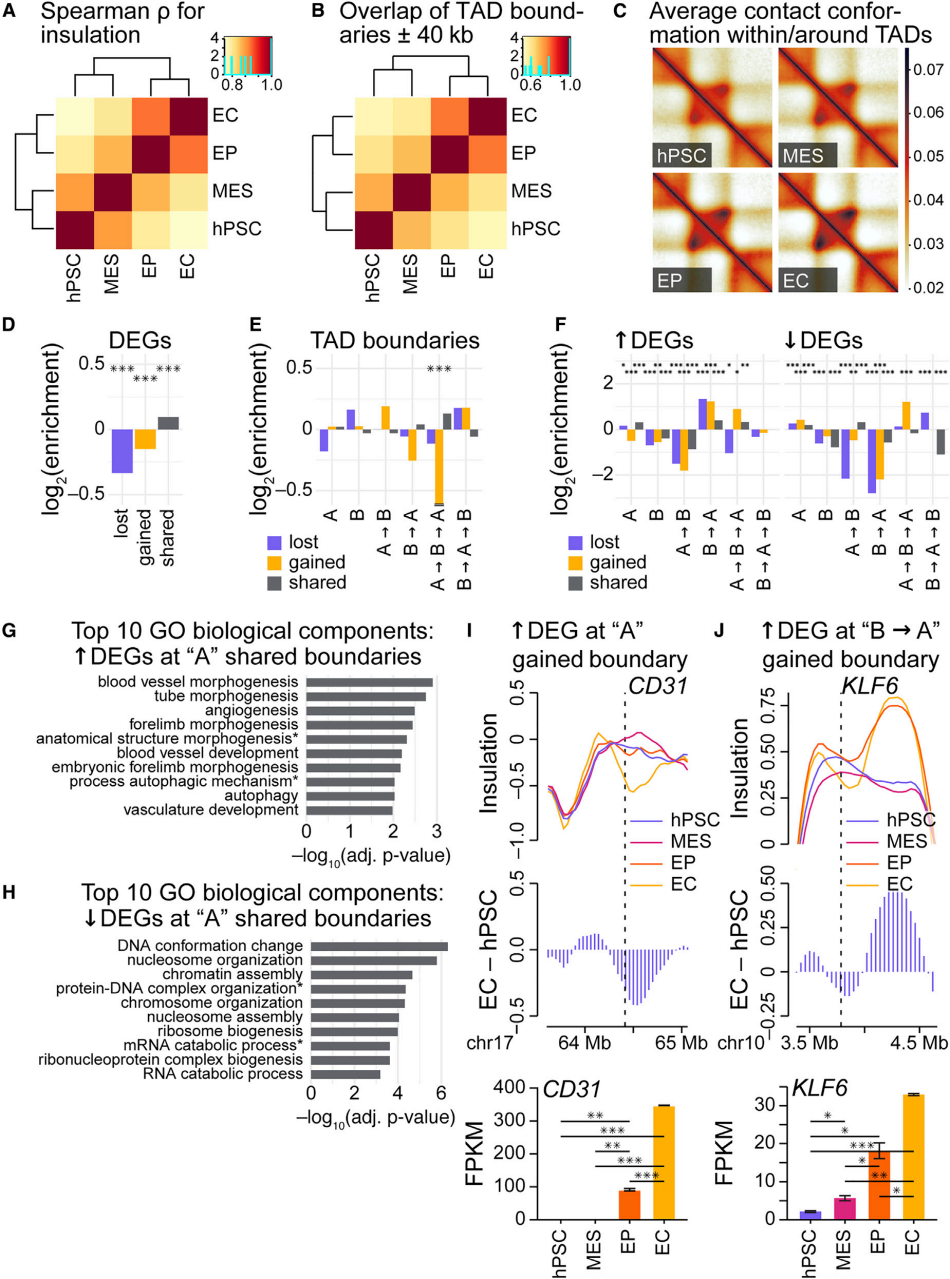
Increasingly strengthened topologically associating domains regulate endothelial cell-specific gene expression (A) Hierarchically clustered heatmap of Spearman correlation coefficients (ρ) for insulation scores from Hi-C samples (40-kb resolution, autosomes). (B) Hierarchically clustered heatmap for TAD-boundary set intersections for Hi-C samples using a window of ±40 kb around boundaries (40-kb resolution, autosomes). (C) Aggregate heatmaps representing average *cis* chromatin contact conformation around TADs for Hi-C samples (40-kb resolution, autosomes). (D) Bar charts depicting log_2_ enrichment of differentially expressed genes (DEGs) proximal to (±80 kb) TAD boundaries (40-kb resolution, autosomes) lost in differentiation (hPSC-specific), gained in differentiation (EC-specific), and shared between time points (hPSC, EC). Log_2_ values: observed/gene density. DEGs were called via DESeq2 analysis (Love et al., 2014), EC versus hPSC (adjusted p < 0.05, absolute log_2_ fold change >1). Enrichment significantly different via chi-square tests with Yates corrections: ^∗∗∗^p < 0.001. (E) Bar charts depicting log_2_ enrichment of lost, gained, and shared TAD boundaries in stable and dynamic compartments (500-kb resolution, autosomes). Log_2_ values: observed/TAD union set. Enrichment significantly different via chi-square tests with Yates corrections: ^∗^p < 0.05, ^∗∗^p < 0.01, ^∗∗∗^p < 0.001. Value for gained boundaries in A-to-B-to-A compartments: −1.2. (F) Bar charts depicting log_2_ enrichment of up- (left) and downregulated (right) DEGs proximal to (±80 kb) boundaries grouped by stable and dynamic compartments. Log_2_ values: observed/gene density. Enrichment significantly different via chi-square tests with Yates corrections: ^∗^p < 0.05, ^∗∗^p < 0.01, ^∗∗∗^p < 0.001. (G and H) Bar charts depicting adjusted p values for the top 10 Gene Ontology (GO) biological process terms for up- (G) and downregulated DEGs (H) proximal to (±80 kb) shared TAD boundaries in stable A compartments. The p values are from hypergeometric tests with Bonferroni corrections. Terms with asterisks have been abbreviated: in (G), “anatomical structure morphogenesis” is “anatomical structure formation involved in morphogenesis” and “process autophagic mechanism” is “process utilizing autophagic mechanism,” and in (H), “protein-DNA complex organization” is “protein-DNA complex subunit organization” and “mRNA catabolic process” is “nuclear-transcribed mRNA catabolic process.” (I and J) Top: line plots for insulation scores at and within the vicinity of (I) *CD31* (chr17: 64.32–64.41 Mb) and (J) *KLF6* (chr10: 3.776–3.785) for Hi-C samples (40-kb resolution). Middle: bar charts indicating changes in insulation score via the subtraction of hPSC insulation scores from EC insulation scores. Bottom: bar charts for RNA-seq expression levels (FPKM) of (I) *CD31* and (J) *KLF6* in differentiation. The p values are from pairwise t tests between samples (two independent replicates each); when adjusted with Benjamini-Hochberg *post hoc* tests: ^∗^p < 0.05, ^∗∗^p < 0.01, ^∗∗∗^p < 0.001. Bar, mean; error bars, standard error of the mean. See also Figure S4 and Datasets S1 and S2.

Hierarchical clustering of Spearman correlations for TAD insulation scores gave results similar to those of hierarchical clustering of separate forms of chromatin organization (Figures 4A, 2D, S2A, and S3B). Replicates were paired, and the time-course datasets were separated into groups of early—hPSC, MES—and later samples—EP, EC—indicating an overall change in insulation scores across differentiation. We observed a similar dynamism for TAD boundaries: when measuring boundary intersections with a window of ±40 kb, 45.2% of TAD boundaries change position in differentiation (Figure 4B and Dataset S1); when the window size is increased to ±80 kb, 20.9% of TAD boundaries change (Figure S4B and Dataset S1). Clustering of intersections (windows of ±40 kb) revealed the same branching of early and later cell types (Figure 4B). Of note, EP and EC share the highest proportion of TAD boundaries (windows of ±40 kb): EP shares 78.1% of its boundaries with EC, EC shares 78.0% of its boundaries with EP. Next, we examined insulation-score changes at boundaries. Although scatterplots revealed limited changes in insulation scores at boundaries (Figure S4C), the differences in insulation to the immediate left and right of boundaries—i.e., “TAD boundary strengths” (Crane et al., 2015)—increased in differentiation (Figure S4D). Analyses of the average chromatin contact conformation within and around TADs reveal that, as cells differentiate, *cis* chromatin interactions are increasingly restricted within TAD boundaries (Figure 4C); this constraint is stronger in EC versus hPSC (Figure S4E). Although nearly 55% of all TAD boundaries are conserved in differentiation, dynamic boundaries converge on an EC state, and this is accompanied by a steady increase in the numbers of intra-TAD chromatin contacts.

We hypothesized that nascent boundaries and increased intra-TAD contacts regulate gene expression changes necessary for differentiation. Thus, we analyzed the enrichment of DEGs (EC versus hPSC) proximal to (within ±80 kb of) boundaries lost in differentiation (hPSC-specific boundaries), boundaries gained in differentiation (EC-specific), and shared boundaries (common to hPSC and EC). We found that DEGs are depleted from lost and gained boundaries and enriched at shared boundaries (Figure 4D). Relative to shared boundaries, gene expression is decreased at lost and gained boundaries (Figure S4F). These data raise the possibility that unshared boundaries are, on average, associated with repressive chromatin environments.

To investigate this, we examined the interplay of TADs and compartments. TADs in A compartments are smaller than those in B compartments (Figure S4G), a likely consequence of gene regulation in gene-dense, transcriptionally active regions. TAD boundaries lost in specification are slightly depleted from stable A compartments and dynamic regions of the genome that transition to A compartments, and slightly enriched in stable and dynamic B compartments (Figure 4E, purple). Shared boundaries are slightly enriched at dynamic A regions (Figure 4E, gray). TAD boundaries gained in specification are slightly enriched at dynamic B regions and depleted from dynamic A regions (Figure 4E, yellow). Consistent with this, the proportions of gained boundaries across compartment categories differ significantly from the proportions of shared boundaries (Figure S4H). These data support the assertion that TAD boundaries gained in differentiation tend to be associated with repressive chromatin.

This led us to explore how TAD-compartment interrelationships influence gene expression. We examined the enrichment of up- and downregulated DEGs at TAD boundaries grouped by stable and dynamic compartments (Figure 4F). Regardless of boundary type, upregulated DEGs are depleted from boundaries in A-to-B regions and enriched at boundaries in B-to-A regions (Figure 4F, left); downregulated DEGs are depleted from both A-to-B and B-to-A regions (Figure 4F, right). Similarly, gene expression is generally higher at boundaries in B-to-A regions and lower at boundaries in A-to-B regions (Figure S4I). These results indicate that, although TAD boundaries gained in differentiation tend to be associated with repressive chromatin environments, those boundaries that form in regions undergoing repressive-to-active chromatin transitions are enriched in upregulated DEGs. Thus, the relationship between TADs and gene expression is contextual, influenced by chromatin environment.

Our analyses also revealed that shared TAD boundaries in stable A regions of the genome are slightly but significantly enriched in both up- and downregulated DEGs (Figure 4F, gray). GO analyses of upregulated DEGs output terms associated with EC-specific functions (Figure 4G and Dataset S2); DEGs downregulated in ECs (and thus upregulated in hPSCs) are associated with chromatin organization and lability (Figure 4H and Dataset S2). Analyses of shared boundaries in stable A compartments indicate that, beyond gross dynamic compartment switching, additional gene-regulatory mechanisms are at play in endothelial specification.

Upregulated DEGs with essential roles in EC biology were observed at a subset of gained boundaries as well: the expression of *CD31* (Figure S1C) increases alongside boundary formation in a stable A compartment (Figure 4I), and the expression of *KLF6*, which encodes a Kruppel-like transcription factor that regulates genes involved in angiogenesis, vascular repair, and remodeling (Gallardo-Vara et al., 2016), increases with boundary formation in a B-to-A region (Figure 4J).

### Long-range pairwise point interactions increase over differentiation and are associated with both gene activation and gene repression

**Figure 5:**
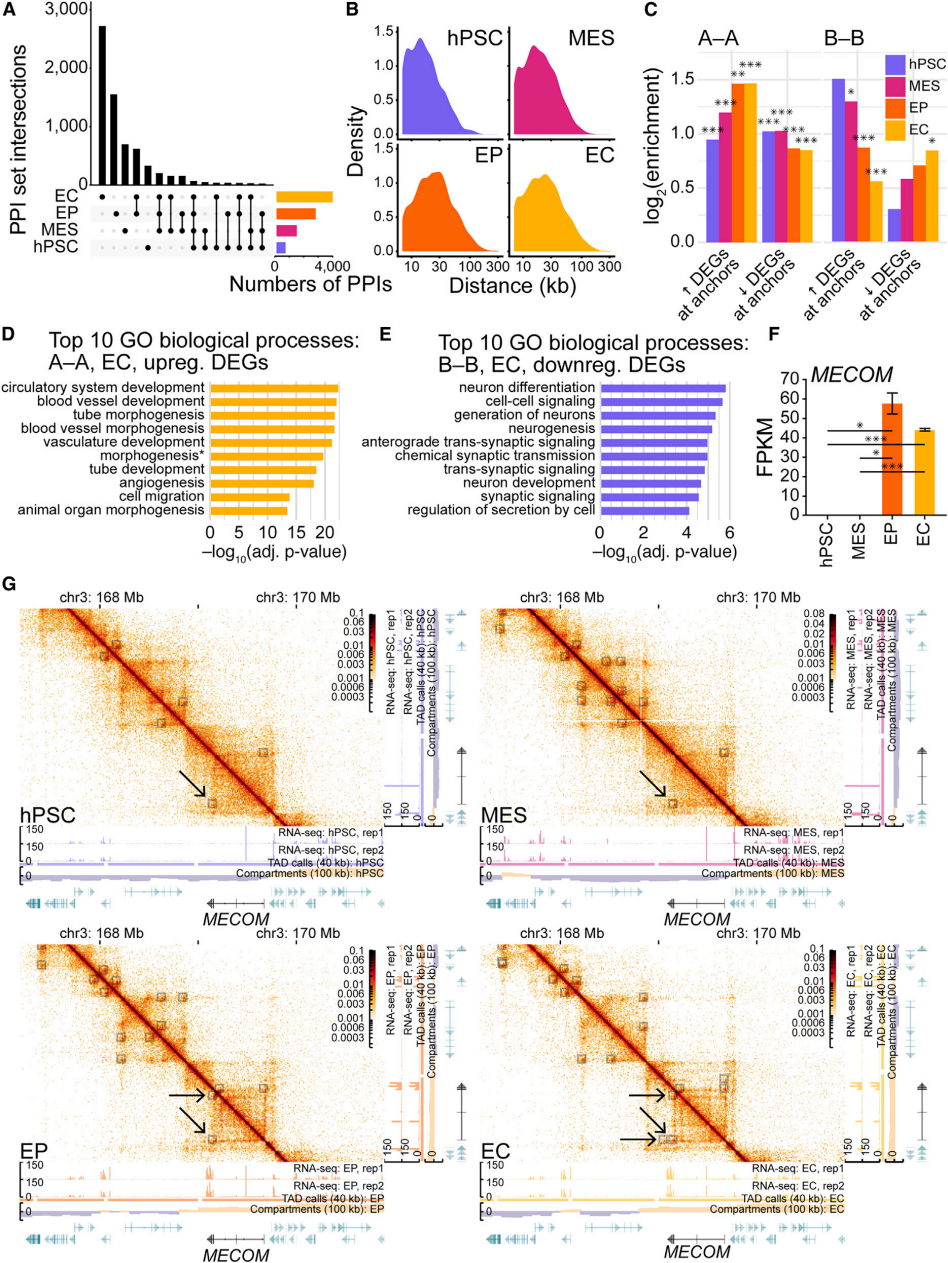
Long-range pairwise point interactions increase over differentiation and are associated with both gene activation and gene repression (A) UpSet plot showing intersections of HiCCUPS (Rao et al., 2014) pairwise point interaction (PPI) anchors from Hi-C samples (10-kb resolution, autosomes). Vertical bars, PPI-anchor intersection sizes; horizontal bars, sample set sizes; black circles, anchors present; linked black circles, anchors shared between samples; gray circles, anchors absent. (B) Density plots showing distributions of distances between PPI anchors from Hi-C samples (10-kb resolution, autosomes). (C) Bar charts showing log_2_ enrichment of up- and downregulated differentially expressed genes (DEGs) at PPI anchors stratified by compartment type (A-A, A-B, and B-B; 100-kb resolution, autosomes) for Hi-C samples (10-kb resolution, autosomes). DEGs were called via DESeq2 analysis (Love et al., 2014), EC versus hPSC (adjusted p < 0.05, absolute log_2_ fold change >1). Enrichment significantly different via chi-square tests with Yates corrections: ^∗^p < 0.05, ^∗∗^p < 0.01, ^∗∗∗^p < 0.001. (D and E) Bar charts depicting adjusted p values for the top 10 Gene Ontology (GO) biological process terms for DEGs associated with (D) A-A and (E) B-B PPIs. The p values are from hypergeometric tests with Bonferroni corrections. Terms with asterisks have been abbreviated: in (D), “morphogenesis” is “anatomical structure formation involved in morphogenesis.” (F) Bar chart for *MECOM* RNA-seq expression levels (FPKM) in differentiation. The p values are from pairwise t tests between samples (two independent replicates each) adjusted with Benjamini-Hochberg *post hoc* tests: ^∗^p < 0.05, ^∗∗∗^p < 0.001. Bar, mean; error bars, standard error of the mean. (G) Visualization of PPIs associated with *MECOM* (chr3: 169.08–169.66 Mb). Heatmaps and tracks for normalized Hi-C interaction frequencies (10-kb resolution), RNA-seq signal (unadjusted), TADs (40-kb resolution), genomic compartments (100-kb resolution; gold, A compartment; purple, B compartment), and genes (green and black) are shown. Gray transparent squares over heatmaps, PPIs; arrows, PPIs associated with *MECOM*. See also Figure S5, Datasets S1 and S2, and Note S3.

Increasing evidence supports the importance of DNA pairwise point interactions (PPIs) in transcriptional regulation and suggests the existence of PPIs that function in specific aspects of development (Bonev et al., 2017; Gorkin et al., 2014). These interactions are thought to arise from the clustering of regulatory elements and genes through chromatin looping mechanisms (Rao et al., 2014). Using the point interaction-calling package HiCCUPs (Rao et al., 2014), we investigated PPI formation in EC differentiation. We identified increasing numbers of PPIs in differentiation: 623 in hPSCs to 3,881 in ECs (Figure 5A). Most loops are specific to one time point; few loops are shared by more than two time points. We observed the greatest number of stage-specific loops in ECs, suggesting a role for PPI-mediated transcriptional regulation in maturation. We examined the distances between PPI anchors across differentiation and observed a progressive expansion of PPI sizes (Figure 5B). Thus, endothelial specification sees PPIs increase in both frequency and distance.

Since PPI dynamics occur amid changes in genomic compartmentalization (Figures 3 and S3), and since TAD-compartment interplay is associated with essential gene expression (Figures 4 and S4), we investigated PPI-compartment interrelationships. From MES to EC, the proportion of PPIs with both anchors in A compartments (A-A) increases, while the proportion with anchors in B compartments (B-B) decreases (Figures S5A and S5B). To investigate whether nascent A-A PPIs are involved in the transcriptional activation of genes essential to EC development, we evaluated the genome-wide enrichment of DEGs (EC versus hPSC) at time-point-specific anchors in A/B compartments. We observed an enrichment of DEGs at PPIs in all time points (Figure 5C and Dataset S1). The enrichment of PPIs at upregulated DEGs is elevated in comparison to downregulated DEGs. PPIs anchored in A compartments are enriched for upregulated DEGs and depleted for downregulated DEGs; the enriched upregulated DEGs are associated with numerous functions in endothelial specification (Figure 5D and Dataset S2). This trend was reversed for PPIs anchored in B compartments; the genes associated with B-compartment PPIs have numerous functions in neurogenesis (Figure 5E and Dataset S2), raising the possibility that B-compartment PPIs function in a transcriptional repression mechanism that inhibits neuronal specification. In a reciprocal analysis, we noted even stronger trends for the enrichment of PPI anchors at DEGs (Figure S5C), indicating that the overlap between DEGs and PPI anchors does not occur by chance. Our data suggest that PPIs facilitate both transcriptional activation within the A compartment and transcriptional repression within the B compartment.

Several genes with well-established roles in EC development associate with PPIs as their transcription increases. A prominent example is *MECOM*, which encodes a transcription factor that promotes arterial EC identity (McCracken et al., 2022). *MECOM* is expressed in EPs and ECs (Figure 5F), and comes to overlap multiple PPI anchors in differentiation (Figure 5G). Additional examples include *VEGFC* (Figure S5D), *KDR* (Figure S5E), and *TFPI* (Figure S5F and Note S3). Numerous genes repressed in EC differentiation are associated with B-compartment PPIs, including genes associated with tissue patterning and neuronal development; these include *EPHB6* (Figure S5G), *GABRB3/A5* (Figure S5H), and *PTPRZ1* (Figure S5I). We also observed examples of B-compartment PPI anchors associating with genes that code for factors with antiangiogenic properties, including *FOXC1* (Figure S5J) and *ISM1* (Figure S5K and Note S3).

### Chromatin organization reveals the developmental divergence of endothelial cells and cardiomyocytes

**Figure 6:**
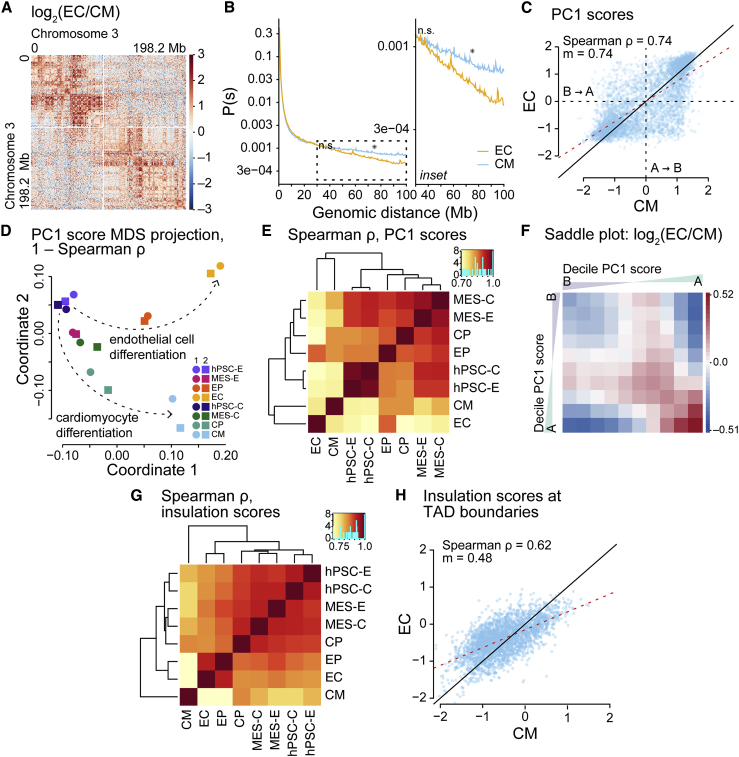
Chromatin organization reveals the developmental divergence of endothelial cells and cardiomyocytes (A) Log_2_ ratios of normalized Hi-C interaction frequencies (500-kb resolution, chromosome 3) for ECs versus cardiomyocytes (CMs). Red, interactions increased in ECs; blue, interactions increased in CMs. (B) Left: *cis* interaction frequency probabilities p stratified by distance *s* (Mb) over 0–100 Mb for Hi-C samples (500-kb resolution, autosomes). Inset, right: p stratified by *s* over 30–100 Mb. The p-values at *s* of 30 and 75 Mb are from pairwise t tests between samples (two independent replicates each); when adjusted with Benjamini-Hochberg *post hoc* tests: ^∗^p < 0.05, n.s. (not significant). (C) Scatterplot comparing PC1 scores from Hi-C samples (500-kb resolution, autosomes). ρ, Spearman correlation coefficient; m, regression slope; red dashed line, regression line; black solid line, x = y. (D) MDS projection of PC1 scores for Hi-C samples (500-kb resolution, autosomes) taken from EC and CM differentiation. Similarity measure: 1 − ρ. hPSC-E, human pluripotent stem cells from endothelial cell differentiation; MES-E, mesoderm cells from endothelial cell differentiation; hPSC-C, human pluripotent stem cells from cardiomyocyte differentiation; MES-C, mesoderm cells from cardiomyocyte differentiation; CP, cardiomyocyte progenitor cells. (E) Hierarchically clustered heatmap of Spearman correlation coefficients (ρ) for PC1 scores from Hi-C samples (500-kb resolution, autosomes). (F) Log_2_ ratios of saddle plots for Hi-C samples (500-kb resolution, autosomes); red, interactions higher in EC; blue, interactions higher in CM. (G) Hierarchically clustered heatmap of Spearman correlation coefficients for insulation scores from Hi-C samples (40-kb resolution, autosomes). (H) Scatterplot comparing insulation scores from Hi-C samples (40-kb resolution, autosomes). ρ, Spearman correlation coefficients; m, regression slope; black dashed line, regression line; black solid line, x = y. See also Figure S6 and Dataset S1.

Given their developmental origin from cardiogenic mesoderm (Palpant et al., 2015, 2017), we sought to compare genome organization in ECs versus cardiomyocytes, analyzing published time-course cardiomyocyte datasets (Bertero et al., 2019) with respect to our EC datasets. We took the log_2_ ratio of EC and cardiomyocyte (CM) contacts for a single chromosome, chromosome 3 (Figure 6A), noting elevated near-range interactions in ECs (red) and, in CMs, an increase in long-range interactions (blue). Beyond 30 Mb, there is a higher probability for *cis* contacts in CM versus EC (Figure 6B), indicating that, although long-range *cis* contacts increase as hPSCs differentiate to become ECs (Figures 2A–2C), longer-range *cis* contacts are present in—and a prominent feature of—genome organization in CM.

To examine the influence of *cis* chromatin interactions as ECs and CMs differentiate from their common origin, we performed hierarchical clustering of SCC scores, which apportioned samples into distinct groups related to time point and cell type (Figure S6A and Dataset S1). hPSC and MES cell types clustered into separate groups, and CM progenitor cells (CPs) were observed amid the MES samples. Increasingly differentiated cell types—EP, EC, and CM—occupied their own groups. Thus, *cis*-contact dynamism tightly correlates with the developmental divergence of the two cell types.

Given that, we investigated the influence of genomic compartments on EC and CM differentiation. Scatterplots for PC1 scores in EC versus CM revealed marked changes in compartment status in EC versus CM (Figures 6C and S6B). MDS of PC1 scores grouped replicates and outlined divergent differentiation trajectories (Figure 6D). Hierarchical clustering of Spearman correlation coefficients for PC1 scores paired replicates (Figure 6E) and revealed that the most differentiated samples, EC and CM, have the most distinct compartment profiles. We calculated and plotted the log_2_ ratio of EC and CM saddle plots, finding that *cis* interactions in B compartments are generally weaker in EC versus CM, while *cis* interactions between A compartments are generally stronger (Figures 6F and S6C). Taken together, these data reveal genomic compartmentalization as an important distinguishing feature of the two lineages. Considering their varying transcriptomes (Bertero et al., 2019), dynamic compartmentalization likely influences the two transcription programs.

Next, we asked whether TAD dynamics varied between EC and CM. Hierarchical clustering of TAD insulation scores grouped early cell states—hPSC, MES, and also CP—and separated out later cell states—EP, EC, and CM (Figure 6G). Of note, insulation scores in CMs were markedly different from all other cell types. Hierarchical clustering of TAD boundary set intersections (windows of ±40 and ±80 kb) apportioned samples similar to clustered insulation scores (Figure S6D and Dataset S1), separating early states from later states. We homed in on TAD dynamics across EC and CM by drawing scatterplots for insulation scores at boundaries, revealing strong changes in insulation scores in EC versus CM (Figures 6H and S6E) and a trend in which CM insulation scores tend to be higher when EC insulation scores are lower. These results suggest that the two lineages develop distinct chromatin topologies: one in which intra-TAD chromatin interactions are tightly bounded in ECs, serving to hinder longer-range chromatin interactions; on the other hand, CM TAD boundaries are less restrictive, and thus CM interactions are able to form longer *cis* chromatin interactions.

## Discussion

In this study, we advanced a model system in which hPSCs are differentiated to ECs, and we used this system to present a comprehensive look at chromatin organization on its own and with respect to the dynamic transcriptomes of differentiation (Note S4).

Our examination of genomic compartmentalization in EC differentiation revealed that ∼25% of the genome is dynamic, transitioning from transcriptionally active, euchromatic A compartments to repressive, heterochromatic B compartments or vice versa. Regions that transition from B to A are enriched in upregulated DEGs related to endothelial specification; regions that transition from A to B are enriched in downregulated DEGs related to neurogenesis. We also observed the increasing strength of B compartments in differentiation, suggesting the compaction of chromatin (Bertero et al., 2019). Given the large changes in transcription that occur in EC differentiation, it is possible that dynamic, strengthening B compartments function to repress transcription through chromatin inaccessibility mechanisms. These findings are in line with those reported in recent Hi-C analyses of CMs and other models for differentiation (Bertero et al., 2019; Paulsen et al., 2019) and, taken together, indicate that dynamic genomic compartmentalization is an important, conserved regulator of cell-type-specific transcription.

In addition to genomic compartmentalization, we assessed TADs and PPIs, two other forms of chromatin organization. TADs and PPIs are similar in that both arise through a process of chromatin loop extrusion (Fudenberg et al., 2016; Nora et al., 2017; Sanborn et al., 2015). In loop extrusion, the multisubunit protein complex cohesin entraps small loops of chromatin inside its lumen; through progressive extrusion, the loops are enlarged, ceasing to grow when cohesin colocalizes with the transcription factor/insulator protein CTCF, the CTCF cofactor MAZ (Ortabozkoyun et al., 2022), and likely other “architectural proteins” (Rowley et al., 2016). It has been suggested that cohesin is subject to rapid turnover (Haarhuis et al., 2017; Nora et al., 2017), limiting the time span for cohesin-chromatin interactions and, thus, loop extrusion. Cohesins can also extend beyond CTCF-bound consensus sequences: under conditions where cohesin turnover is prolonged or stopped, cohesin appears to move beyond CTCF (Schwarzer et al., 2017; Tedeschi et al., 2013). We observed that most PPIs are unique to each stage of EC differentiation, with each successive stage in our time-point analysis seeing more and more PPIs. The EC stage exhibits the most PPIs in addition to the strongest TAD boundaries and highest number of intra-TAD contacts. Considering these findings together, we speculate that cohesin loading is increased and turnover is prolonged, allowing for strengthened TAD boundaries and increased numbers of PPIs in maturation. These findings are consistent with observations in a prior study (Niskanen et al., 2018), which reported high levels of TAD connectivity, identifying EC-specific long-range interactions between TADs enriched for histone H3 trimethylated at lysine 9 (H3K9me3), a marker of constitutive heterochromatin. While it remains to understand how the strengthened TADs and numerous PPIs arise in ECs, our findings indicate that these features are influenced by stable and dynamic compartments to regulate transcription necessary for differentiation (Note S4).

We also performed comparative analyses of dynamic chromatin organization in endothelial specification versus CM specification, observing that, although B compartments strengthen in EC differentiation, B compartments do not strengthen to the extent seen in CM differentiation (Bertero et al., 2019). Alongside this, insulation scores are generally lower at TAD boundaries in EC versus CM, indicating that *cis* chromatin interactions are less restrained in EC versus CM. These observations indicate that high levels of heterochromatinization and long-range chromatin contacts are prevalent features of genome organization in CM versus EC. The CM nuclear environment is notable for a *trans*-interaction network of *TTN*-associated genes facilitated by the muscle-specific splicing protein RBM20 (Bertero et al., 2019). Could it be that CM differentiation, with its preponderance of long-range interactions, sees the establishment of a nuclear environment that facilitates functionally significant regulatory *trans* interactions, while EC development sees the development of a nuclear environment that bounds long-range contacts and comes to support regulation through predominantly *cis* forms of chromatin organization, e.g., PPIs?

Attempting to address this and other questions will undoubtedly fuel further research. With this study, we provide a comprehensive analysis of 3D chromatin organization in a model of EC development that will be a key resource for studying EC biology in health and disease.

## Experimental procedures

Details are provided in the supplemental experimental procedures.

### Resource availability

#### Corresponding author

Further information and requests should be directed to and will be fulfilled by co-lead contact Charles E. Murry (murry@uw.edu).

#### Materials availability

This study did not generate new unique reagents.

#### Data and code availability

Data have been deposited in the 4DN data portal and are publicly available at https://data.4dnucleome.org/Alavattam-Mitzelfelt-endothelial-differentiation-chromatin; previously published CM differentiation data are available from GEO: GSE106690 (Bertero et al., 2019). Source code for analyses performed in this study is available at github.com/Noble-Lab/2020_kga0_endothelial-diff.

### Statistics

No statistical methods were used to predetermine sample sizes. No data were excluded from analyses. The experiments were not randomized and investigators were not blinded to allocation during experiments and assessment. Where appropriate, the mean is reported as a measurement of central tendency, and the SEM is used as a measure of precision. Statistical significance was thresholded at α = 0.05; p < α are considered significant. Sample and replicate numbers are reported in figure and supplemental figure captions where appropriate. Statistical analyses were performed using base R (version 4.1) or various software packages (supplemental experimental procedures). Strategies for stratification, sampling, and enrichment are described in the supplemental experimental procedures, as are statistical tests used in this study.

## Author contributions

K.G.A., K.A.M., W.S.N., and C.E.M. wrote the manuscript with edits provided by all other authors. K.G.A., K.A.M., G.B., W.S.N., and C.E.M. designed the experiments. K.A.M. conducted wet-lab experiments; K.G.A., G.B., P.A.F., and X.Y. conducted dry-lab experiments. K.G.A., K.A.M., X.Y., L.P., A.B., N.J.P., H.S.C., W.S.N., and C.E.M. interpreted the results. W.S.N. and C.E.M. obtained resources and were the overall supervisors of this work.

## Supplemental information

Document S1. Figures S1–S6, Notes S1–S4, and supplemental experimental procedures

Dataset S1. RNA-seq and Hi-C dataset metrics, stratum adjusted correlation coefficients, proportions of overlapping TAD boundaries, and numbers of DEGs associated with PPI anchors, related to Figures 1, 2, and 4–6

Dataset S2. GO terms for PC loadings, analyses of differentially expressed genes (DEGs), DEGs associated with B-to-A and A-to-B compartment transitions, DEGs associated with shared TAD boundaries in stable A compartments, and DEGs associated with PPI anchors in A and B compartments, related to Figures 1, and 3–5

Document S2. Article plus supplemental information
