# Supplementary material for: Dynamic chromatin organization and regulatory interactions in human endothelial cell differentiation
Source: Stem Cell Reports. 2022 Dec 8;18(1):159–74. doi: 10.1016/j.stemcr.2022.11.003 (PMC9860068; doi:10.1016/j.stemcr.2022.11.003)
Supplement: Document S1. Figures S1–S6, Notes S1–S4, and supplemental experimental procedures [file mmc1.pdf]

Stem Cell Reports, Volume *18*

Supplemental Information

Dynamic chromatin organization and regulatory interactions in human endothelial cell differentiation

Kris G. Alavattam, Katie A. Mitzelfelt, Giancarlo Bonora, Paul A. Fields, Xiulan Yang, Han Sheng Chiu, Lil Pabon, Alessandro Bertero, Nathan J. Palpant, William S. Noble, and Charles E. Murry

Supplemental Information

Supplemental figures

Figure S1. Validation of stem cell line and the purity of endothelial cells, and transcriptomes are highly dynamic in endothelial cell specification, related to Figure 1

Figure S2. Insulation-score consistency indicates Hi-C data are of high quality, related to Figure 2

Figure S3. Dynamic compartmentalization reveals an endothelial cell differentiation trajectory that resembles the transcriptome-identified trajectory, related to Figure 3

Figure S4. In differentiation, TAD boundaries converge on an endothelial cell state, and gained boundaries tend to be associated with repressive chromatin environments, related to Figure 4

Figure S5. PPI anchors are enriched at sites of DEGs, and examples of PPIs associated with gene repression, related to Figure 5

Figure S6. Chromatin topologies differ in endothelial cells versus cardiomyocytes, related to Figure 6

Supplemental datasets

Dataset S1. RNA-seq and Hi-C dataset metrics, stratum-adjusted correlation coefficients, proportions of overlapping TAD boundaries, and numbers of DEGs associated with PPI anchors, related to Figures 1, 2, 4–6

Dataset S2. GO terms for PC loadings, analyses of differentially expressed genes (DEGs), DEGs associated with B-to-A and A-to-B compartment transitions, DEGs associated with shared TAD boundaries in stable A compartments, and DEGs associated with PPI anchors in A and B compartments, related to Figures 1, 3–5

Supplemental notes

Note S1. Transcriptomes undergo overt, cell type-relevant changes in endothelial cell differentiation, related to Figure 1

Note S2. Hi-C datasets are of high quality as indicated by read alignment metrics, analyses of insulation-score consistency, and evaluation of stratum-adjusted correlation coefficients, related to Figure 2

Note S3. In differentiation, endothelial cell genes associate with PPIs in both eu- and heterochromatic regions, related to Figure 5

Note S4. Additional comments and interpretation, related to Discussion

Supplemental experimental procedures

Cell culture

Flow cytometry

Hi-C: Sample preparation, library generation, and sequencing

RNA-seq: Sample preparation, library generation, and sequencing

Hi-C: Data-sourcing, alignment, processing, and quality control

Hi-C: Generation and visualization of Hi-C heatmaps

Hi-C: *Cis* contact-decay curve analyses

Hi-C: Genomic compartment analyses

Hi-C: Multidimensional scaling

Hi-C: Analyses of topologically associating domains

Hi-C: Enrichment of topologically associating domain boundaries with respect to genomic compartments

Hi-C: Analyses of pairwise point interactions

RNA-seq: Sourcing, alignment, and gene-level quantification of alignments

RNA-seq: Principal component analysis

RNA-seq: Gene expression analysis

RNA-seq: Differential gene expression analysis

RNA-seq: Gene Ontology analyses

RNA-seq: Enrichment of differentially expressed genes with respect to genomic compartments

RNA-seq: Enrichment of differentially expressed genes with respect to topologically associating domain boundaries

RNA-seq: Enrichment of differentially expressed genes with respect to pairwise point interactions

Statistics

Figure preparation

Supplemental references

Supplemental figures

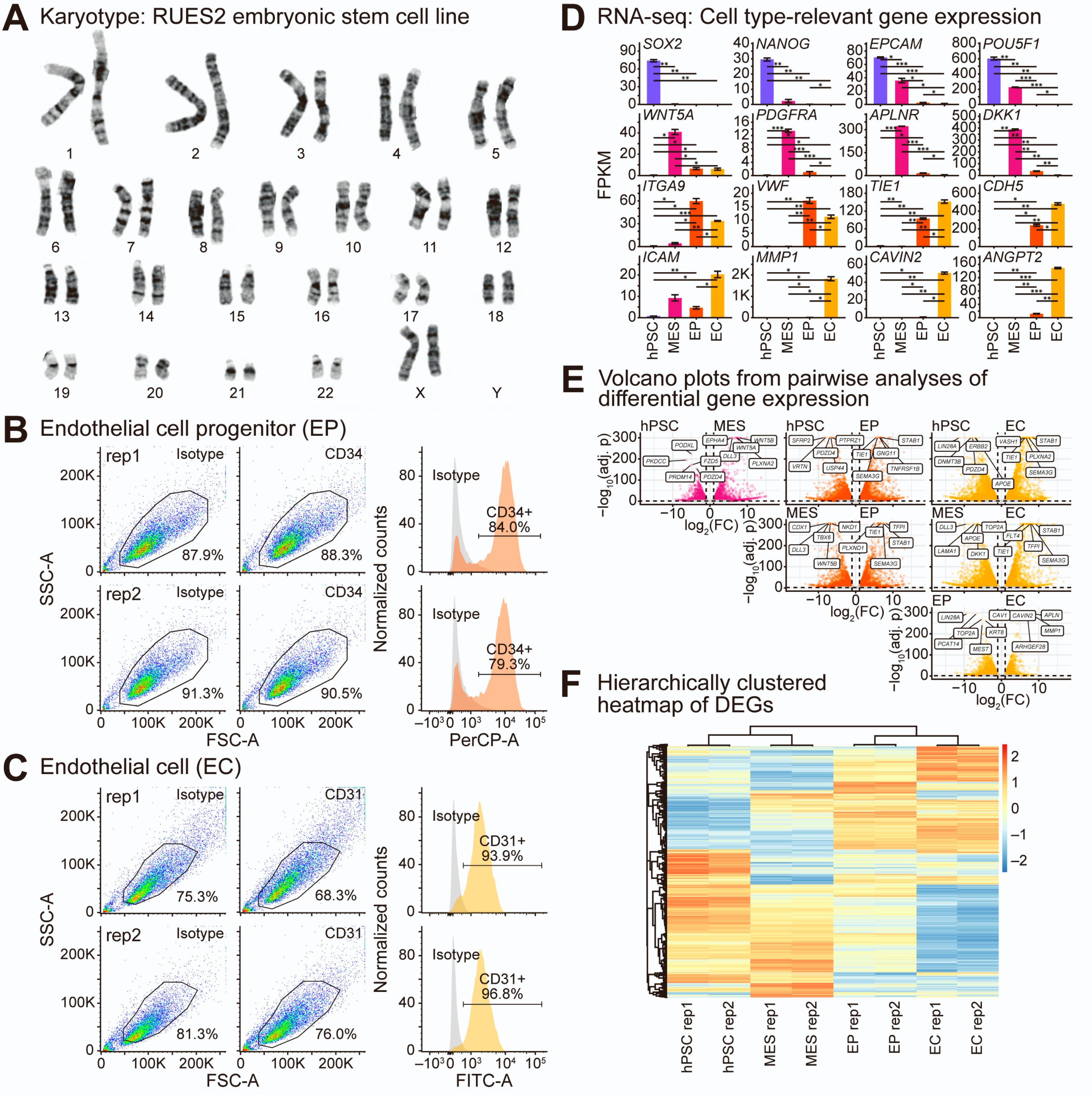
A Karyotype: RUES2 embryonic stem cell line
B Endothelial cell progenitor (EP)
C Endothelial cell (EC)
D RNA-seq: Cell type-relevant gene expression
E Volcano plots from pairwise analyses of differential gene expression
F Hierarchically clustered heatmap of DEGs
SSC-A
FSC-A
PerCP-A
FITC-A
Normalized counts
FPKM
hPSC
MES
EP
EC

Figure S1. Validation of stem cell line and the purity of endothelial cells, and transcriptomes are highly dynamic in endothelial cell specification, related to Figure 1

A. Karyotype analysis of RUES2 human embryonic stem cells demonstrating a normal 46,XX pattern.

B. Left: flow cytometry scatter plot for endothelial progenitor cells (EP) via immunostaining against human anti-IgG antibody, a negative control. Middle: flow cytometry scatter plot indicating EP purity via immunostaining against mouse anti-human CD34-PerCP antibody. Right: overlapping histograms for EP stained against the IgG isotype (gray) and CD34 (orange). Counts were normalized to modes. Top: replicate 1; bottom: replicate 2.

C. Left: flow cytometry scatter plot for endothelial cells (EC) as in panel **A**. Middle: flow cytometry scatter plot indicating EC purity via immunostaining against mouse anti-human CD31-FITC antibody. Right: overlapping histograms of EC stained against the IgG isotype (gray) and CD31 (yellow). Counts were normalized to modes. Top: replicate 1; bottom: replicate 2.

D. Bar charts for RNA-seq expression levels (FPKM) for genes relevant to time point-specific cell functions in endothelial cell differentiation. hPSC: human pluripotent stem cells; MES: mesoderm cells; EP: endothelial progenitor cells; EC: endothelial cells. P-values from pairwise t-tests between samples (two independent replicates each) adjusted with Benjamini-Hochberg post-hoc tests: * < 0.05, ** < 0.01, *** < 0.001. Bar: mean; error bars: standard error of the mean (SEM).

E. Volcano plots showing magnitude of change ($\log_2$ fold change, or “FC”) versus significance ($-\log_{10}$ adjusted p-values) for differentially expressed genes (DEGs) from pairwise DESeq2 analyses (Love et al., 2014) (adjusted p-value < 0.05, absolute $\log_2$ fold change > 1) of RNA-seq samples from endothelial cell differentiation: human pluripotent stem cells (hPSC) versus mesoderm cells (MES), endothelial progenitor cells (EP), and endothelial cells (EC; top row); MES versus EP and EC (middle row); and EP versus EC (bottom row). Labels: the top five down- and upregulated DEGs per analysis.

F. Hierarchically clustered heatmap of DEGs from pairwise analyses as in panel **A**. Color scale: $\log_2$-transformed, z score-normalized expression (FPKM).

A Spearman ρ for insulation scores

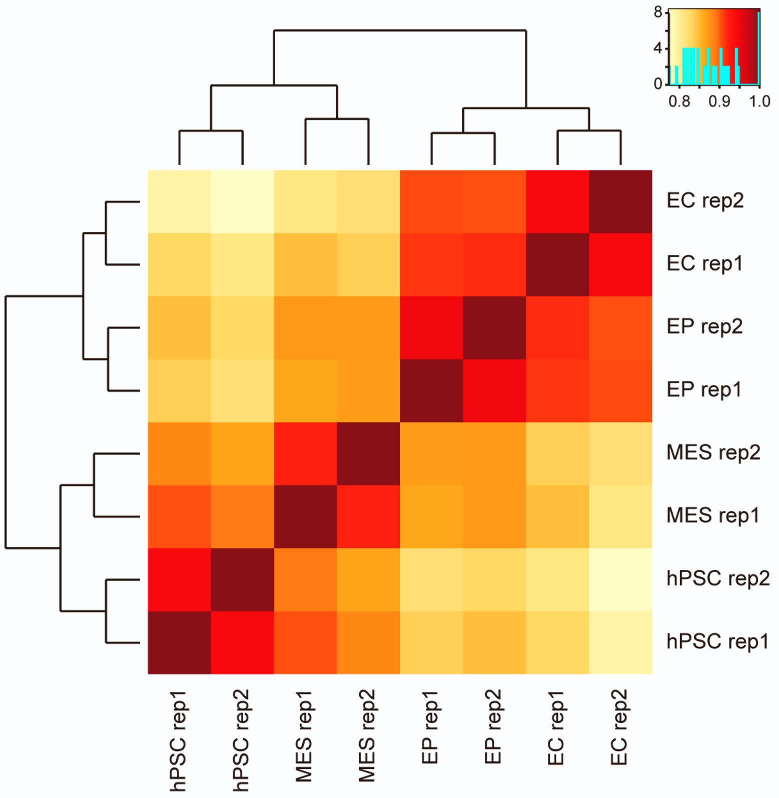

B Insulation score scatter plots

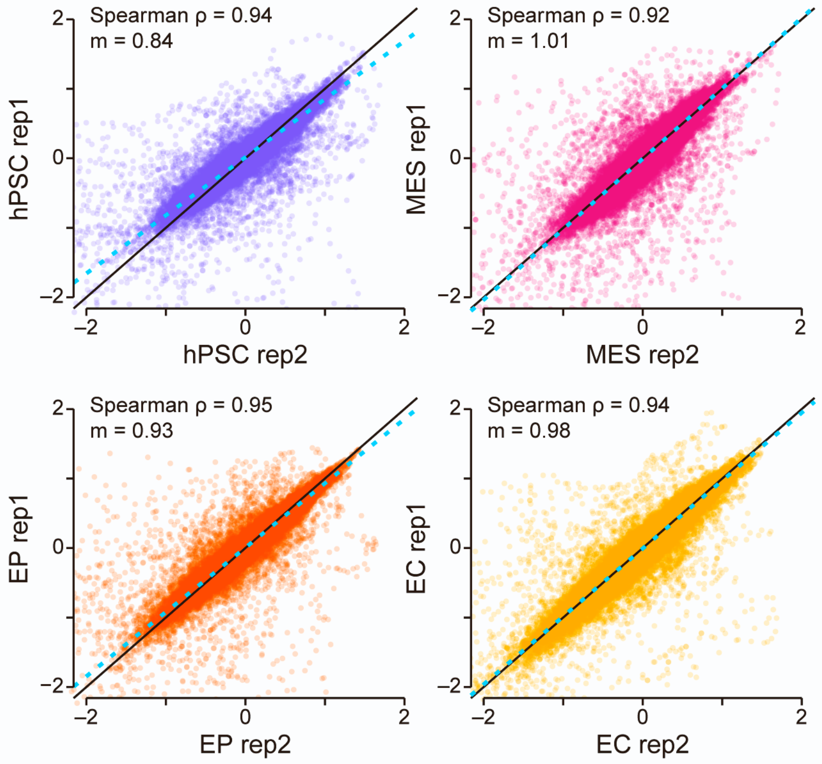

Figure S2. Insulation-score consistency indicates Hi-C data are of high quality, related to Figure 2

A. Hierarchically clustered heatmap of Spearman correlation coefficients (ρ) for insulation scores from Hi-C sample replicates (40-kb resolution, autosomes) taken across endothelial cell differentiation.

B. Scatter plots for Hi-C sample replicate (40-kb resolution, autosomes) insulation scores: hPSC (top left), MES (top right), EP (bottom left), and EC (bottom right). ρ: Spearman correlation coefficients; m: regression slope; blue dashed line: regression line; black solid line: x = y.

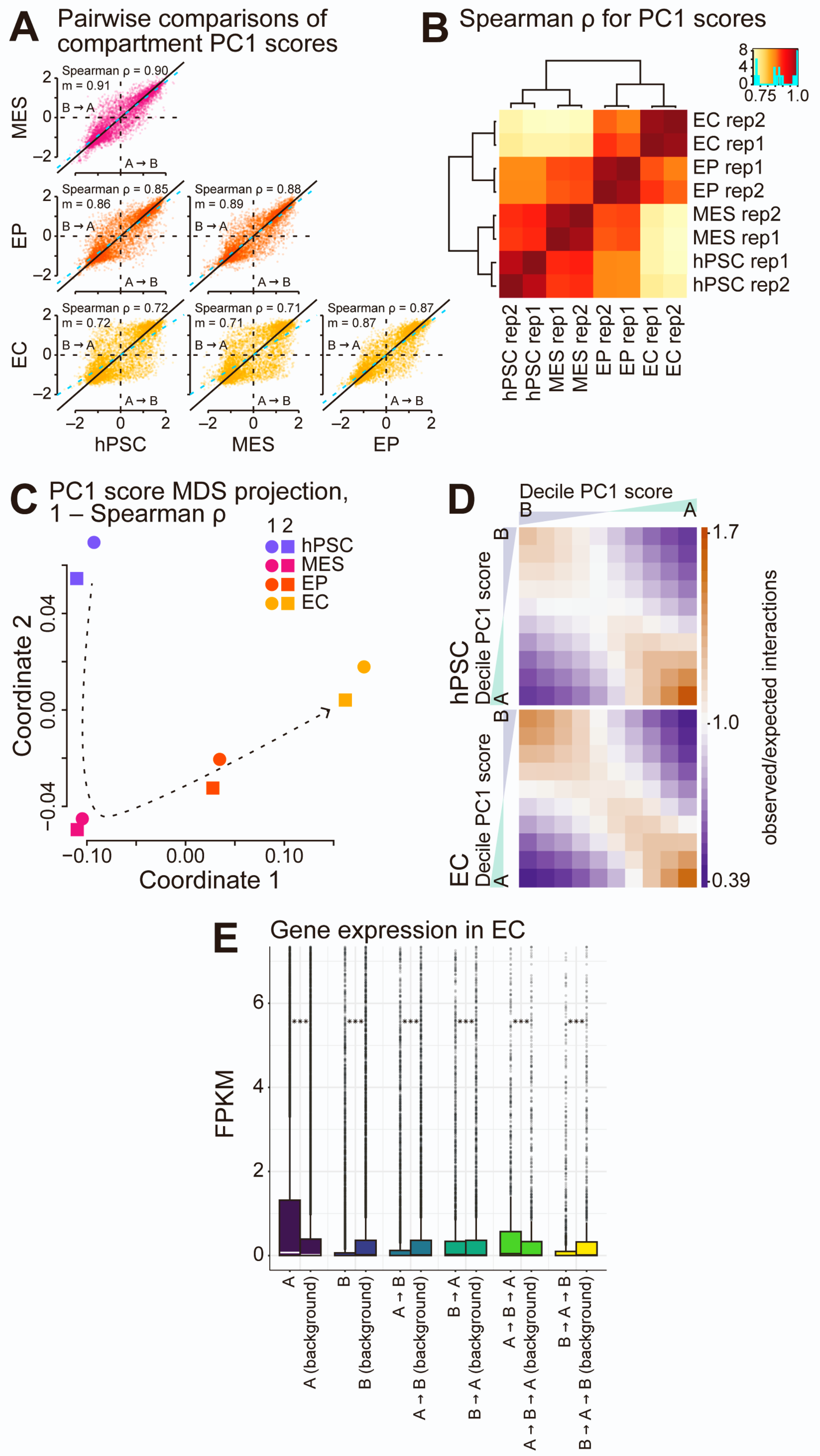

Alavattam, Mitzelfelt et al. – Figure S3

Figure S3. Dynamic compartmentalization reveals an endothelial cell differentiation trajectory that resembles the transcriptome-identified trajectory, related to Figure 3

A. Scatter plots for Hi-C sample (500-kb resolution, autosomes) PC1 scores: hPSC versus MES (top row), hPSC versus EP, MES versus EP (middle row); hPSC versus EC, MES versus EC, EP versus EC (bottom row). ρ: Spearman correlation coefficient; m: regression slope; blue dashed line: regression line; black solid line: x = y.

B. Hierarchically clustered heatmap of Spearman correlation coefficients (ρ) for PC1 scores from Hi-C samples (500-kb resolution, autosomes).

C. MDS projection of PC1 scores for Hi-C samples (500-kb resolution, autosomes); similarity measure: 1 – Spearman correlation coefficient (ρ). Arrow: differentiation trajectory.

D. Saddle plots for EC and hPSC Hi-C samples (500-kb resolution, autosomes). Gold-to-purple color bar: observed/expected interactions.

E. Box-and-whisker plots showing EC gene expression (FPKM; averaged from two independent replicates) in stable (A, B) and dynamic (A-to-B, B-to-A, A-to-B-to-A, B-to-A-to-B) compartments. Box-and-whisker plots represent the 25th percentile, median, and 75th percentile; whiskers extend to 1.5 times the interquartile range. P-values from pairwise Kolmogorov-Smirnov tests between observed and corresponding background distributions adjusted with Benjamini-Hochberg post-hoc tests: *** < 0.001.

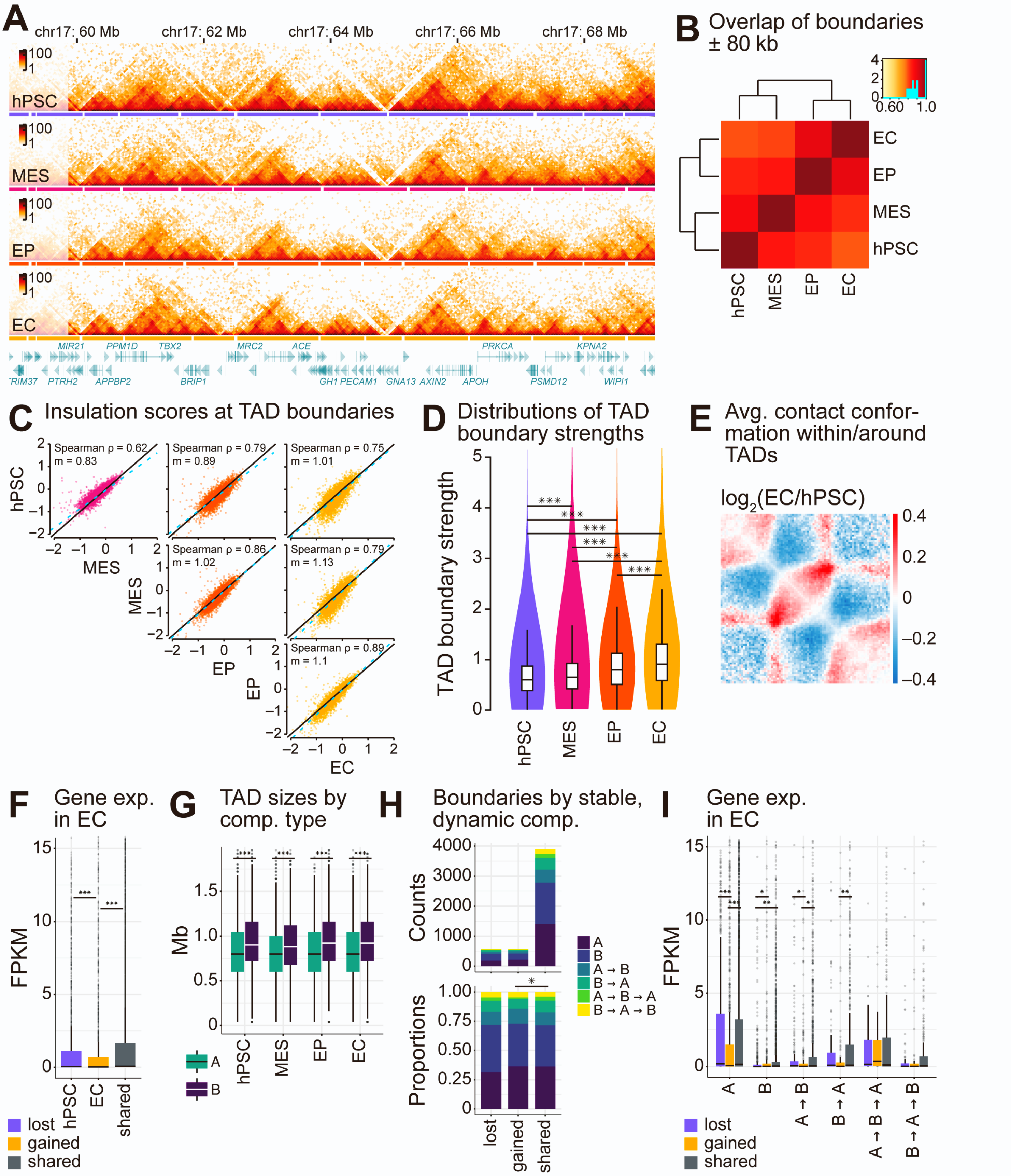

Alavattam, Mitzelfelt et al. – Figure S4

Figure S4. In differentiation, TAD boundaries converge on an endothelial cell state, and gained boundaries tend to be associated with repressive chromatin environments, related to Figure 4

A. Hi-C interaction heatmaps (40-kb bins, chromosome 17, approximately 59–69 Mb) showing dynamics of local interactions and TADs in hPSC, MES, EP, and EC. Horizontal solid bars: TADs; gaps in horizontal solid bars: TAD boundaries; bottom row: genes (green).

B. Hierarchically clustered heatmap for TAD-boundary set intersections for Hi-C samples using a window of ± 80 kb (40-kb resolution, autosomes).

C. Scatter plots for Hi-C sample (40-kb resolution, autosomes) insulation scores at TAD boundaries: hPSC versus MES, EP, and EC (top row); MES versus EP and EC (middle row); and EP versus EC (bottom row). ρ: Spearman correlation coefficients; m: regression slope; blue dashed line: regression line; black solid line: x = y.

D. Box-and-whisker plots imposed over violin plots showing TAD boundary strength distributions for Hi-C samples (40-kb resolution, autosomes). Box-and-whisker plots represent the 25th percentile, median, and 75th percentile; whiskers extend to 1.5 times the interquartile range. P-values from pairwise Kolmogorov-Smirnov tests adjusted with Benjamini-Hochberg post-hoc tests: * < 0.05, ** < 0.01, *** < 0.001.

E. $\log_2$ ratio heatmap for an EC aggregate TAD plot over an hPSC aggregate TAD plot (see Figure 3C); red: interaction frequency higher in EC; blue: interaction frequency higher in hPSC.

F. Box-and-whisker plots showing FPKM-normalized EC gene expression at TAD boundaries lost in differentiation (hPSC-specific), gained in differentiation (EC-specific), and shared between time points (hPSC and EC); FPKM values are averaged from two independent replicates. P-values from pairwise Kolmogorov-Smirnov tests adjusted with Benjamini-Hochberg post-hoc tests: *** < 0.001; if no asterisks, then not significant.

G. Box-and-whisker plots depicting TAD size distributions within A and B compartments across differentiation. P-values from intra-sample Kolmogorov-Smirnov tests adjusted with Benjamini-Hochberg post-hoc tests: *** < 0.001.

H. Stacked bar charts showing the absolute (top) and relative (bottom) numbers of lost, gained, and shared TAD boundaries stratified by stable (A, B) and dynamic (A-to-B, B-to-A, A-to-B-to-A, B-to-A-to-B) compartments. P-values from pairwise chi-squared contingency table tests: * < 0.05; if no asterisks, then not significant.

I. Box-and-whisker plots showing EC gene expression (FPKM; averaged from two independent replicates) distributions at lost, gained, and shared TAD boundaries stratified by stable and dynamic compartments. P-values from pairwise Kolmogorov-Smirnov tests and were adjusted with Benjamini-Hochberg post-hoc tests: * < 0.05, ** < 0.01, *** < 0.001; if no asterisks, then not significant.

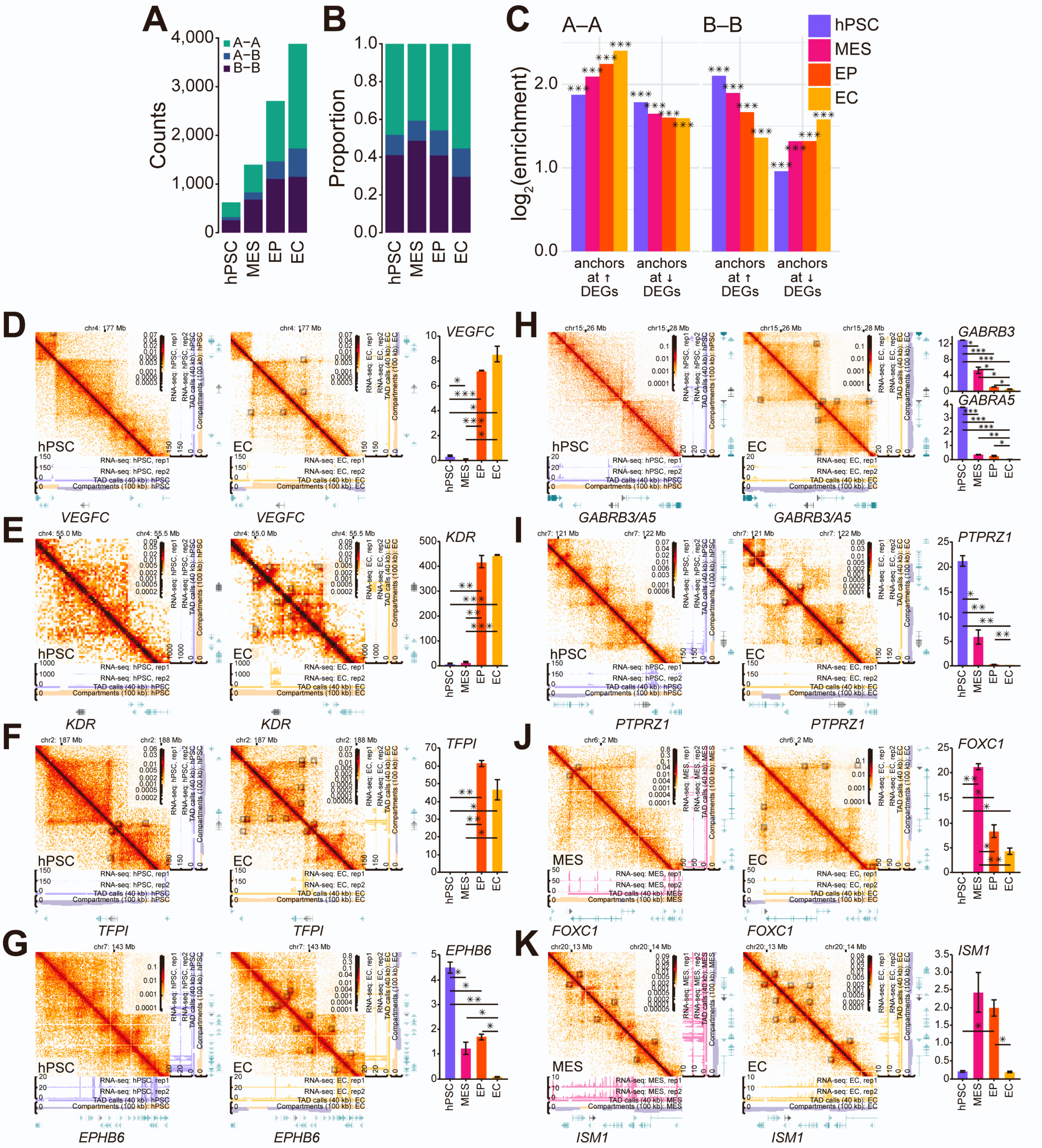

Alavattam, Mitzelfelt et al. – Figure S5

Figure S5. PPI anchors are enriched at sites of DEGs, and examples of PPIs associated with gene repression, related to Figure 5

A, B. Stacked bar charts showing the absolute (**A**) and relative (**B**) numbers of PPIs for Hi-C samples (10-kb resolution, autosomes). Bars are stratified by PPI-anchor compartment (100-kb resolution, autosomes) of origin; A–A: both anchors in A compartments; A–B: one anchor in A compartment, other anchor in B compartment; B–B, both anchors in B compartments.

C. Bar plots showing the $\log_2$ enrichment of PPI anchors at up- and downregulated differentially expressed genes (DEGs) stratified by compartment type in which anchors are found (A–A, A–B, and B–B; 100-kb resolution, autosomes) for Hi-C samples (10-kb resolution, autosomes). DEGs from DESeq2 analysis (Love et al., 2014) (adjusted p-value < 0.05, absolute $\log_2$ fold change > 1) of EC versus hPSC. Enrichment significantly different via chi-squared tests with Yates corrections: *** < 0.001.

D–K. Left: visualization of PPIs associated with *VEGFC* (**D**), *KDR* (**E**), *TFPI* (**F**) *EPHB6* (**G**), *GABRB3* and *GABRA5* (**H**), *PTPRZ1* (**I**), *FOXC1* (**J**), and *ISM1* (**K**) in hPSC or MES (left), and EC (right) Hi-C samples (10-kb bins, autosomes). Heatmaps of normalized Hi-C interaction frequencies (10-kb resolution), RNA-seq signal (unadjusted), TADs (40-kb resolution; see Methods), genomic compartments (100-kb resolution; gold: A compartment; purple: B compartment), and genes (green and black); solid squares overlying the heatmaps: PPIs. Right: bar plots for the RNA-seq expression levels (FPKM) of the above genes. P-values from pairwise t-tests between samples (two independent replicates each) adjusted with Benjamini-Hochberg post-hoc tests: * < 0.05, ** < 0.01, *** < 0.001. Bar: mean; error bars: standard error of the mean (SEM).

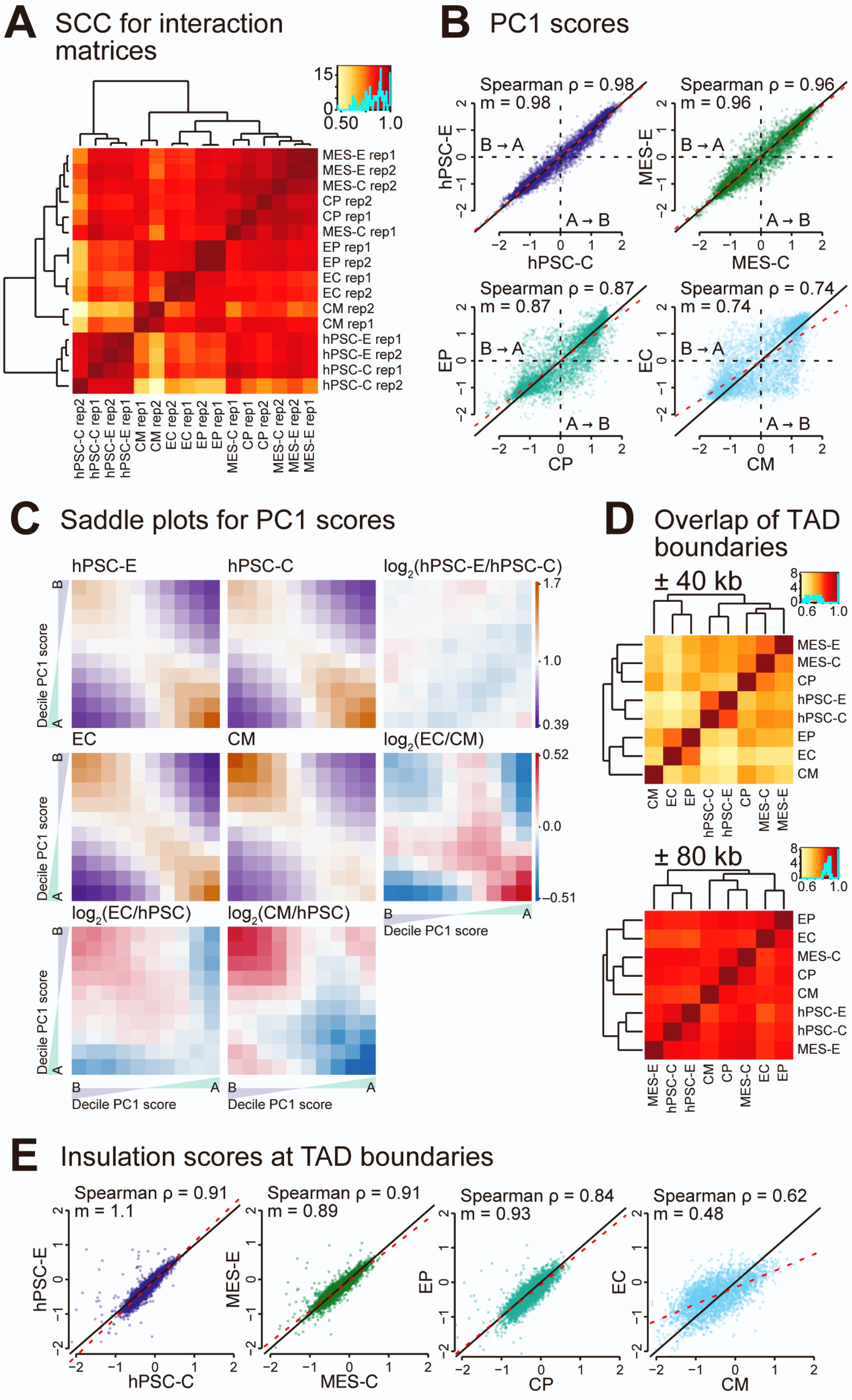

Alavattam, Mitzelfelt et al. – Figure S6

Figure S6. Chromatin topologies differ in endothelial cells versus cardiomyocytes, related to Figure 6

A. Hierarchically clustered heatmap of stratum-adjusted correlation coefficients (SCC) for Hi-C sample replicates (500-kb resolution, autosomes). hPSC-E: human pluripotent stem cells from endothelial cell differentiation; MES-E: mesoderm cells from endothelial cell differentiation; EP: endothelial progenitor cells ; EC: endothelial cells; hPSC-C: human pluripotent stem cells from cardiomyocyte differentiation; MES-C: mesoderm cells from cardiomyocyte differentiation; CP: cardiomyocyte progenitor cells; CM: cardiomyocytes.

B. Scatter plots for Hi-C sample (500-kb resolution, autosomes) PC1 scores from comparable time points in endothelial cell and cardiomyocyte differentiation: hPSC-E versus hPSC-C (top left), MES-E versus MES-C (top right), EP versus CP (bottom left), and EC versus CM (bottom right). ρ: Spearman correlation coefficient; m: regression slope; red dashed line: regression line; black solid line: x = y.

C. Saddle plots and $\log_2$ ratios of saddle plots for hPSC-E, hPSC-C, EC, and CM Hi-C samples (500-kb resolution, autosomes). Gold-to-purple color bar: observed/expected interactions. Red-to-blue color bar: $\log_2$ ratios of saddle plots; red: interactions higher in numerator; blue: interactions higher in denominator.

D. Hierarchically clustered heatmaps for TAD-boundary set intersections using windows of ± 40 kb (top) and ± 80 kb (bottom) around boundaries (Hi-C samples: 40-kb resolution, autosomes).

E. Scatter plots for Hi-C sample (40-kb resolution, autosomes) insulation scores from comparable time points in endothelial cell and cardiomyocyte differentiation: (left to right) hPSC-E versus hPSC-C, MES-E versus MES-C, EP versus CP, and EC versus CM. ρ: Spearman correlation coefficient; m: regression slope; red dashed line: regression line; black solid line: x = y.

Supplemental datasets

Dataset S1. RNA-seq and Hi-C dataset metrics, stratum-adjusted correlation coefficients, proportions of overlapping TAD boundaries, and numbers of DEGs associated with PPI anchors, related to Figures 1, 2, 4–6

- Tab 01 (related to Figure 1): Metrics for RNA-seq experiments.
- Tab 02 (related to Figure 2): Metrics for Hi-C experiments.
- Tab 03 (related to Figure 2): Stratum-adjusted correlation coefficients (SCCs) for endothelial cell Hi-C sample replicates (500-kb resolution, autosomes).
- Tab 04 (related to Figure 4): TAD boundary ± 40 kb (1 bin) intersections (top) and non-intersections (bottom) for endothelial cell Hi-C samples (40-kb resolution, autosomes).
- Tab 05 (related to Figure 4): Same as tab 04 except for TAD boundaries ± 80 kb (2 bins).
- Tab 06 (related to Figure 5): Counts for up- and downregulated DEGs associated with PPIs with anchors in A compartments, both compartments, and B compartments.
- Tab 07 (related to Figure 6): SCCs for both endothelial cell and cardiomyocyte Hi-C sample replicates.
- Tab 08 (related to Figure 6): Same as tab 04 except for both endothelial cell and cardiomyocyte Hi-C samples (40-kb resolution, autosomes).
- Tab 09 (related to Figure 6): Same as tab 08 except for TAD boundaries ± 80 kb.

Dataset S2. GO terms for PC loadings, analyses of differentially expressed genes (DEGs), DEGs associated with B-to-A and A-to-B compartment transitions, DEGs associated with shared TAD boundaries in stable A compartments, and DEGs associated with PPI anchors in A and B compartments, related to Figures 1, 3–5

Gene Ontology (GO) terms (molecular function, biological process, and cellular component) associated with the below data types. P-values from hypergeometric tests adjusted with Bonferroni corrections. List filtered for terms with adjusted p-value < 0.05.

- Tabs 01–04 (related to Figure 1): Top 500 PC1 negative loadings (tab 01), top 500 PC1 positive loadings (tab 02), top 500 PC2 negative loadings (tab 03), and top 500 PC2 positive loadings (tab 04)
- Tabs 05–16 (related to Figure 1): The up- and downregulated differentially expressed genes (DEGs) between…
    - mesoderm cells (MES) versus human pluripotent stem cells (hPSC; tabs 05, 06)
    - endothelial progenitor cells (EP) versus hPSC (tabs 07, 08)
    - endothelial cells (EC) versus hPSC (tabs 09, 10)
    - EP versus MES (tabs 11, 12)
    - EC versus MES (tabs 13, 14)
    - EC versus EP (tabs 15, 16)
- Tabs 17, 18 (related to Figure 3): The up- (tab 17) and downregulated (tab 18) DEGs between EC versus hPSC in regions that undergo, respectively, B-to-A and A-to-B compartment transitions
- Tabs 19, 20 (related to Figure 4): The up- (tab 19) and downregulated (tab 20) DEGs between EC versus hPSC in the vicinity (± 80 kb) of TAD boundaries in stable A compartments
- Tabs 21, 22 (related to Figure 5): The up- (tab 21) and downregulated (tab 22) DEGs between EC versus hPSC at, respectively, EC A–A and EC B–B PPI anchors

Supplemental notes

Note S1. Transcriptomes undergo overt, cell type-relevant changes in endothelial cell differentiation, related to Figure 1

We analyzed a panel of genes relevant to time point-specific cell functions and observed gene expression patterns consistent with endothelial specification (Figure S1D). Global analyses support this finding: Transcriptomes undergo overt changes in specification, with large amounts of differential expression (Figure S1E, F), and Gene Ontology (GO) analyses (Ashburner et al., 2000; Chen et al., 2009; Gene Ontology Consortium, 2021) of differentially expressed genes (DEGs) revealed biological processes highly relevant to corresponding cell types (Dataset S2). These results support the efficacy and reproducibility of our differentiation protocol, and underscore the dynamism of gene expression in endothelial cell development.

Note S2. Hi-C datasets are of high quality as indicated by read alignment metrics, analyses of insulation-score consistency, and evaluation of stratum-adjusted correlation coefficients, related to Figure 2

We observed the alignment of Hi-C reads at high rates (68–74%; Dataset S1) and high, comparable levels of *cis* interactions across differentiation (64–80%, Dataset S1). To further assess data quality, we calculated "insulation scores" (Crane et al., 2015) (Supplemental Experimental Procedures) for all replicates and compared these values within and between samples. Insulation scores are used to identify a form of chromatin organization known as "topologically associating domains" (discussed below). It is expected that topologically associating domains are harder to detect in noisy, low-quality datasets and, intuitively, such datasets should have a low degree of insulation-score consistency. Hierarchical clustering of Spearman correlation coefficients (ρ) for insulation scores showed high levels of concordance between replicates (Figure S2A); likewise, scatter plots revealed Spearman coefficients ≥0.92 and regression slopes ranging 0.84–1.01 (Figure S2B). Finally, we used the HiCRep method (Yang et al., 2017) to evaluate the consistency of biological replicates. Stratum-adjusted correlation coefficients (SCC) (Yang et al., 2017) revealed high levels of reproducibility between biological replicates (Dataset S1; Figure 2D, E). Together, these results indicate that our Hi-C datasets are of high quality.

Note S3. In differentiation, endothelial cell genes associate with PPIs in both eu- and heterochromatic regions, related to Figure 5

Examples of endothelial cell genes that associate with PPIs as their transcription increases in development include *VEGFC* (vascular endothelial growth factor C; Figure S5D), which codes for a protein critical for angiogenesis, endothelial cell growth, and blood vessel permeability in vascular and lymphatic vessels (Joukov et al., 1996; Jussila and Alitalo, 2002); *KDR* (kinase insert domain receptor; Figure S5E), which encodes a VEGF receptor (Terman et al., 1992); and *TFPI* (tissue factor pathway inhibitor; Figure S5F), a gene that encodes a serine protease inhibitor with anti-coagulative effects (Broze et al., 1990; Dahm et al., 2008; Ndonwi et al., 2010).

Numerous genes repressed in endothelial cell differentiation are associated with B-compartment PPIs, including genes associated with tissue patterning and neuronal development. These include *EPHB6* (ephrin type-B receptor 6; Figure S5G), which codes for a pseudokinase member of the Eph receptor family (Liang et al., 2021; Lisabeth et al., 2013; Nievergall et al., 2012; Wilkinson, 2014); *GABRB3* (gamma-aminobutyric acid type A receptor subunit beta 3) and *GABRA5* (gamma-aminobutyric acid type A receptor subunit alpha 3; Figure S5H), both of which encode receptor subunits for the neurotransmitter GABA; and *PTPRZ1* (protein tyrosine phosphatase receptor type Z1; Figure S5I), which codes for a

member of the receptor protein tyrosine phosphatase family that is largely restricted to the central nervous system in development (Wang et al., 2010).

Additionally, we observed examples of B-compartment PPI anchors associating with genes that code for factors with anti-angiogenic properties. Examples include *FOXC1* (forkhead box C1; Figure S5J), whose protein product has known antagonistic roles in vascular development (Koo and Kume, 2013); and *ISM1* (isthmin 1; Figure S5K), which codes for a secreted protein that functions as an endogenous angiogenesis inhibitor (Xiang et al., 2011).

Note S4. Additional comments and interpretation, related to Discussion

Contextualizing the increase in long-range cis contacts during endothelial cell differentiation. We observed a general, gross increase in long-range *cis* chromatin contacts as endothelial cells mature—a finding consistent with a number of studies of Hi-C (and Hi-C-like) data using *in vitro* and *in vivo* models of differentiation. These studies include stem cell differentiation to cells with neural identities (Bonev et al., 2017; Choi et al., 2020; Dixon et al., 2015; Fraser et al., 2015), studies of stem cell differentiation to cardiomyocytes (Bertero et al., 2019a, 2019b; Zhang et al., 2019), and studies of early mammalian development (Du et al., 2017; Ke et al., 2017), among others. However, in hematopoiesis, a form of cell lineage specification developmentally related to endothelial cell specification, there are decreases in the probabilities of long-range *cis* contacts in the development of megakaryocyte-erythrocyte progenitors from multipotent progenitors, megakaryocytes from megakaryocyte progenitors, and granulocytes from granulocyte-macrophage progenitors (Zhang et al., 2020). A drop in long-range *cis* contacts is also observed when germ cells mature into meiosis (Du et al., 2020; Wang et al., 2019), although long-range *cis* interactions increase as post-mitotic male germ cells mature (Alavattam et al., 2019; Vara et al., 2019; Wang et al., 2019). (Consistent with this, a marked drop occurs when proliferating cells enter into mitosis (Gibcus et al., 2018; Naumova et al., 2013).) Thus, the finding that there is an increase in long-range *cis* contacts during endothelial cell differentiation informs our collective assessment of the characteristics of differentiating cells.

TAD and PPI function is dependent on compartment type. While work remains to understand how TADs strengthen and PPIs arise in endothelial cells, our findings indicate that these features are influenced by stable and dynamic compartments to regulate transcription necessary for differentiation: strengthened TAD boundaries are accompanied by increased numbers of intra-TAD contacts, nascent TAD boundaries in A compartments are enriched for expressed genes and upregulated DEGs, and PPI anchors strongly correlate with upregulated DEGs in the A compartment, consistent with mounting evidence that loop-extruded features of chromatin organization play an important role in the regulation of gene expression (Bonev et al., 2017; Freire-Pritchett et al., 2017; Gorkin et al., 2014). We showed a number of examples of PPIs forming in A compartments coincident with the upregulation of genes essential to endothelial cell biology. On the other hand, PPI anchors and nascent TAD boundaries in B compartments are associated with lowly expressed and repressed genes, and downregulated DEGs. These findings indicate that the function of TADs and PPIs, two interrelated forms of chromatin organization, is contextual, dependent on compartment type; in stable and dynamic A compartments, they promote gene expression necessary for endothelial function.

Supplemental experimental procedures

Cell culture

To differentiate endothelial cells from human pluripotent stem cells, a modified version of a published protocol was followed (Palpant et al., 2015, 2017). Human pluripotent stem cells (hPSCs) from the RUES2 line (RUESe002-A; WiCell) were maintained on recombinant human Laminin-521 matrix (rhLaminin521; Biolamina) in Essential 8 (E8) media (ThermoFisher) at a density of 0.5 μg/cm^2. Cells were passaged with Versene (ThermoFisher) and seeded overnight with 10 μM Y-27632 (ROCK inhibitor; Tocris). Karyotyping was performed by Diagnostic Cytogenetics Incorporated, Seattle, WA, and cells were found to contain no clonal abnormalities (Figure S1A).

Prior to directed endothelial cell differentiation (day –1), hPSCs were re-seeded at a density of 3.0×10^5 cells per well of a 12-well plate coated with 2 μg/cm^2 rhLaminin521; then, the cells were immersed in E8 supplemented with 10 μM Y-27632. The following day (day 0), differentiation was induced with 7 μM CHIR99021 (a GSK3 inhibitor; Cayman) in RPMI (ThermoFisher) supplemented with 500 μg/mL BSA (Sigma, A9418) and 213 μg/mL ascorbic acid (Sigma, A8960). Seventy-two hours later (day 3), the media was switched to Stempro34 (Invitrogen) containing 300 ng/mL VEGF (Peprotech), 5 ng/mL bFGF (R&D), 10 ng/mL BMP4 (Preprotech), 4×10^{-4} M monothioglycerol (Sigma), 50 μg/mL ascorbic acid (Sigma), 2 mM L-glutamine (Invitrogen), and penicillin-streptomycin (Invitrogen). Forty-eight hours later (day 5), cells were passaged with 0.25% Trypsin (ThermoFisher) and re-seeded onto 0.2% gelatin-coated 10-cm dishes at a density of 5.0×10^5 cells per dish in Endothelial Cell Growth Medium (EGM; Lonza) containing 20 ng/mL VEGF, 20 ng/mL bFGF, and 1 μM CHIR99021. Cells were maintained on these dishes in EGM plus above-described factors until day 14 (Figure 1A).

Flow cytometry

To evaluate the purity of differentiating cells, cell aliquots were collected at days 6 and 14 of differentiation (Figure 1A). The cells were washed in Dulbecco's phosphate-buffered saline (DPBS) with 5% fetal bovine serum (FBS) and resuspended in a solution of Dulbecco's Modified Eagle's Medium (DMEM; Corning) containing mouse anti-human CD34-PerCP (BD Biosciences 340430, 1:5) and mouse anti-human CD31-FITC (BD Biosciences 555445, 1:5) antibodies, or a solution of DMEM and antibody-appropriate isotype controls. All staining was performed on live cells. Staining was performed on ice for 45 minutes in darkness. The stained cells were washed in DPBS and fixed with 4% paraformaldehyde (Affymetrix) prior to flow cytometry analyses. Flow cytometry was performed using a FACS Canto II cell analysis instrument (BD Biosciences); flow cytometry data were analyzed using FlowJo Software (FlowJo, LLC). Gates were set such that isotype controls contained 5% positive cells (Figure S1B, 1C).

Hi-C: Sample preparation, library generation, and sequencing

In situ DNase Hi-C (Ramani et al., 2016) was performed on 2–3 × 10^6 cells from two independent differentiations at the following time points: day 0, a pluripotent cell type (hPSC); day 2, a mesodermal cell type (MES); day 6, an endothelial progenitor cell type (EP); and day 14, an endothelial cell type (EC; Figure 1A). To prepare samples for Hi-C benchwork, plated cells were washed three times with DPBS and then fixed with a mixture of fresh RPMI containing 2% formaldehyde (diluted from a 37% formaldehyde solution); fixation took place for 10 minutes at room temperature with orbital rotation. Formaldehyde was quenched with 1% 2.5 M glycine for 5 minutes at room temperature and then 15 minutes at 4 °C. Afterwards, cells were treated with 0.25% trypsin for 5 minutes at 37 °C, washed with RPMI containing 10% FBS, and subsequently scraped off their plates. Cells were washed once with DPBS, flash frozen in liquid nitrogen, and stored at −80 °C until the time of Hi-C benchwork.

To perform *in situ* DNase Hi-C, frozen samples were thawed and lysed in 500 µL lysis buffer comprised of 10 mM Tris-HCl (pH 8.0), 10 mM NaCl, 0.2% Igepal CA-630, 1× protease inhibitor, and double-distilled water (ddH_2O). Then, nuclei were resuspended in 300 µL DNase buffer with 0.2% SDS and $MnCl_2$, and incubated at 37 °C for 60 minutes with periodic vortexing. Afterwards, nuclei were subjected to an additional 300 µL DNase buffer containing 2% Triton X-100 and RNase A, and incubated for another 10 minutes. Six units of DNase (ThermoFisher, EN0525) were added and incubated for 7 minutes at room temperature. The reaction was stopped with 30 µL of 0.5 M EDTA and 15 µL of 10% SDS. Nuclei were collected and resuspended in 150 µL water and combined with 300 µL AMPure XP beads (Beckman). DNA-end repair with T4 DNA Polymerase (ThermoFisher, EP0062) and Klenow (ThermoFisher, EP0052) was performed *in situ*, as was subsequent dA-tailing with Klenow Exo- (ThermoFisher, EP0422). Then, biotinylated oligonucleotides (adapters) were ligated to DNA at 16 °C overnight. To remove unbound adapters, nuclei were washed once with AMPure buffer (20% PEG in 2.5 M NaCl), then twice with 80% ethanol. To carry out adapter phosphorylation and ligation, PNK treatment was performed for 4 hours at room temperature. To de-crosslink the DNA, samples were treated with Proteinase K overnight at 62 °C. The next day, DNA precipitation was performed with 0.055 mg/mL glycogen, 10% volume 3 M NaOAc (pH 5.2), and 100% volume isopropanol for 2 hours at −80 °C. To purify the DNA, it was resuspended in 100 µL water and combined with 100 µL of AMPure beads. The pull-down of biotin adapter-containing DNA was performed using MyOne C1 Beads (ThermoFisher, 65001) for 30 minutes at room temperature with rotation. Afterwards, samples were washed four times with bind-and-wash buffer (5 mM Tris-HCl pH 8.0, 0.5 mM EDTA, 1 M NaCl, and 0.05% Tween-20) followed by two elution-buffer washes. On-bead DNA underwent end repair using the reagents in a Fast DNA End Repair Kit (ThermoFisher, K0771), and this was followed by dA-tailing using Klenow Exo- (ThermoFisher, EP0422); between each reaction, the DNA was washed four times with bind-and-wash buffer and twice with Tris-EDTA buffer.

Sequencing Y-adapters were ligated at room temperature for 60 minutes. To amplify the Hi-C libraries, the DNA underwent 10 PCR cycles using Kapa HiFi ReadyStart Master Mix (Roche, KK2602) with barcode-containing primers. Libraries were purified with 0.8× volumes of Ampure XP beads and quantified with a Qubit prior to sequencing. The libraries were paired-end sequenced using a NextSeq 500 (Illumina) in a high-output run with 150 cycles, 75 cycles for each end.

RNA-seq: Sample preparation, library generation, and sequencing

Cell samples from the same two independent differentiations were collected in Buffer RLT (QIAGEN) at the time points described above (Figure 1A). Samples were stored at –80 °C prior to RNA purification, which was performed with an RNeasy Mini Kit (QIAGEN) with on-column DNase digestion. RNA-seq libraries were prepared from total RNA (≥200 nucleotides in length) using the TruSeq Stranded Total RNA Ribo-Zero H/M/R kit (Illumina, RS-122-2201). Libraries were paired-end sequenced on a NextSeq 500 (Illumina) in a high-output run with 150 cycles, 75 cycles for each end.

Hi-C: Data-sourcing, alignment, processing, and quality control

In situ DNase Hi-C datasets for cardiomyocyte samples differentiated from RUES2 hPSCs were obtained from published work (GEO GSE106690) (Bertero et al., 2019a). As with the endothelial cell data generated for this study, the cardiomyocyte data are comprised of samples from two independent differentiations at the following time points: day 0, hPSC; day 2, MES; day 5, a cardiomyocyte progenitor cell type (CP); and day 14, a cardiomyocyte cell type (CM).

Reads were aligned to a *Homo sapiens* reference genome (Ensembl 83) with BWA-MEM (0.7.13-r1126) (Li, 2013; Li and Durbin, 2009) using default parameters, and each read-pair end was aligned individually. The Hi-C datasets exhibit high percentages of unique, paired alignments (Dataset S1). Primary alignments were extracted and sorted with Samtools (version 1.2) (Li et al., 2009). Then, the

alignments were processed with HiC-Pro (version 2.7.6) (Servant et al., 2015), filtering for MAPQ scores ≥ 30 and excluding read pairs that mapped within 1 kb of each other; PCR duplicates, defined as sequence matches with the exact same starts and ends, were excluded. HiC-Pro `allValidPairs` files and unbalanced matrices were generated at the following resolutions: 40, 100, and 500 kb.

Hi-C data quality were assessed with three metrics: proportions of *cis* interactions, insulation-score (described below) consistency, and contact-matrix similarity. Processed Hi-C data exhibited consistently high levels of *cis* interactions across differentiation: 64–80% (Dataset S1). Insulation score analyses of replicate samples revealed high levels of concordance between replicates (Figure S2A, B). To assess contact-matrix similarity, HiCRep analyses (Yang et al., 2017) were performed with the following parameters: `resol = 500000, ubr = 5000000, h = 1`; all other parameters were set to default values. HiCRep stratum-adjusted correlation coefficients revealed high levels of reproducibility between biological replicates (Dataset S1; Figures 2D, E).

To facilitate genomic binning at resolutions finer than 40 kb, biological replicates were merged using HiC-Pro, thereby increasing the sequencing depth for each sample. The merged Hi-C datasets are comprised of 111.4 million unique, valid read pairs for hPSC from endothelial cell differentiation; 120.0 million for MES from endothelial cell differentiation; 128.7 million for EP; 132.9 million for EC; 138.5 million for hPSC from cardiomyocyte differentiation; 143.3 million MES from cardiomyocyte differentiation; 161.6 million for CP; and 185.0 million for CM. HiC-Pro `allValidPairs` files and unbalanced matrices were generated for pooled replicates at the following resolutions: 10, 20, 40, 100, and 500 kb.

Using cooler (Abdennur and Mirny, 2019) and HiCExplorer (Ramírez et al.; Wolff et al., 2018, 2020) `hicConvertFormat`, HiC-Pro matrices were converted to the cooler format (`.cool`). Read pairs not aligned to autosomes or chromosome X were excluded from `.cool` files with HiCExplorer `hicAdjustMatrix`. Cooler-formatted matrices were balanced using Sinkhorn balancing (Sinkhorn and Knopp, 1967) such that the sum of every row and column is equal; to do so, the cooler `balance` command was called with default parameters.

Hi-C: Generation and visualization of Hi-C heatmaps

To generate and visualize chromatin-contact heatmaps, HiCExplorer `hicPlotMatrix` was used with contact matrices. To aid visualization, matrix values were log-transformed. To generate and visualize differential interactions between samples, matrices of $\log_2$ ratios matrices were generated with HiCExplorer `hicCompareMatrices` using `.cool` files from two datasets; the matrices of $\log_2$ ratios were plotted with `hicPlotMatrix`. To generate and visualize Pearson correlation coefficient heatmaps, contact matrices were input into HiCExplorer `hicPCA` for conversion to distance-normalized matrices (Lieberman-Aiden et al., 2009) (i.e., matrices taken from dividing observed interactions by expected interactions) and then Pearson correlation coefficient matrices; `hicPlotMatrix` was used to visualize the Pearson correlation coefficient matrices.

Hi-C: *Cis* contact-decay curve analyses

Cis contact-decay curves were generated by aggregating normalized counts as a function of distance at 500-kb intervals using HiCExplorer `hicPlotDistVsCounts`. Hi-C matrices were scaled to match the sum of the smallest matrix in the set. P-values were obtained from pairwise t-tests between samples (Figures 2C; Figure 6B) and were adjusted for false discovery rate with the Benjamini-Hochberg method.

Hi-C: Genomic compartment analyses

To calculate principal component (PC) scores for genomic compartment assignments, contact matrices were distance-normalized, transformed into Pearson correlation coefficient matrices, and eigen-decomposed using HOMER (Heinz et al., 2010; Lin et al., 2012). HiC-Pro `validPairs` files were

used as inputs. Eigenvectors were generated for contact matrices at 100- and 500-kb resolutions. Eigenvectors were assessed, and the first PC (PC1) was found to represent the genomic compartment profile (data not shown); subsequent principal components represented profiles distinct from genomic compartments (data not shown). Per convention (Lieberman-Aiden et al., 2009), PC1 orientation and binwise genomic-compartment assignments were based on biological features: bins with higher gene densities and increased enrichment for transcription were assigned to the "A" compartment type; all other bins were assigned to the "B" compartment type. Using oriented PC1 scores, sample similarity was evaluated via Spearman correlation coefficient and multidimensional scaling analyses (described below; Figure S3B, C; Figure 6D, E).

Genomic compartment transitions were defined using the following two criteria: (i) for a given bin, PC1 scores were available for each sample (i.e., no sample is represented by NA at that bin); and (ii) for a given bin, at least one sample had a mean PC1 score greater than 0 and at least one sample had a mean PC1 score less than 0. A-to-B-to-A-to-B and B-to-A-to-B-to-A transitions represented less than 1% of genomic compartment-switch regions (Figure 3B) and were combined, respectively, with A-to-B- and B-to-A-transitioning regions for downstream analyses.

Saddle plots were generated by ranking each bin, assigning it to its corresponding percentile value, and then dividing the genome into deciles. Each interaction (observed) was normalized to the average score at the corresponding distance for *cis* interactions (expected), then assigned to a decile pair based on the two bins. Plots represent the $\log_2$ average observed/expected values for pairs of deciles. Changes between one sample and another (e.g., EC/hPSC in Figure 3I) are represented by a $\log_2$ transformation of the quotient.

Hi-C: Multidimensional scaling

To perform multidimensional scaling (MDS) (Kruskal and Wish, 1977) of Hi-C interaction matrices, one minus the stratum-adjusted correlation coefficient (SCC), a statistic calculated by HiCRep (Yang et al., 2017), was used as input. To perform MDS of PC1 scores, one minus the Spearman correlation coefficient was used as input.

Hi-C: Analyses of topologically associating domains

Using the insulation score method (Crane et al., 2015), topologically associating domains (TADs) were called for Hi-C at 40-kb resolution. To do so, cworld (github.com/dekkerlab/cworld-dekker) `matrix2insulation.pl` was called with the following parameters: `--is 520001 --ids 320001 --ss 160001 --nt 0.01 --im mean`. Insulation scores are defined as the average number of chromatin contacts across a given bin, and TAD boundaries are called at local insulation score minima, which represent areas where the average number of chromatin contacts across a given bin are few (Crane et al., 2015). Aggregate TAD heatmaps were generated using FAN-C (Kruse et al., 2020) `fanc aggregate` with the following parameters: `--tads-imakaev --vmin 0.02 --vmax 0.075`. A given boundary was categorized as "shared" if its midpoint was observed at the same location ± 40 kb or 80 kb, i.e., within up to ± one or two bins of the boundary; if boundaries did not meet these conditions, then they were categorized as sample-specific and described as "lost" if unique to the sample from earlier in differentiation (e.g., hPSC) and "gained" if unique to the later sample (e.g., EC).

TADs were categorized as being in A or B compartments if they occupied one of the two compartment types (Figure S4G); if a given TAD overlapped multiple compartments, then it was assigned to the compartment type that it overlapped the most.

Hi-C: Enrichment of topologically associating domain boundaries with respect to genomic compartments

To calculate the enrichment of TAD boundary types (lost, gained, and shared) with respect to stable (A, B) and dynamic (A-to-B, B-to-A, A-to-B-to-A, B-to-A-to-B) compartment categories, the following calculation was performed:

$$\frac{\frac{x}{y}}{\frac{z}{a}},$$

where *x* is the number of a given boundary type within a given compartment category, *y* is the number of the given boundary type across all compartment categories, *z* is the number of all boundaries within the given compartment category, and *a* is the number of all boundaries across all compartment categories. P-values were obtained from chi-squared tests with Yates corrections.

Hi-C: Analyses of pairwise point interactions

Pairwise point interactions (PPIs) were called at 10-, 20-, and 40-kb resolutions using HiCCUPS (Rao et al., 2014), which was invoked with the following parameters: `-m 512 -r 10000,20000,40000 -k KR -f .1,.1,.1 -p 4,2,1 -i 8,4,2 -t 0.02,1.5,1.75,2 -d 20000,40000,80000 --cpu`. UpSet plots were generated to score the overlap of loop anchors among samples (Conway et al., 2017; Lex et al., 2014).

RNA-seq: Sourcing, alignment, and gene-level quantification of alignments

Reads were mapped to hg38 using HISAT2 (version 2.1.0) (Pertea et al., 2016) with default settings. The RNA-seq datasets exhibit high percentages of unique, paired alignments (Dataset S1). Files in `.sam` format were converted into coordinate-sorted `.bam` files using sambamba (version 0.6.6) (Tarasov et al., 2015) with default parameters. The program featureCounts from the Subread package (version 1.6.3) (Liao et al., 2019) was used to quantify gene expression levels with the following parameters specified: `-p -B -a gtf_file -t exon -g gene_id`.

RNA-seq: Principal component analysis

Principal component analysis (PCA) was performed with the software PCAtools (version 3.15; github.com/kevinblighe/PCAtools). An unfiltered RNA-seq counts matrix for the eight endothelial cell replicates—two independent replicates for each of four samples—was normalized with the DESeq2 function `rlog` (Love et al., 2014) prior to performing PCA. To determine significant principal components (PCs), Horn's parallel analysis (Horn, 1965) was performed using the PCAtools function `parallelPCA` with default settings. To determine the top positive and negative loading vectors for PCs 1 and 2, a PCAtools-generated loadings matrix was ordered by sign and magnitude prior to selecting the top 10 (Figure 1D) and top 500 (Figure 1E) component loadings.

RNA-seq: Gene expression analysis

To evaluate the expression of selected genes in endothelial cell differentiation (Figures 3–5; Figures S1, S5), the RNA-seq counts matrix was FPKM (fragments per kilobase of transcript per million mapped fragments)-normalized. Bar charts were plotted for genes of interest, and statistical significance was assigned through pairwise t-tests with false discovery rate adjustment (Benjamini-Hochberg method). Distributions of FPKM-normalized gene expression were plotted for genes associated with stable (A, B) and dynamic (B-to-A, A-to-B, A-to-B-to-A, B-to-A-to-B) compartment categories (Figure S3E); TAD boundary types, i.e., boundaries lost, gained, and shared in endothelial cell differentiation (Figure S4F); and TAD boundary types stratified by stable and dynamic compartment categories (Figure S4I).

Background distributions for FPKM-normalized gene expression were plotted for numbers of randomly sampled (non-repeating) genes equal to the numbers of genes in corresponding observed distributions (Figure S3E). P-values were obtained from pairwise Kolmogorov-Smirnov tests adjusted with Benjamini-Hochberg post-hoc tests; for Figure S3E, only p-values from comparisons between observed and background distributions are shown.

RNA-seq: Differential gene expression analysis

Differential gene expression analysis was performed with DESeq2 (version 1.32.0) (Love et al., 2014). Genes exhibiting a false discovery rate-adjusted p-value (Benjamini-Hochberg method) < 0.05 and an absolute $\log_2$ fold change > 1 were categorized as DEGs. EnhancedVolcano (version 1.14; github.com/kevinblighe/EnhancedVolcano) was used to generate labeled volcano plots (Figure S1E). A hierarchically clustered heatmap for $\log_2$ FPKM-normalized gene expression was generated for all non-redundant differentially expressed genes identified from pairwise analyses of endothelial cell samples (Figure S1F).

RNA-seq: Gene Ontology analyses

Gene Ontology (Ashburner et al., 2000; Chen et al., 2009; Gene Ontology Consortium, 2021) term enrichment analyses were performed using the ToppGene Suite ToppFun application (Chen et al., 2009); default settings were used, and the full gene set for each category was used as the background set. P-values were obtained from hypergeometric tests and adjusted for family-wise error rate with Bonferroni corrections.

RNA-seq: Enrichment of differentially expressed genes with respect to genomic compartments

To calculate enrichment scores for DEGs with respect to stable (A, B) and dynamic (A-to-B, B-to-A, A-to-B-to-A, B-to-A-to-B) compartment categories, the following calculation was performed:

$$\frac{\frac{x}{y}}{\frac{d}{g}},$$

where *x* is the number of DEGs within a given compartment category, *y* is the number of genes within the given compartment category, *d* is the number of all DEGs, and *g* is the number of all genes. P-values were obtained from chi-squared tests with Yates corrections.

RNA-seq: Enrichment of differentially expressed genes with respect to topologically associating domain boundaries

To calculate enrichment scores for DEGs with respect to TAD boundary types (lost, gained, and shared), the following calculation was performed:

$$\frac{\frac{x}{y}}{\frac{d}{g}},$$

where *x* is the number of DEGs within ± 80 kb (i.e., ± two bins) of a given boundary type, *y* is the number of genes within ± 80 kb of the given boundary type, *d* is the number of all DEGs, and *g* is the number of all genes.

To calculate enrichment scores for up- and downregulated DEGs with respect to TAD boundary types stratified by stable and dynamic compartment categories, the above calculation was performed except *x* is the number of up- or downregulated DEGs within ± 80 kb of a given boundary type within a

given compartment category, y is the number of up- or downregulated genes within ± 80 kb of the given boundary type within the given compartment category, d is the number of all up- or downregulated DEGs, and g is the number of all genes. P-values were obtained from chi-squared tests with Yates corrections.

RNA-seq: Enrichment of differentially expressed genes with respect to pairwise point interactions

To calculate enrichment scores for genes that overlap PPI anchors, the following calculation was performed:

$$\frac{\frac{x}{d}}{\frac{y}{g}},$$

where x is the number of DEGs overlapping anchors, d is the total number of DEGs, y is the number of genes overlapping anchors, and g is the total number of genes.

We used the following equation to calculate enrichment scores for anchors that overlap genes:

$$\frac{x}{a \cdot \frac{d}{g}},$$

where x is the number of anchors overlapping DEGs, a is the total number of anchors, d is the total number of DEGs, and g is the total number of genes. P-values were obtained from chi-squared tests with Yates corrections.

Statistics

Strategies for stratification, sampling, and enrichment are described in the following *Supplemental Experimental Procedures* subsections: for statistical analyses of features with respect to stratified genomic compartments (i.e., those that remain stable or undergo one of four transitions in differentiation), see *Hi-C: Genomic compartment analyses*; for statistical analyses of features with respect to stratified TAD boundaries (i.e., those that are lost, gained, and shared in differentiation), see *Hi-C: Analyses of topologically associating domains*; for statistical analyses of observed versus background gene expression distributions, see *RNA-seq: Gene expression analysis*; for statistical analyses of DEGs, see *RNA-seq: Differential gene expression analysis*; and for statistical analyses of Gene Ontology term enrichment, see *RNA-seq: Gene Ontology analyses*. Statistical analyses of enrichment of (a) general gene expression and (b) DEGs with respect to stratified genomic compartments, TAD boundary types, PPIs, and combinations thereof, are found in the following subsections: *RNA-seq: Gene expression analysis*, *RNA-seq: Enrichment of differentially expressed genes with respect to genomic compartments*, *RNA-seq: Enrichment of differentially expressed genes with respect to topologically associating domain boundaries*, and *RNA-seq: Enrichment of differentially expressed genes with respect to pairwise point interactions*.

Specific statistical tests are described in the *Results* and *Supplemental Experimental Procedures* sections, and figure and supplemental figure captions. An overview of statistical tests used in this study follows. Student's t-tests were performed for Figures 2C, 3G, 3H, 4I, 4J, 5F, 6B, S1D, and S5D–K; t-tests were adjusted for false discovery rate using the Benjamini-Hochberg method. Chi-squared tests with Yates corrections were performed for Figures 3D, 4D–F, 5C, S4H, and S5C. Kolmogorov-Smirnov tests were performed for Figures S3E, S4D, S4F, S4G, and S4I; Kolmogorov-Smirnov tests were adjusted for false discovery rate using the Benjamini-Hochberg method. Hypergeometric tests were performed for Figures 1E, 3E, 3F, 4G, 4H, 5D, and 5E; hypergeometric tests were adjusted for family-wise error rate with Bonferroni corrections.

Figure preparation

Plots were generated with, alone or in combination, Excel (version 16.60, Microsoft), base R (version 4.1), the R software package ggplot2 (version 3.3.4), and various plotting programs employed by the other software packages used in this study. Illustrator (version 26.0.2, Adobe) was used for composing figures.

Supplemental references

Abdennur, N., and Mirny, L.A. (2019). Cooler: scalable storage for Hi-C data and other genomically labeled arrays. Bioinformatics https://doi.org/10.1093/bioinformatics/btz540.

Alavattam, K.G., Maezawa, S., Sakashita, A., Khoury, H., Barski, A., Kaplan, N., and Namekawa, S.H. (2019). Attenuated chromatin compartmentalization in meiosis and its maturation in sperm development. Nat. Struct. Mol. Biol. *26*, 175–184.

Ashburner, M., Ball, C.A., Blake, J.A., Botstein, D., Butler, H., Cherry, J.M., Davis, A.P., Dolinski, K., Dwight, S.S., Eppig, J.T., et al. (2000). Gene ontology: tool for the unification of biology. The Gene Ontology Consortium. Nat. Genet. *25*, 25–29.

Bertero, A., Fields, P.A., Ramani, V., Bonora, G., Yardimci, G.G., Reinecke, H., Pabon, L., Noble, W.S., Shendure, J., and Murry, C.E. (2019a). Dynamics of genome reorganization during human cardiogenesis reveal an RBM20-dependent splicing factory. Nat. Commun. *10*, 1538.

Bertero, A., Fields, P.A., Smith, A.S.T., Leonard, A., Beussman, K., Sniadecki, N.J., Kim, D.-H., Tse, H.-F., Pabon, L., Shendure, J., et al. (2019b). Chromatin compartment dynamics in a haploinsufficient model of cardiac laminopathy. J. Cell Biol. *218*, 2919–2944.

Bonev, B., Mendelson Cohen, N., Szabo, Q., Fritsch, L., Papadopoulos, G.L., Lubling, Y., Xu, X., Lv, X., Hugnot, J.-P., Tanay, A., et al. (2017). Multiscale 3D Genome Rewiring during Mouse Neural Development. Cell *171*, 557–572.e24.

Broze, G.J., Jr, Girard, T.J., and Novotny, W.F. (1990). Regulation of coagulation by a multivalent Kunitz-type inhibitor. Biochemistry *29*, 7539–7546.

Chen, J., Bardes, E.E., Aronow, B.J., and Jegga, A.G. (2009). ToppGene Suite for gene list enrichment analysis and candidate gene prioritization. Nucleic Acids Research *37*, W305–W311. https://doi.org/10.1093/nar/gkp427.

Choi, W.-Y., Hwang, J.-H., Lee, J.-Y., Cho, A.-N., Lee, A.J., Jung, I., Cho, S.-W., Kim, L.K., and Kim, Y.-J. (2020). Chromatin Interaction Changes during the iPSC-NPC Model to Facilitate the Study of Biologically Significant Genes Involved in Differentiation. Genes *11*. https://doi.org/10.3390/genes11101176.

Conway, J.R., Lex, A., and Gehlenborg, N. (2017). UpSetR: an R package for the visualization of intersecting sets and their properties. Bioinformatics *33*, 2938–2940. https://doi.org/10.1093/bioinformatics/btx364.

Crane, E., Bian, Q., McCord, R.P., Lajoie, B.R., Wheeler, B.S., Ralston, E.J., Uzawa, S., Dekker, J., and Meyer, B.J. (2015). Condensin-driven remodelling of X chromosome topology during dosage compensation. Nature *523*, 240–244.

Dahm, A.E.A., Sandset, P.M., and Rosendaal, F.R. (2008). The association between protein S levels and anticoagulant activity of tissue factor pathway inhibitor type 1. J. Thromb. Haemost. *6*, 393–395.

Dixon, J.R., Jung, I., Selvaraj, S., Shen, Y., Antosiewicz-Bourget, J.E., Lee, A.Y., Ye, Z., Kim, A., Rajagopal, N., Xie, W., et al. (2015). Chromatin architecture reorganization during stem cell differentiation. Nature *518*, 331–336.

Du, Z., Zheng, H., Huang, B., Ma, R., Wu, J., Zhang, X., He, J., Xiang, Y., Wang, Q., Li, Y., et al. (2017). Allelic reprogramming of 3D chromatin architecture during early mammalian development. Nature *547*, 232–235.

Du, Z., Zheng, H., Kawamura, Y.K., Zhang, K., Gassler, J., Powell, S., Xu, Q., Lin, Z., Xu, K., Zhou, Q., et al. (2020). Polycomb Group Proteins Regulate Chromatin Architecture in Mouse Oocytes and Early

Embryos. Mol. Cell *77*, 825–839.e7.

Fraser, J., Ferrai, C., Chiariello, A.M., Schueler, M., Rito, T., Laudanno, G., Barbieri, M., Moore, B.L., Kraemer, D.C.A., Aitken, S., et al. (2015). Hierarchical folding and reorganization of chromosomes are linked to transcriptional changes in cellular differentiation. Mol. Syst. Biol. *11*, 852.

Freire-Pritchett, P., Schoenfelder, S., Várnai, C., Wingett, S.W., Cairns, J., Collier, A.J., García-Vílchez, R., Furlan-Magaril, M., Osborne, C.S., Fraser, P., et al. (2017). Global reorganisation of cis-regulatory units upon lineage commitment of human embryonic stem cells. Elife *6*. https://doi.org/10.7554/eLife.21926.

Gene Ontology Consortium (2021). The Gene Ontology resource: enriching a GOld mine. Nucleic Acids Res. *49*, D325–D334.

Gibcus, J.H., Samejima, K., Goloborodko, A., Samejima, I., Naumova, N., Nuebler, J., Kanemaki, M.T., Xie, L., Paulson, J.R., Earnshaw, W.C., et al. (2018). A pathway for mitotic chromosome formation. Science *359*, eaao6135.

Gorkin, D.U., Leung, D., and Ren, B. (2014). The 3D genome in transcriptional regulation and pluripotency. Cell Stem Cell *14*, 762–775.

Heinz, S., Benner, C., Spann, N., Bertolino, E., Lin, Y.C., Laslo, P., Cheng, J.X., Murre, C., Singh, H., and Glass, C.K. (2010). Simple Combinations of Lineage-Determining Transcription Factors Prime cis-Regulatory Elements Required for Macrophage and B Cell Identities. Molecular Cell *38*, 576–589. https://doi.org/10.1016/j.molcel.2010.05.004.

Horn, J.L. (1965). A rationale and test for the number of factors in factor analysis. Psychometrika 30, 179–185.

Joukov, V., Pajusola, K., Kaipainen, A., Chilov, D., Lahtinen, I., Kukk, E., Saksela, O., Kalkkinen, N., and Alitalo, K. (1996). A novel vascular endothelial growth factor, VEGF-C, is a ligand for the Flt4 (VEGFR-3) and KDR (VEGFR-2) receptor tyrosine kinases. The EMBO Journal *15*, 290–298. https://doi.org/10.1002/j.1460-2075.1996.tb00359.x.

Jussila, L., and Alitalo, K. (2002). Vascular growth factors and lymphangiogenesis. Physiol. Rev. *82*, 673–700.

Ke, Y., Xu, Y., Chen, X., Feng, S., Liu, Z., Sun, Y., Yao, X., Li, F., Zhu, W., Gao, L., et al. (2017). 3D Chromatin Structures of Mature Gametes and Structural Reprogramming during Mammalian Embryogenesis. Cell *170*, 367–381.e20.

Koo, H.-Y., and Kume, T. (2013). FoxC1-dependent regulation of vascular endothelial growth factor signaling in corneal avascularity. Trends Cardiovasc. Med. *23*, 1–4.

Kruse, K., Hug, C.B., and Vaquerizas, J.M. (2020). FAN-C: a feature-rich framework for the analysis and visualisation of chromosome conformation capture data. Genome Biology *21*. https://doi.org/10.1186/s13059-020-02215-9.

Kruskal J.B., and Wish M. (1977). Multidimensional Scaling. Sage Publications, Beverly Hills, CA.

Lex, A., Gehlenborg, N., Strobelt, H., Vuillemot, R., and Pfister, H. (2014). UpSet: Visualization of Intersecting Sets. IEEE Transactions on Visualization and Computer Graphics *20*, 1983–1992. https://doi.org/10.1109/tvcg.2014.2346248.

Li, H. (2013). Aligning sequence reads, clone sequences and assembly contigs with BWA-MEM. https://doi.org/10.48550/ARXIV.1303.3997.

Li, H., and Durbin, R. (2009). Fast and accurate short read alignment with Burrows-Wheeler transform.

Bioinformatics *25*, 1754–1760. https://doi.org/10.1093/bioinformatics/btp324.

Li, H., Handsaker, B., Wysoker, A., Fennell, T., Ruan, J., Homer, N., Marth, G., Abecasis, G., Durbin, R., and 1000 Genome Project Data Processing Subgroup (2009). The Sequence Alignment/Map format and SAMtools. Bioinformatics *25*, 2078–2079.

Liang, L.-Y., Roy, M., Horne, C.R., Sandow, J.J., Surudoi, M., Dagley, L.F., Young, S.N., Dite, T., Babon, J.J., Janes, P.W., et al. (2021). The intracellular domains of the EphB6 and EphA10 receptor tyrosine pseudokinases function as dynamic signalling hubs. Biochem. J *478*, 3351–3371.

Liao, Y., Smyth, G.K., and Shi, W. (2019). The R package Rsubread is easier, faster, cheaper and better for alignment and quantification of RNA sequencing reads. Nucleic Acids Research *47*, e47–e47. https://doi.org/10.1093/nar/gkz114.

Lieberman-Aiden, E., van Berkum, N.L., Williams, L., Imakaev, M., Ragoczy, T., Telling, A., Amit, I., Lajoie, B.R., Sabo, P.J., Dorschner, M.O., et al. (2009). Comprehensive mapping of long-range interactions reveals folding principles of the human genome. Science *326*, 289–293.

Lin, Y.C., Benner, C., Mansson, R., Heinz, S., Miyazaki, K., Miyazaki, M., Chandra, V., Bossen, C., Glass, C.K., and Murre, C. (2012). Global changes in the nuclear positioning of genes and intra- and interdomain genomic interactions that orchestrate B cell fate. Nature Immunology *13*, 1196–1204. https://doi.org/10.1038/ni.2432.

Lisabeth, E.M., Falivelli, G., and Pasquale, E.B. (2013). Eph Receptor Signaling and Ephrins. Cold Spring Harbor Perspectives in Biology *5*, a009159–a009159. https://doi.org/10.1101/cshperspect.a009159.

Love, M.I., Huber, W., and Anders, S. (2014). Moderated estimation of fold change and dispersion for RNA-seq data with DESeq2. Genome Biol. *15*, 550.

Naumova, N., Imakaev, M., Fudenberg, G., Zhan, Y., Lajoie, B.R., Mirny, L.A., and Dekker, J. (2013). Organization of the mitotic chromosome. Science *342*, 948–953.

Ndonwi, M., Tuley, E.A., and Broze, G.J. (2010). The Kunitz-3 domain of TFPI-α is required for protein S–dependent enhancement of factor Xa inhibition. Blood *116*, 1344–1351.

Nievergall, E., Lackmann, M., and Janes, P.W. (2012). Eph-dependent cell-cell adhesion and segregation in development and cancer. Cell. Mol. Life Sci. *69*, 1813–1842.

Palpant, N.J., Pabon, L., Roberts, M., Hadland, B., Jones, D., Jones, C., Moon, R.T., Ruzzo, W.L., Bernstein, I., Zheng, Y., et al. (2015). Inhibition of β-catenin signaling respecifies anterior-like endothelium into beating human cardiomyocytes. Development *142*, 3198–3209.

Palpant, N.J., Pabon, L., Friedman, C.E., Roberts, M., Hadland, B., Zaunbrecher, R.J., Bernstein, I., Zheng, Y., and Murry, C.E. (2017). Generating high-purity cardiac and endothelial derivatives from patterned mesoderm using human pluripotent stem cells. Nat. Protoc. *12*, 15–31.

Pertea, M., Kim, D., Pertea, G.M., Leek, J.T., and Salzberg, S.L. (2016). Transcript-level expression analysis of RNA-seq experiments with HISAT, StringTie and Ballgown. Nature Protocols *11*, 1650–1667. https://doi.org/10.1038/nprot.2016.095.

Ramani, V., Cusanovich, D.A., Hause, R.J., Ma, W., Qiu, R., Deng, X., Blau, C.A., Disteche, C.M., Noble, W.S., Shendure, J., et al. (2016). Mapping 3D genome architecture through in situ DNase Hi-C. Nat. Protoc. *11*, 2104–2121.

Ramírez, F., Bhardwaj, V., Villaveces, J., Arrigoni, L., Grüning, B.A., Lam, K.C., Habermann, B., Akhtar, A., and Manke, T. High-resolution TADs reveal DNA sequences underlying genome organization in flies. https://doi.org/10.1101/115063.

Rao, S.S.P., Huntley, M.H., Durand, N.C., Stamenova, E.K., Bochkov, I.D., Robinson, J.T., Sanborn, A.L., Machol, I., Omer, A.D., Lander, E.S., et al. (2014). A 3D Map of the Human Genome at Kilobase Resolution Reveals Principles of Chromatin Looping. Cell *159*, 1665–1680. https://doi.org/10.1016/j.cell.2014.11.021.

Servant, N., Varoquaux, N., Lajoie, B.R., Viara, E., Chen, C.-J., Vert, J.-P., Heard, E., Dekker, J., and Barillot, E. (2015). HiC-Pro: an optimized and flexible pipeline for Hi-C data processing. Genome Biology *16*. https://doi.org/10.1186/s13059-015-0831-x.

Sinkhorn, R., and Knopp, P. (1967). Concerning nonnegative matrices and doubly stochastic matrices. Pacific Journal of Mathematics *21*, 343–348. https://doi.org/10.2140/pjm.1967.21.343.

Tarasov, A., Vilella, A.J., Cuppen, E., Nijman, I.J., and Prins, P. (2015). Sambamba: fast processing of NGS alignment formats. Bioinformatics *31*, 2032–2034. https://doi.org/10.1093/bioinformatics/btv098.

Terman, B.I., Dougher-Vermazen, M., Carrion, M.E., Dimitrov, D., Armellino, D.C., Gospodarowicz, D., and Böhlen, P. (1992). Identification of the KDR tyrosine kinase as a receptor for vascular endothelial cell growth factor. Biochem. Biophys. Res. Commun. *187*, 1579–1586.

Vara, C., Paytuví-Gallart, A., Cuartero, Y., Le Dily, F., Garcia, F., Salvà-Castro, J., Gómez-H, L., Julià, E., Moutinho, C., Aiese Cigliano, R., et al. (2019). Three-Dimensional Genomic Structure and Cohesin Occupancy Correlate with Transcriptional Activity during Spermatogenesis. Cell Rep. *28*, 352–367.e9.

Wang, V., Davis, D.A., Veeranna, R.P., Haque, M., and Yarchoan, R. (2010). Characterization of the activation of protein tyrosine phosphatase, receptor-type, Z polypeptide 1 (PTPRZ1) by hypoxia inducible factor-2 alpha. PLoS One *5*, e9641.

Wang, Y., Wang, H., Zhang, Y., Du, Z., Si, W., Fan, S., Qin, D., Wang, M., Duan, Y., Li, L., et al. (2019). Reprogramming of Meiotic Chromatin Architecture during Spermatogenesis. Mol. Cell *73*, 547–561.e6.

Wilkinson, D.G. (2014). Regulation of cell differentiation by Eph receptor and ephrin signaling. Cell Adh. Migr. *8*, 339–348.

Wolff, J., Bhardwaj, V., Nothjunge, S., Richard, G., Renschler, G., Gilsbach, R., Manke, T., Backofen, R., Ramírez, F., and Grüning, B.A. (2018). Galaxy HiCExplorer: a web server for reproducible Hi-C data analysis, quality control and visualization. Nucleic Acids Research *46*, W11–W16. https://doi.org/10.1093/nar/gky504.

Wolff, J., Rabbani, L., Gilsbach, R., Richard, G., Manke, T., Backofen, R., and Grüning, B.A. (2020). Galaxy HiCExplorer 3: a web server for reproducible Hi-C, capture Hi-C and single-cell Hi-C data analysis, quality control and visualization. Nucleic Acids Research *48*, W177–W184. https://doi.org/10.1093/nar/gkaa220.

Xiang, W., Ke, Z., Zhang, Y., Cheng, G.H.-Y., Irwan, I.D., Sulochana, K.N., Potturi, P., Wang, Z., Yang, H., Wang, J., et al. (2011). Isthmin is a novel secreted angiogenesis inhibitor that inhibits tumour growth in mice. J. Cell. Mol. Med. *15*, 359–374.

Yang, T., Zhang, F., Yardımcı, G.G., Song, F., Hardison, R.C., Noble, W.S., Yue, F., and Li, Q. (2017). HiCRep: assessing the reproducibility of Hi-C data using a stratum-adjusted correlation coefficient. Genome Res. *27*, 1939–1949.

Zhang, C., Xu, Z., Yang, S., Sun, G., Jia, L., Zheng, Z., Gu, Q., Tao, W., Cheng, T., Li, C., et al. (2020). tagHi-C Reveals 3D Chromatin Architecture Dynamics during Mouse Hematopoiesis. Cell Rep. *32*, 108206.

Zhang, Y., Li, T., Preissl, S., Amaral, M.L., Grinstein, J.D., Farah, E.N., Destici, E., Qiu, Y., Hu, R., Lee, A.Y., et al. (2019). Transcriptionally active HERV-H retrotransposons demarcate topologically associating domains in human pluripotent stem cells. Nat. Genet. *51*, 1380–1388.
