# Supplementary material for: Dynamic chromatin organization and regulatory interactions in human endothelial cell differentiation
Source: Stem Cell Reports. 2022 Dec 8;18(1):159–74. doi: 10.1016/j.stemcr.2022.11.003 (PMC9860068; doi:10.1016/j.stemcr.2022.11.003)
Supplement: Document S2. Article plus supplemental information [file mmc4.pdf]

Stem Cell Reports
Article
OPEN ACCESS

Dynamic chromatin organization and regulatory interactions in human endothelial cell differentiation

Kris G. Alavattam,[1,2,3,4,13] Katie A. Mitzelfelt,[1,2,3,13] Giancarlo Bonora,[4] Paul A. Fields,[1,2,3] Xiulan Yang,[1,2,3] Han Sheng Chiu,[5,6] Lil Pabon,[1,2,3,7] Alessandro Bertero,[1,2,3,12] Nathan J. Palpant,[5,6,8] William S. Noble,[4,9,*] and Charles E. Murry[1,2,3,7,10,11,*]

[1]Department of Laboratory Medicine and Pathology, University of Washington, 1959 NE Pacific Street, Seattle, WA 98195, USA
[2]Center for Cardiovascular Biology, University of Washington, 850 Republican Street, Brotman Building, Seattle, WA 98109, USA
[3]Institute for Stem Cell and Regenerative Medicine, University of Washington, 850 Republican Street, Seattle, WA 98109, USA
[4]Department of Genome Sciences, University of Washington, William H. Foege Hall, 3720 15th Avenue NE, Seattle, WA 98195, USA
[5]Institute for Molecular Bioscience, The University of Queensland, Brisbane, QLD 4072, Australia
[6]Centre for Cardiac and Vascular Biology, The University of Queensland, Brisbane, QLD 4072, Australia
[7]Sana Biotechnology, Seattle, WA 98102, USA
[8]School of Biomedical Sciences, The University of Queensland, Brisbane, QLD 4072, Australia
[9]Paul G. Allen School of Computer Science and Engineering, University of Washington, Seattle, WA 98195, USA
[10]Department of Medicine/Cardiology, University of Washington, 1959 NE Pacific Street, Seattle, WA 98195, USA
[11]Department of Bioengineering, University of Washington, 1959 NE Pacific Street, Seattle, WA 98195, USA
[12]Present address: Molecular Biotechnology Center, Department of Molecular Biotechnology and Health Sciences, University of Torino, Via Nizza 52, 10126 Torino, Italy
[13]These authors contributed equally
*Correspondence: wnoble@uw.edu (W.S.N.), murry@uw.edu (C.E.M.)
https://doi.org/10.1016/j.stemcr.2022.11.003

SUMMARY

Vascular endothelial cells are a mesoderm-derived lineage with many essential functions, including angiogenesis and coagulation. The gene-regulatory mechanisms underpinning endothelial specialization are largely unknown, as are the roles of chromatin organization in regulating endothelial cell transcription. To investigate the relationships between chromatin organization and gene expression, we induced endothelial cell differentiation from human pluripotent stem cells and performed Hi-C and RNA-sequencing assays at specific time points. Long-range intrachromosomal contacts increase over the course of differentiation, accompanied by widespread heteroeuchromatic compartment transitions that are tightly associated with transcription. Dynamic topologically associating domain boundaries strengthen and converge on an endothelial cell state, and function to regulate gene expression. Chromatin pairwise point interactions (DNA loops) increase in frequency during differentiation and are linked to the expression of genes essential to vascular biology. Chromatin dynamics guide transcription in endothelial cell development and promote the divergence of endothelial cells from cardiomyocytes.

INTRODUCTION

Endothelial cells, a mesoderm-derived cell population, line the entirety of the circulatory system. Their functions are complex and critical, including angiogenesis, blood clotting, barrier function, vasomotor function, and fluid/nutrient filtration. Endothelial cell dysfunction is a prominent feature of many pathological conditions, including nearly all cardiovascular diseases (Rajendran et al., 2013). Complex transcriptional changes mediate both endothelial cell development and dysfunction (De Val and Black, 2009; Xu, 2014). It remains largely unknown what brings about such changes in gene expression.

The role of 3D chromatin organization in gene expression is an active area of study. However, to date, few studies have examined the genome organization of endothelial cells (Niskanen et al., 2018; Rao et al., 2014), and none have made use of a model for endothelial cell differentiation in which all samples are clonal, having been differentiated from the same source. To fill this gap, we performed a modified version of a previously developed protocol to induce endocardial-like endothelial differentiation from human pluripotent stem cells (hPSCs; Palpant et al., 2015, 2017) and, taking advantage of *in situ* DNase Hi-C (Ramani et al., 2016), a form of high-throughput chromosome conformation capture with sequencing, we investigated global chromatin organization at specific time points in endothelial cell development. In performing RNA sequencing (RNA-seq), we correlated and contextualized Hi-C data with endothelial cell gene expression. Our results show that dynamic changes in chromatin organization, including genomic compartmentalization and topologically associating domains, are associated with changes in transcription. DNA loops (pairwise point interactions) are associated with the expression of essential genes in euchromatic genomic compartments. Altogether, this study provides a comprehensive look at dynamic 3D chromatin organization during human endothelial cell development and uncovers important relationships between 3D chromatin organization and transcription.

This is an open access article under the CC BY license (http://creativecommons.org/licenses/by/4.0/).

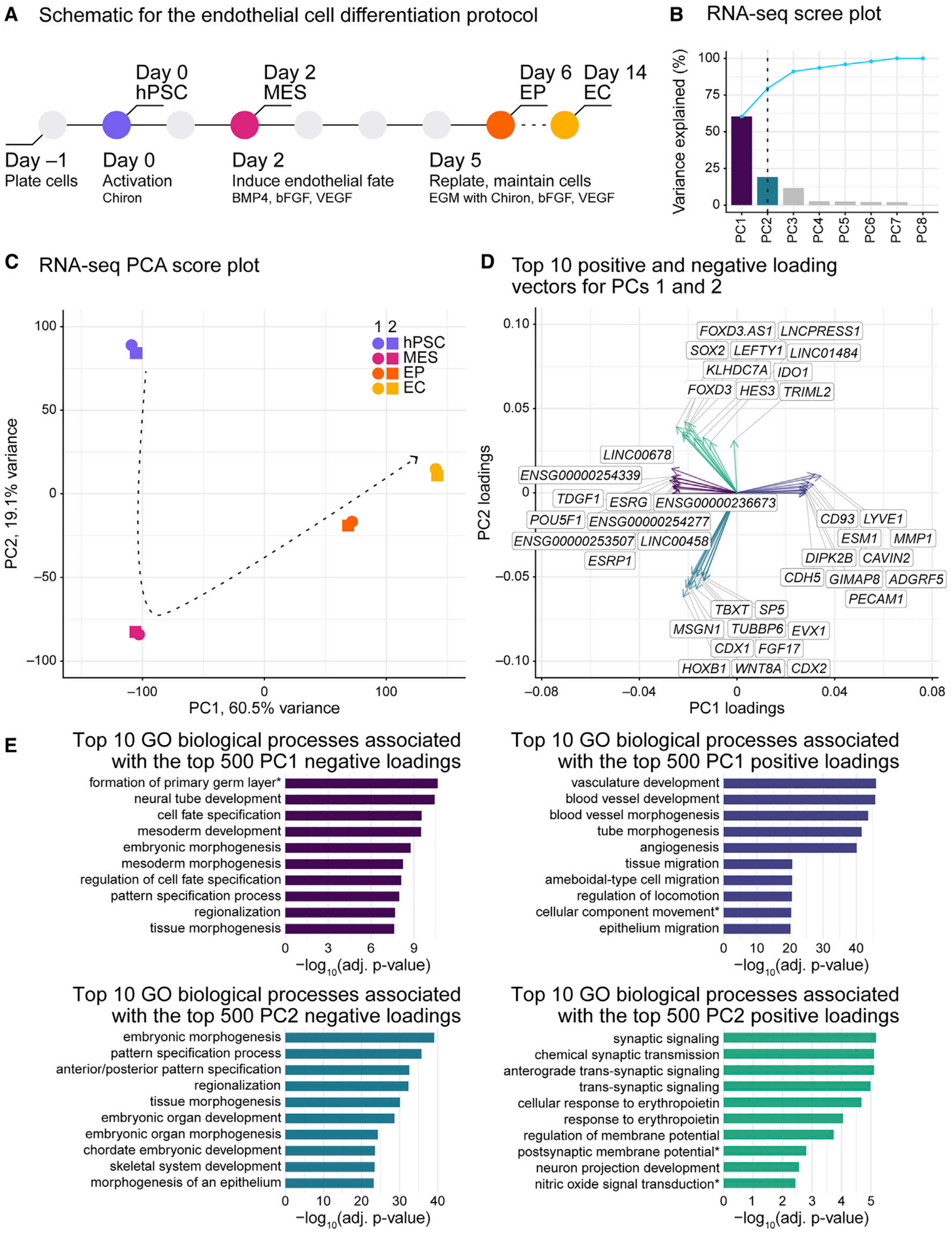

Figure 1. Transcription dynamics reveal epithelial-to-mesenchymal and mesenchymal-to-epithelial transitions in endothelial cell differentiation

(A) Schematic of the endothelial cell differentiation protocol. hPSC, human pluripotent stem cells; MES, mesoderm cells; EP, endothelial progenitor cells; EC, endothelial cells; BMP4, bone morphogenetic protein 4; bFGF, basic fibroblast growth factor; VEGF, vascular endothelial growth factor; EGM, endothelial cell growth medium.

(legend continued on next page)

RESULTS

Long-range *cis* contacts increase during endothelial cell differentiation

To study the dynamics and functional significance of 3D chromatin organization in endothelial cell development, we modified and performed a stepwise protocol to induce endocardial-like endothelial differentiation from hPSCs (Palpant et al., 2015, 2017). Using the RUES2 embryonic stem cell line (Figure S1A), we recapitulated key signaling events in endothelial cell development. First, we induced the mesodermal lineage through activation of the WNT signaling pathway; then, we directed the cells to an endothelial fate through the addition of bone morphogenetic protein 4 (BMP4), basic fibroblast growth factor (bFGF), and vascular endothelial growth factor (VEGF; Figure 1A). We took samples across a differentiation time course: day 0, pluripotent cells (hPSCs); day 2, mesodermal cells (MESs); day 6, endothelial progenitor cells (EPs); and day 14, endothelial cells (ECs; Figure 1A). We obtained high-purity cell populations as determined by flow cytometry with antibodies raised against two EC markers, CD34 and CD31: EPs were >75% pure and ECs were >90% pure (Figures S1B and S1C).

To assess the quality of the time course samples, we prepared and analyzed bulk RNA-seq datasets from two independent differentiations for each time point (Dataset S1). To evaluate relationships among the RNA-seq datasets, we performed principal component analysis (PCA). Horn's parallel analysis (Horn, 1965) revealed that, of the eight principal components (PCs), the first two are significant (Figure 1B); PC1 accounted for 60.5% of variance, and PC2 accounted for 19.1% (Figures 1B and 1C). A score plot for PCs 1 and 2 revealed the tight clustering of biological replicates and, in the separation of time points, an apparent EC developmental trajectory in which PC1 separated hPSCs and MESs from EPs and ECs, while PC2 separated mesenchymal cells (MESs) from epithelial cells (hPSCs, EPs, and ECs) (Figure 1C). Consistent with this, analysis of component loading vectors revealed the presence and enrichment of genes associated with endothelial development along the PC1 positive axis, formation of the embryonic primary germ layer on the PC1 negative axis, neural signaling and projection on the PC2 positive axis, and embryonic development and anterior/posterior pattern specification along the PC2 negative axis (Figures 1D and 1E and Dataset S2). Results from additional gene expression analyses were consistent with endothelial specification (Figures S1D–S1F, Dataset S2, and Note S1). Together with functional assays (Palpant et al., 2015, 2017) and the developmental trajectory uncovered by PCA, these results confirm cell identities and indicate that our differentiation protocol recapitulates the epithelial-to-mesenchymal and subsequent mesenchymal-to-epithelial transitions that occur in endothelial development.

To examine changes in chromatin organization over the course of EC differentiation, we prepared and analyzed *in situ* DNase Hi-C (Ramani et al., 2016) datasets from the same two independent differentiations for each time point. Various quality control checks revealed the data are of high quality (Figure S2, Dataset S1, and Note S2). Thus, for subsequent analyses of chromatin organization, we pooled replicates to increase the sequencing depth for each cell type.

Next, we surveyed features of chromatin organization across EC differentiation. At all time points, chromosome-wide contact maps displayed patterns indicative of local and longer-range chromatin interactions (Figures 2A and 2B). These included an abundance of "near" *cis* interactions in hPSCs (e.g., strong interactions along the diagonal of the hPSC panel) that spread outward with differentiation, increasing the numbers of "far" *cis* interactions. Next, we examined the *cis* chromatin contact probability $P(s)$ for pairs of genomic loci stratified by distance s (Lieberman-Aiden et al., 2009). Consistent with the *cis* contact maps, the proportion of long-range contacts increased over differentiation: hPSCs had the lowest proportion of long-range contacts >30 Mb; MESs and EPs had similar, higher,

(B) Bar chart showing proportions of variance explained for each principal component (PC) from principal component analysis (PCA) of RNA-seq data sampled from endothelial cell differentiation. Dashed black line, retained PCs—PCs 1 and 2—computed from Horn's parallel analysis (Horn, 1965); solid blue line, cumulative proportion of explained variance.

(C) PCA score plot for normalized RNA-seq data from endothelial cell differentiation with respect to PCs 1 and 2. Arrow, differentiation trajectory.

(D) PCA loading plot showing the top 10 positive and negative loading vectors (genes) for each axis (PCs 1 and 2).

(E) Bar charts depicting adjusted p values for the top 10 Gene Ontology (GO) biological process terms for the top 500 PC1 negative loading vectors (top left), the top 500 PC1 positive loading vectors (top right), the top 500 PC2 negative loading vectors (bottom left), and the top 500 PC2 positive loading vectors (bottom right). The p values are from hypergeometric tests with Bonferroni corrections. Terms with asterisks have been abbreviated: "formation of primary germ layer" is "cell fate commitment involved in formation of primary germ layer," "cellular component movement" is "regulation of cellular component movement," "postsynaptic membrane potential" is "regulation of postsynaptic membrane potential," and "nitric oxide signal transduction" is "nitric oxide-mediated signal transduction." See also Figure S1, Datasets S1 and S2, and Note S1.

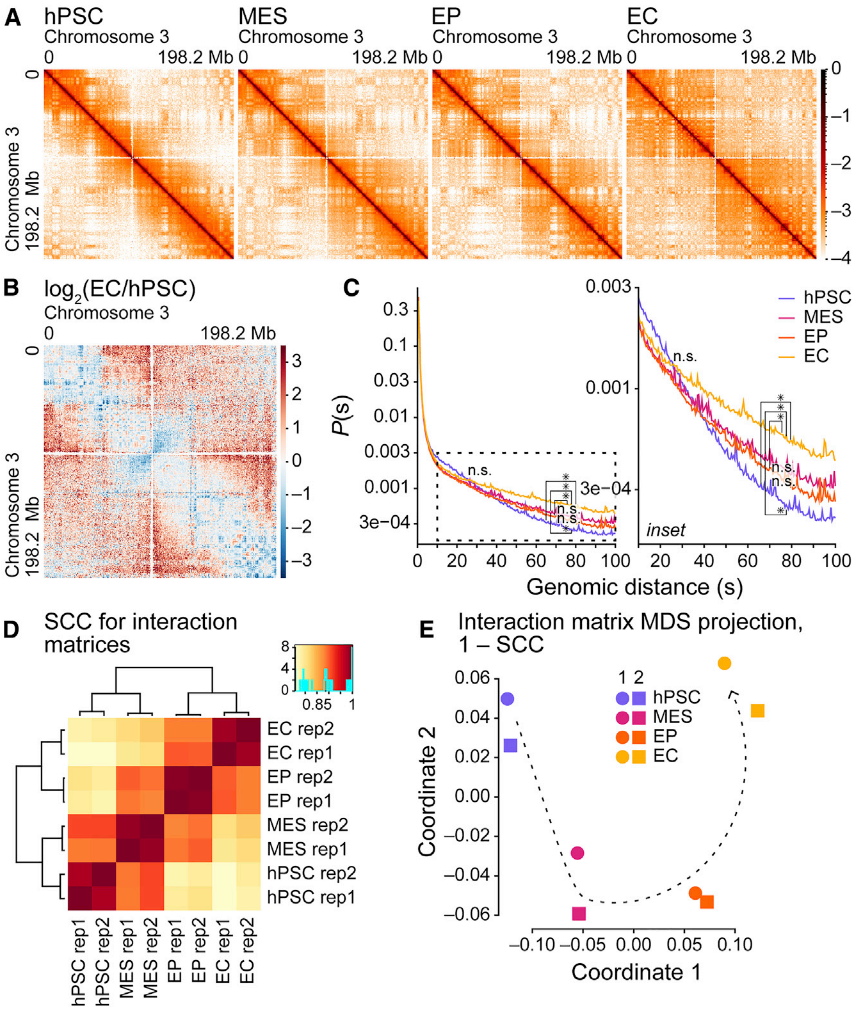

Figure 2. Long-range *cis* contacts increase during endothelial cell differentiation

(A) Heatmaps of normalized Hi-C interaction frequencies (500-kb resolution, chromosome 3) in hPSCs, MESs, EPs, and ECs.

(B) Log_2 ratios of normalized Hi-C interaction frequencies (500-kb resolution, chromosome 3) for ECs and hPSCs. Red, interactions increased in ECs; blue, interactions increased in hPSCs.

(C) Left: *cis* interaction frequency probabilities *P* stratified by distance *s* (Mb) over 0–100 Mb for Hi-C samples (500-kb resolution, autosomes). Inset, right: *P* stratified by *s* over 30–100 Mb. The p values at *s* of 30 and 75 Mb from pairwise t tests between samples (two independent replicates each) are shown; when adjusted with Benjamini-Hochberg *post hoc* tests, *p <0.05, n.s. (not significant). Adjusted p values at 30 Mb are n.s.

(D) Hierarchically clustered heatmap of HiCRep (Yang et al., 2017) stratum-adjusted correlation coefficients (SCCs) for Hi-C sample replicates (500-kb resolution, autosomes).

(E) Multidimensional scaling (MDS) projection of SCCs for Hi-C sample replicates (500-kb resolution); similarity measure: 1 − SCC. Arrow: differentiation trajectory. See also Figure S2, Dataset S1, and Note S2.

proportions of long-range contacts >30 Mb; and ECs exhibited the highest proportion of contacts >30 Mb (Figure 2C). These data reveal that long-range *cis* contacts increase over the course of EC differentiation.

To assess the influence of *cis* chromatin interactions as differentiation progress, we performed hierarchical clustering of stratum-adjusted correlation coefficients (SCCs) for *cis* interactions (Yang et al., 2017), clustering paired replicates while separating the datasets into two groups: an “early” group composed of hPSCs and MESs, and a “later” group composed of EPs and ECs (Figure 2D). Next, we performed multidimensional scaling (MDS) (Kruskal and Wish, 1977) of *cis* interaction maps using 1 − SCC as a measure of similarity. MDS paired replicates and arranged the samples in order of time point, revealing an apparent EC differentiation trajectory (Figure 2E) that resembles the epithelial-to-mesenchymal and mesenchymal-to-epithelial transitions captured by PCA of RNA-seq data (Figures 1B–1E). Together, these results indicate that changes in chromatin organization—including a gross increase in long-range *cis* chromatin contacts—are a key feature of EC differentiation, separating and ordering datasets by time point.

Dynamic compartmentalization reflects endothelial cell-specific changes in gene expression

Given these findings, we sought to understand how chromatin organization changes across differentiation and the functional significance of such changes. We investigated three forms of chromatin organization: genomic compartments, topologically associating domains, and peaks of elevated contact frequency referred to as “pairwise point interactions.” Pairwise point interactions are thought to represent DNA “loops”; however, we avoid using the term “loop” for its multiple meanings and interpretations as described in a recent review of chromosome organization (Mirny et al., 2019).

To begin, we focused on genomic compartments, the “plaid” patterns of chromatin interactions evident in Hi-C heatmaps (Lieberman-Aiden et al., 2009). Genomic

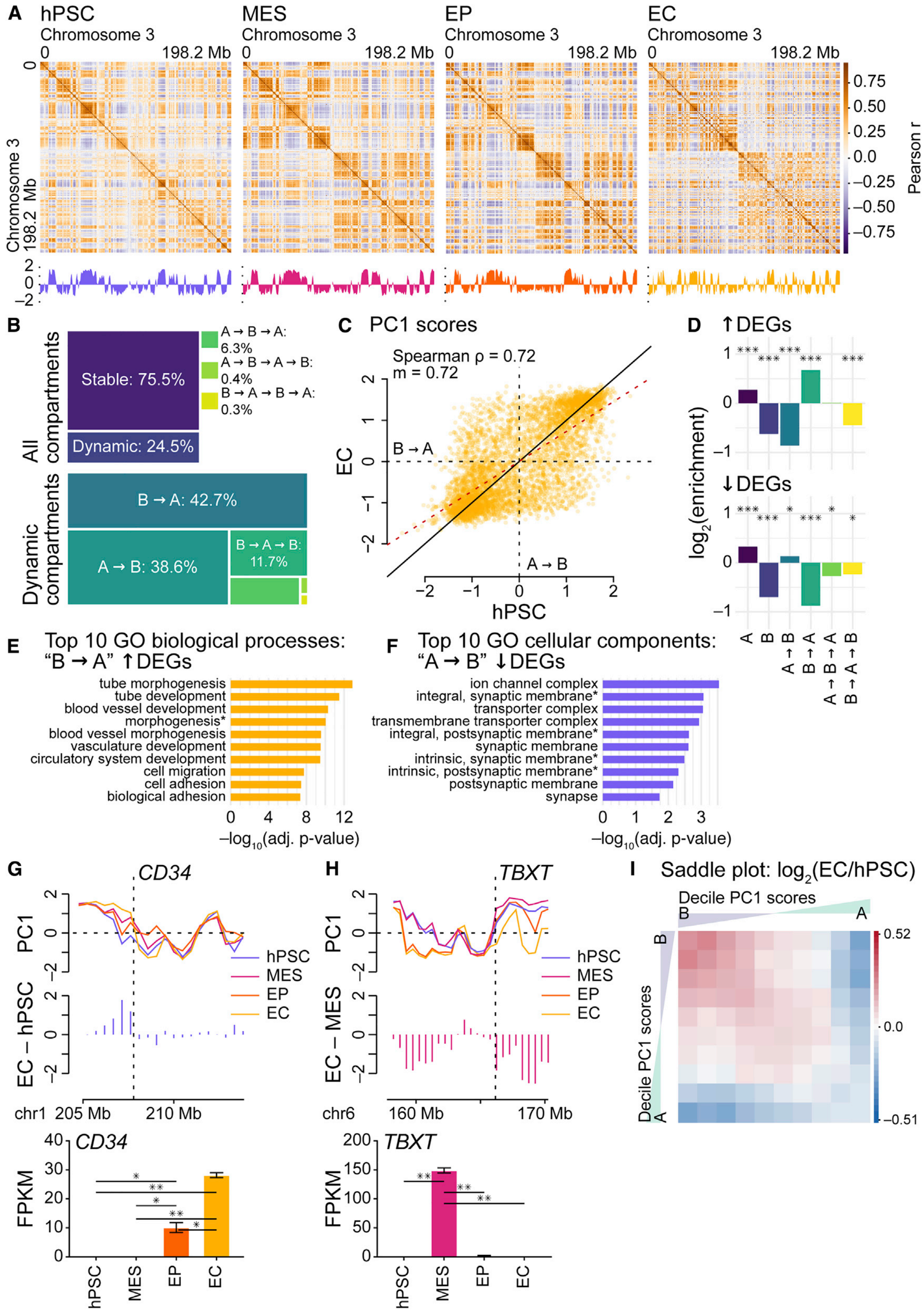

(legend on next page)

compartments represent at least two alternating states of chromatin, A and B, and each state preferentially interacts with loci in the same state. A compartments are associated with higher gene expression in euchromatin, while B compartments are associated with gene silencing in heterochromatin. To segregate genomic bins (500-kb resolution) into A/B compartments, we computed the PC1s of Pearson correlation-transformed contact matrices. We identified genomic compartments in all samples (Figure 3A). To assess changes in compartmentalization as ECs differentiate, we analyzed the proportions of stable and dynamic compartments. We found that approximately 25% of compartments are dynamic, undergoing one or more compartment switches across time points (Figures 3B, 3C, and S3A). Hierarchical clustering of Spearman correlation coefficients for PC1 scores paired and ordered replicates (Figure S3B); likewise, MDS paired replicates while revealing a differentiation trajectory similar to the other trajectories (Figures S3C, 1C, and 2E). Substantial numbers of compartment transitions take place in EC specialization, making compartment dynamism another key feature of EC differentiation.

In a study of differentiating cardiomyocytes (Bertero et al., 2019)—which share their origin in cardiogenic mesoderm with ECs (Palpant et al., 2015, 2017)—we found that compartment switches coincide with transcriptional regulation. To understand the influence of genomic compartmentalization on the dynamic transcriptomes of EC differentiation (Figures 1D, 1E, and S1D–S1F and Dataset S2), we calculated the enrichment of differentially expressed genes (DEGs; EC versus hPSC) with respect to stable and dynamic compartments (Figure 3D). Gene Ontology (GO) analyses (Ashburner et al., 2000; Chen et al., 2009; Gene Ontology Consortium, 2021) show that regions undergoing B-to-A transitions are enriched for DEGs upregulated in ECs, and these are associated with numerous functions in endothelial specification (Figure 3E and Dataset S2). Compartment changes are also associated with gene repression; e.g., in regions undergoing A-to-B transitions, DEGs downregulated in ECs are associated with neuronal development and function, genes that are suppressed in differentiation (Figure 3F and Dataset S2). Consistent with the enrichment of EC genes in B-to-A regions, the EC marker *CD34* is subject to a transition before elevated expression in EPs and ECs (Figure 3G). Similarly, the mesodermal marker *TBXT* is associated with an A-to-B transition, coincident with downregulation from MES to EP (Figure 3H). Thus, as with differentiating cardiomyocytes (Bertero et al., 2019), dynamic genomic compartmentalization is an important regulator of endothelial transcription.

Given that, during specification, long-range *cis* interactions increase (Figures 2A–2C) and 25% of the genome undergoes compartment transitions (Figures 3B, 3C, and S3A), we examined large-scale changes in genomic compartment strength across EC differentiation. We generated "saddle plots," which quantify the strength of compartment segregation, for hPSCs and ECs. We noted

Figure 3. Dynamic compartmentalization reflects endothelial cell-specific changes in gene expression
(A) Top: Pearson correlation coefficient matrices for normalized Hi-C interaction frequencies (500-kb resolution, chromosome 3) for endothelial cell samples. Bottom: principal component 1 (PC1) from principal component analysis.
(B) Top: tree map showing proportions of stable and dynamic genomic compartments in differentiation. Bottom: tree map showing, for dynamic genomic compartments, the proportions of types of compartment switches.
(C) Scatterplot comparing PC1 scores from hPSC and EC Hi-C samples (500-kb resolution, autosomes). ρ, Spearman correlation coefficient; m, regression slope; red dashed line, regression line; black solid line, x = y.
(D) Bar charts depicting $\log_2$ enrichment of up- (top) and downregulated (bottom) differentially expressed genes (DEGs) with respect to stable and dynamic genomic compartments (500-kb resolution, autosomes). Log_2 values, observed/gene density. DEGs were called via DESeq2 analysis (Love et al., 2014), EC versus hPSC (adjusted $p < 0.05$, absolute $\log_2$ fold change >1). Enrichment significantly different via chi-square tests with Yates corrections: *$p < 0.05$, ***$p < 0.001$.
(E and F) Bar charts depicting adjusted p values for the top 10 GO biological process or cellular component terms for genes located within bins (500-kb resolution, autosomes) that undergo (E) B-to-A and (F) A-to-B compartment transitions in endothelial specification. The p values are from hypergeometric tests with Bonferroni corrections. Terms with asterisks have been abbreviated: in (E), "morphogenesis" is "anatomical structure formation involved in morphogenesis," and in (F), "integral, synaptic membrane" is "integral component of synaptic membrane," "integral, postsynaptic membrane" is "integral component of postsynaptic membrane," "intrinsic, synaptic membrane" is "intrinsic component of synaptic membrane," and "intrinsic, postsynaptic membrane" is "intrinsic component of postsynaptic membrane."
(G and H) Top: line plots for PC1 scores at and within the vicinity of (G) *CD34* (chr1: 207.88–207.91 Mb) and (H) *TBXT* (chr6: 166.16–166.17 Mb) for Hi-C samples (500-kb resolution). Middle: bar charts indicating change in PC1 scores via the subtraction of (G) hPSC and (H) MES PC1 scores from EC PC1 scores. Bottom: bar charts for RNA-seq expression levels (FPKM) of (G) *CD34* and (H) *TBXT* in differentiation. The p values are from pairwise t tests between samples (two independent replicates each); when adjusted with Benjamini-Hochberg *post hoc* tests: *$p < 0.05$, **$p < 0.01$. Bar, mean; error bars, standard error of the mean.
(I) Log_2 ratio of "saddle plots," 10 × 10 decile-binned matrices quantifying the strength of ranked PC1 scores, for Hi-C samples (500-kb resolution, autosomes); red, interactions higher in ECs; blue, interactions higher in hPSCs. See also Figure S3 and Dataset S2.

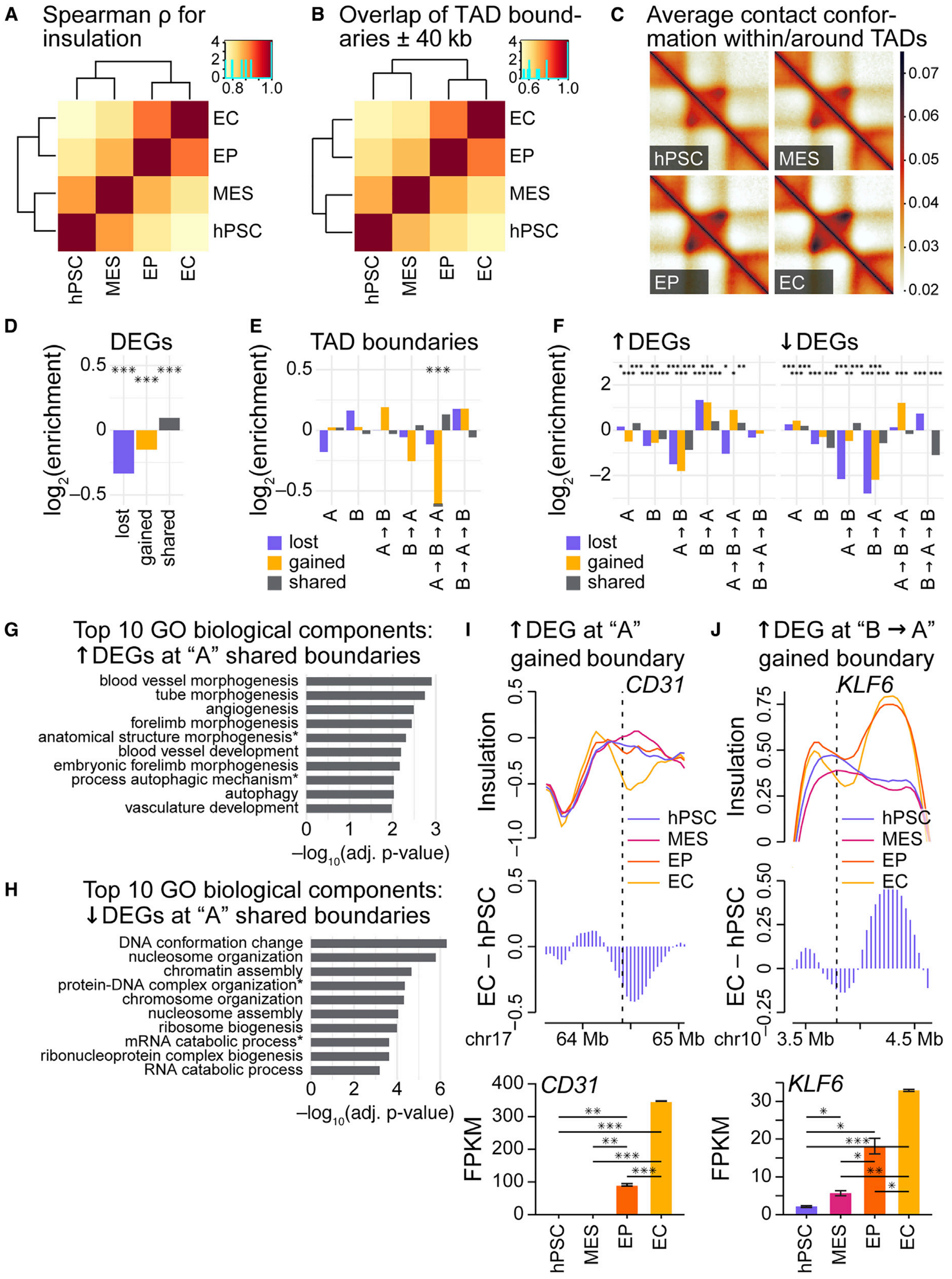

(legend on next page)

that stronger *cis* contacts occur between homotypic regions in comparison to heterotypic regions (Figures 3I and S3D); in differentiation, the strength of *cis* interactions between B compartments increased, while the strength between A compartments decreased. To evaluate the effects of strengthened B compartments, we compared gene expression distributions to background distributions. Observed expression distributions in stable and dynamic B compartment regions are significantly lower than background distributions (Figure S3E), indicating that—consistent with the differentiation of other tissues (Bertero et al., 2019; Bonev et al., 2017)—dynamic, strengthening B compartments repress transcription in endothelial specification.

Increasingly strengthened topologically associating domains regulate endothelial cell-specific gene expression

Next, we shifted focus to a different form of chromatin organization: topologically associating domains (TADs). TADs comprise local "neighborhoods" of increased chromatin contact frequency and are often delimited by insulator sequences (Dixon et al., 2012; Nora et al., 2012). Because TADs are thought to regulate gene expression (Dixon et al., 2012, 2015; Nora et al., 2012), we evaluated TAD features across EC differentiation. Using the insulation score approach to call TAD boundaries (Crane et al., 2015), we identified TADs in all samples taken across EC differentiation (Figure S4A).

Hierarchical clustering of Spearman correlations for TAD insulation scores gave results similar to those of hierarchical clustering of separate forms of chromatin organization (Figures 4A, 2D, S2A, and S3B). Replicates were paired, and the time-course datasets were separated into groups of early—hPSC, MES—and later samples—EP, EC—indicating an overall change in insulation scores across differentiation. We observed a similar dynamism for TAD boundaries: when measuring boundary intersections with a window of ±40 kb, 45.2% of TAD boundaries change position in differentiation (Figure 4B and Dataset S1); when the window size is increased to ±80 kb, 20.9% of TAD boundaries change (Figure S4B and Dataset S1). Clustering of intersections (windows of ±40 kb) revealed the same branching of early and later cell types (Figure 4B). Of note, EP and EC share the highest proportion of TAD boundaries (windows of ±40 kb): EP shares 78.1% of its boundaries with EC, EC shares 78.0% of its boundaries with EP. Next, we examined insulation-score changes at boundaries. Although scatterplots revealed limited changes in insulation scores at boundaries (Figure S4C), the differences in insulation to the immediate left and right of boundaries—i.e., "TAD boundary strengths" (Crane

Figure 4. Increasingly strengthened topologically associating domains regulate endothelial cell-specific gene expression
(A) Hierarchically clustered heatmap of Spearman correlation coefficients (ρ) for insulation scores from Hi-C samples (40-kb resolution, autosomes).
(B) Hierarchically clustered heatmap for TAD-boundary set intersections for Hi-C samples using a window of ±40 kb around boundaries (40-kb resolution, autosomes).
(C) Aggregate heatmaps representing average *cis* chromatin contact conformation around TADs for Hi-C samples (40-kb resolution, autosomes).
(D) Bar charts depicting $\log_2$ enrichment of differentially expressed genes (DEGs) proximal to (±80 kb) TAD boundaries (40-kb resolution, autosomes) lost in differentiation (hPSC-specific), gained in differentiation (EC-specific), and shared between time points (hPSC, EC). Log_2 values: observed/gene density. DEGs were called via DESeq2 analysis (Love et al., 2014), EC versus hPSC (adjusted $p < 0.05$, absolute $\log_2$ fold change >1). Enrichment significantly different via chi-square tests with Yates corrections: ***$p < 0.001$.
(E) Bar charts depicting $\log_2$ enrichment of lost, gained, and shared TAD boundaries in stable and dynamic compartments (500-kb resolution, autosomes). Log_2 values: observed/TAD union set. Enrichment significantly different via chi-square tests with Yates corrections: *$p < 0.05$, **$p < 0.01$, ***$p < 0.001$. Value for gained boundaries in A-to-B-to-A compartments: −1.2.
(F) Bar charts depicting $\log_2$ enrichment of up- (left) and downregulated (right) DEGs proximal to (±80 kb) boundaries grouped by stable and dynamic compartments. Log_2 values: observed/gene density. Enrichment significantly different via chi-square tests with Yates corrections: *$p < 0.05$, **$p < 0.01$, ***$p < 0.001$.
(G and H) Bar charts depicting adjusted p values for the top 10 Gene Ontology (GO) biological process terms for up- (G) and downregulated DEGs (H) proximal to (±80 kb) shared TAD boundaries in stable A compartments. The p values are from hypergeometric tests with Bonferroni corrections. Terms with asterisks have been abbreviated: in (G), "anatomical structure morphogenesis" is "anatomical structure formation involved in morphogenesis" and "process autophagic mechanism" is "process utilizing autophagic mechanism," and in (H), "protein-DNA complex organization" is "protein-DNA complex subunit organization" and "mRNA catabolic process" is "nuclear-transcribed mRNA catabolic process."
(I and J) Top: line plots for insulation scores at and within the vicinity of (I) *CD31* (chr17: 64.32–64.41 Mb) and (J) *KLF6* (chr10: 3.776–3.785) for Hi-C samples (40-kb resolution). Middle: bar charts indicating changes in insulation score via the subtraction of hPSC insulation scores from EC insulation scores. Bottom: bar charts for RNA-seq expression levels (FPKM) of (I) *CD31* and (J) *KLF6* in differentiation. The p values are from pairwise t tests between samples (two independent replicates each); when adjusted with Benjamini-Hochberg *post hoc* tests: *$p < 0.05$, **$p < 0.01$, ***$p < 0.001$. Bar, mean; error bars, standard error of the mean. See also Figure S4 and Datasets S1 and S2.

et al., 2015)—increased in differentiation (Figure S4D). Analyses of the average chromatin contact conformation within and around TADs reveal that, as cells differentiate, *cis* chromatin interactions are increasingly restricted within TAD boundaries (Figure 4C); this constraint is stronger in EC versus hPSC (Figure S4E). Although nearly 55% of all TAD boundaries are conserved in differentiation, dynamic boundaries converge on an EC state, and this is accompanied by a steady increase in the numbers of intra-TAD chromatin contacts.

We hypothesized that nascent boundaries and increased intra-TAD contacts regulate gene expression changes necessary for differentiation. Thus, we analyzed the enrichment of DEGs (EC versus hPSC) proximal to (within ±80 kb of) boundaries lost in differentiation (hPSC-specific boundaries), boundaries gained in differentiation (EC-specific), and shared boundaries (common to hPSC and EC). We found that DEGs are depleted from lost and gained boundaries and enriched at shared boundaries (Figure 4D). Relative to shared boundaries, gene expression is decreased at lost and gained boundaries (Figure S4F). These data raise the possibility that unshared boundaries are, on average, associated with repressive chromatin environments.

To investigate this, we examined the interplay of TADs and compartments. TADs in A compartments are smaller than those in B compartments (Figure S4G), a likely consequence of gene regulation in gene-dense, transcriptionally active regions. TAD boundaries lost in specification are slightly depleted from stable A compartments and dynamic regions of the genome that transition to A compartments, and slightly enriched in stable and dynamic B compartments (Figure 4E, purple). Shared boundaries are slightly enriched at dynamic A regions (Figure 4E, gray). TAD boundaries gained in specification are slightly enriched at dynamic B regions and depleted from dynamic A regions (Figure 4E, yellow). Consistent with this, the proportions of gained boundaries across compartment categories differ significantly from the proportions of shared boundaries (Figure S4H). These data support the assertion that TAD boundaries gained in differentiation tend to be associated with repressive chromatin.

This led us to explore how TAD-compartment interrelationships influence gene expression. We examined the enrichment of up- and downregulated DEGs at TAD boundaries grouped by stable and dynamic compartments (Figure 4F). Regardless of boundary type, upregulated DEGs are depleted from boundaries in A-to-B regions and enriched at boundaries in B-to-A regions (Figure 4F, left); downregulated DEGs are depleted from both A-to-B and B-to-A regions (Figure 4F, right). Similarly, gene expression is generally higher at boundaries in B-to-A regions and lower at boundaries in A-to-B regions (Figure S4I). These results indicate that, although TAD boundaries gained in differentiation tend to be associated with repressive chromatin environments, those boundaries that form in regions undergoing repressive-to-active chromatin transitions are enriched in upregulated DEGs. Thus, the relationship between TADs and gene expression is contextual, influenced by chromatin environment.

Our analyses also revealed that shared TAD boundaries in stable A regions of the genome are slightly but significantly enriched in both up- and downregulated DEGs (Figure 4F, gray). GO analyses of upregulated DEGs output terms associated with EC-specific functions (Figure 4G and Dataset S2); DEGs downregulated in ECs (and thus upregulated in hPSCs) are associated with chromatin organization and lability (Figure 4H and Dataset S2). Analyses of shared boundaries in stable A compartments indicate that, beyond gross dynamic compartment switching, additional gene-regulatory mechanisms are at play in endothelial specification.

Upregulated DEGs with essential roles in EC biology were observed at a subset of gained boundaries as well: the expression of *CD31* (Figure S1C) increases alongside boundary formation in a stable A compartment (Figure 4I), and the expression of *KLF6*, which encodes a Kruppel-like transcription factor that regulates genes involved in angiogenesis, vascular repair, and remodeling (Gallardo-Vara et al., 2016), increases with boundary formation in a B-to-A region (Figure 4J).

Long-range pairwise point interactions increase over differentiation and are associated with both gene activation and gene repression

Increasing evidence supports the importance of DNA pairwise point interactions (PPIs) in transcriptional regulation and suggests the existence of PPIs that function in specific aspects of development (Bonev et al., 2017; Gorkin et al., 2014). These interactions are thought to arise from the clustering of regulatory elements and genes through chromatin looping mechanisms (Rao et al., 2014). Using the point interaction-calling package HiCCUPs (Rao et al., 2014), we investigated PPI formation in EC differentiation. We identified increasing numbers of PPIs in differentiation: 623 in hPSCs to 3,881 in ECs (Figure 5A). Most loops are specific to one time point; few loops are shared by more than two time points. We observed the greatest number of stage-specific loops in ECs, suggesting a role for PPI-mediated transcriptional regulation in maturation. We examined the distances between PPI anchors across differentiation and observed a progressive expansion of PPI sizes (Figure 5B). Thus, endothelial specification sees PPIs increase in both frequency and distance.

Since PPI dynamics occur amid changes in genomic compartmentalization (Figures 3 and S3), and since TAD-compartment interplay is associated with essential

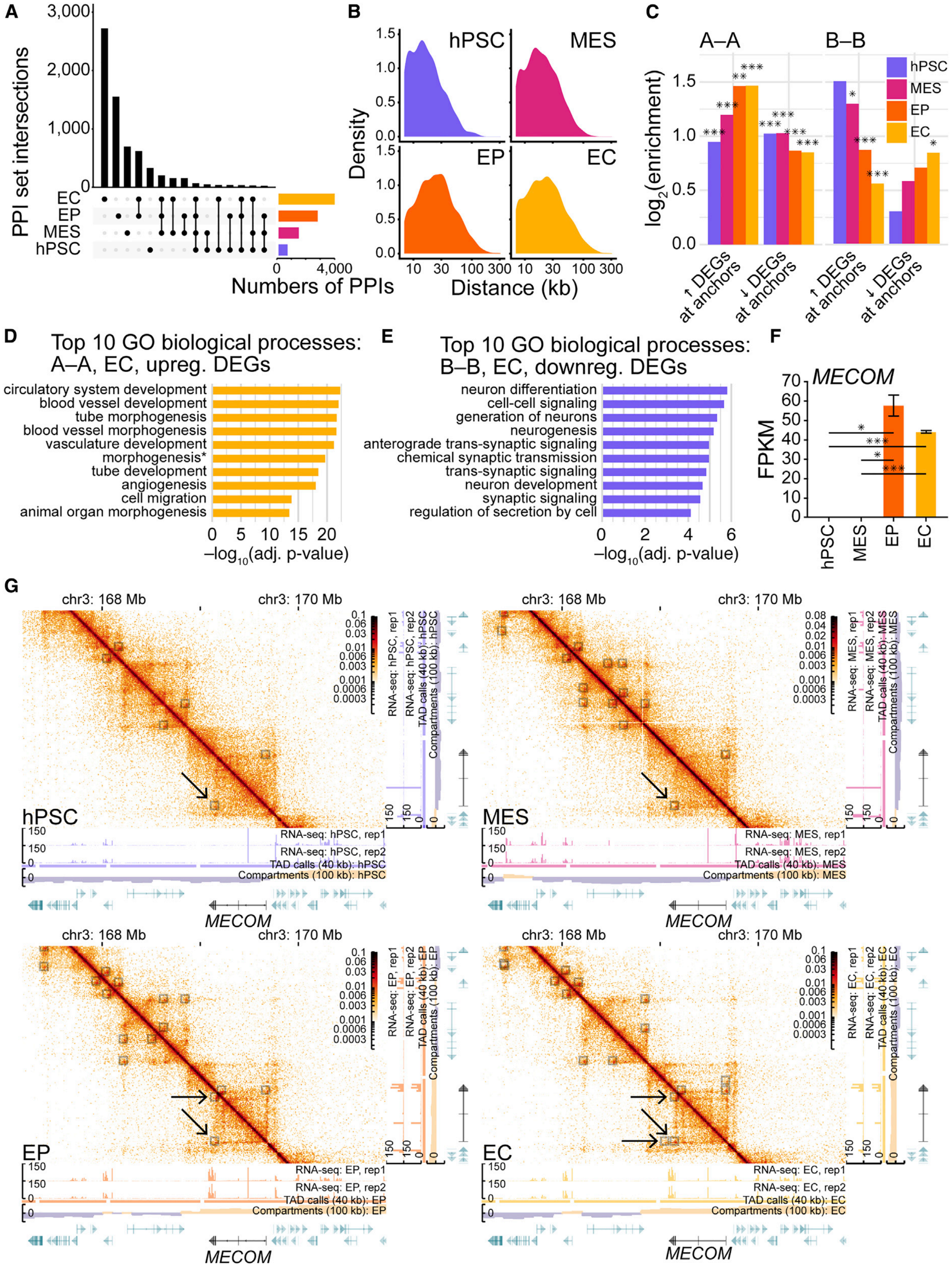

(legend on next page)

gene expression (Figures 4 and S4), we investigated PPI-compartment interrelationships. From MES to EC, the proportion of PPIs with both anchors in A compartments (A-A) increases, while the proportion with anchors in B compartments (B-B) decreases (Figures S5A and S5B). To investigate whether nascent A-A PPIs are involved in the transcriptional activation of genes essential to EC development, we evaluated the genome-wide enrichment of DEGs (EC versus hPSC) at time-point-specific anchors in A/B compartments. We observed an enrichment of DEGs at PPIs in all time points (Figure 5C and Dataset S1). The enrichment of PPIs at upregulated DEGs is elevated in comparison to downregulated DEGs. PPIs anchored in A compartments are enriched for upregulated DEGs and depleted for downregulated DEGs; the enriched upregulated DEGs are associated with numerous functions in endothelial specification (Figure 5D and Dataset S2). This trend was reversed for PPIs anchored in B compartments; the genes associated with B-compartment PPIs have numerous functions in neurogenesis (Figure 5E and Dataset S2), raising the possibility that B-compartment PPIs function in a transcriptional repression mechanism that inhibits neuronal specification. In a reciprocal analysis, we noted even stronger trends for the enrichment of PPI anchors at DEGs (Figure S5C), indicating that the overlap between DEGs and PPI anchors does not occur by chance. Our data suggest that PPIs facilitate both transcriptional activation within the A compartment and transcriptional repression within the B compartment.

Several genes with well-established roles in EC development associate with PPIs as their transcription increases. A prominent example is *MECOM*, which encodes a transcription factor that promotes arterial EC identity (McCracken et al., 2022). *MECOM* is expressed in EPs and ECs (Figure 5F), and comes to overlap multiple PPI anchors in differentiation (Figure 5G). Additional examples include *VEGFC* (Figure S5D), *KDR* (Figure S5E), and *TFPI* (Figure S5F and Note S3). Numerous genes repressed in EC differentiation are associated with B-compartment PPIs, including genes associated with tissue patterning and neuronal development; these include *EPHB6* (Figure S5G), *GABRB3/A5* (Figure S5H), and *PTPRZ1* (Figure S5I). We also observed examples of B-compartment PPI anchors associating with genes that code for factors with antiangiogenic properties, including *FOXC1* (Figure S5J) and *ISM1* (Figure S5K and Note S3).

Chromatin organization reveals the developmental divergence of endothelial cells and cardiomyocytes

Given their developmental origin from cardiogenic mesoderm (Palpant et al., 2015, 2017), we sought to compare genome organization in ECs versus cardiomyocytes, analyzing published time-course cardiomyocyte datasets (Bertero et al., 2019) with respect to our EC datasets. We took the $\log_2$ ratio of EC and cardiomyocyte (CM) contacts for a single chromosome, chromosome 3 (Figure 6A), noting elevated near-range interactions in ECs (red) and, in CMs, an increase in long-range interactions (blue). Beyond 30 Mb, there is a higher probability for *cis* contacts in CM versus EC (Figure 6B), indicating that, although long-range *cis* contacts increase as hPSCs differentiate to become ECs (Figures 2A–2C), longer-range *cis* contacts are present in—and a prominent feature of—genome organization in CM.

To examine the influence of *cis* chromatin interactions as ECs and CMs differentiate from their common origin, we performed hierarchical clustering of SCC scores, which

Figure 5. Long-range pairwise point interactions increase over differentiation and are associated with both gene activation and gene repression
(A) UpSet plot showing intersections of HiCCUPS (Rao et al., 2014) pairwise point interaction (PPI) anchors from Hi-C samples (10-kb resolution, autosomes). Vertical bars, PPI-anchor intersection sizes; horizontal bars, sample set sizes; black circles, anchors present; linked black circles, anchors shared between samples; gray circles, anchors absent.
(B) Density plots showing distributions of distances between PPI anchors from Hi-C samples (10-kb resolution, autosomes).
(C) Bar charts showing $\log_2$ enrichment of up- and downregulated differentially expressed genes (DEGs) at PPI anchors stratified by compartment type (A-A, A-B, and B-B; 100-kb resolution, autosomes) for Hi-C samples (10-kb resolution, autosomes). DEGs were called via DESeq2 analysis (Love et al., 2014), EC versus hPSC (adjusted $p < 0.05$, absolute $\log_2$ fold change >1). Enrichment significantly different via chi-square tests with Yates corrections: *$p < 0.05$, **$p < 0.01$, ***$p < 0.001$.
(D and E) Bar charts depicting adjusted p values for the top 10 Gene Ontology (GO) biological process terms for DEGs associated with (D) A-A and (E) B-B PPIs. The p values are from hypergeometric tests with Bonferroni corrections. Terms with asterisks have been abbreviated: in (D), "morphogenesis" is "anatomical structure formation involved in morphogenesis."
(F) Bar chart for *MECOM* RNA-seq expression levels (FPKM) in differentiation. The p values are from pairwise t tests between samples (two independent replicates each) adjusted with Benjamini-Hochberg *post hoc* tests: *$p < 0.05$, ***$p < 0.001$. Bar, mean; error bars, standard error of the mean.
(G) Visualization of PPIs associated with *MECOM* (chr3: 169.08–169.66 Mb). Heatmaps and tracks for normalized Hi-C interaction frequencies (10-kb resolution), RNA-seq signal (unadjusted), TADs (40-kb resolution), genomic compartments (100-kb resolution; gold, A compartment; purple, B compartment), and genes (green and black) are shown. Gray transparent squares over heatmaps, PPIs; arrows, PPIs associated with *MECOM*. See also Figure S5, Datasets S1 and S2, and Note S3.

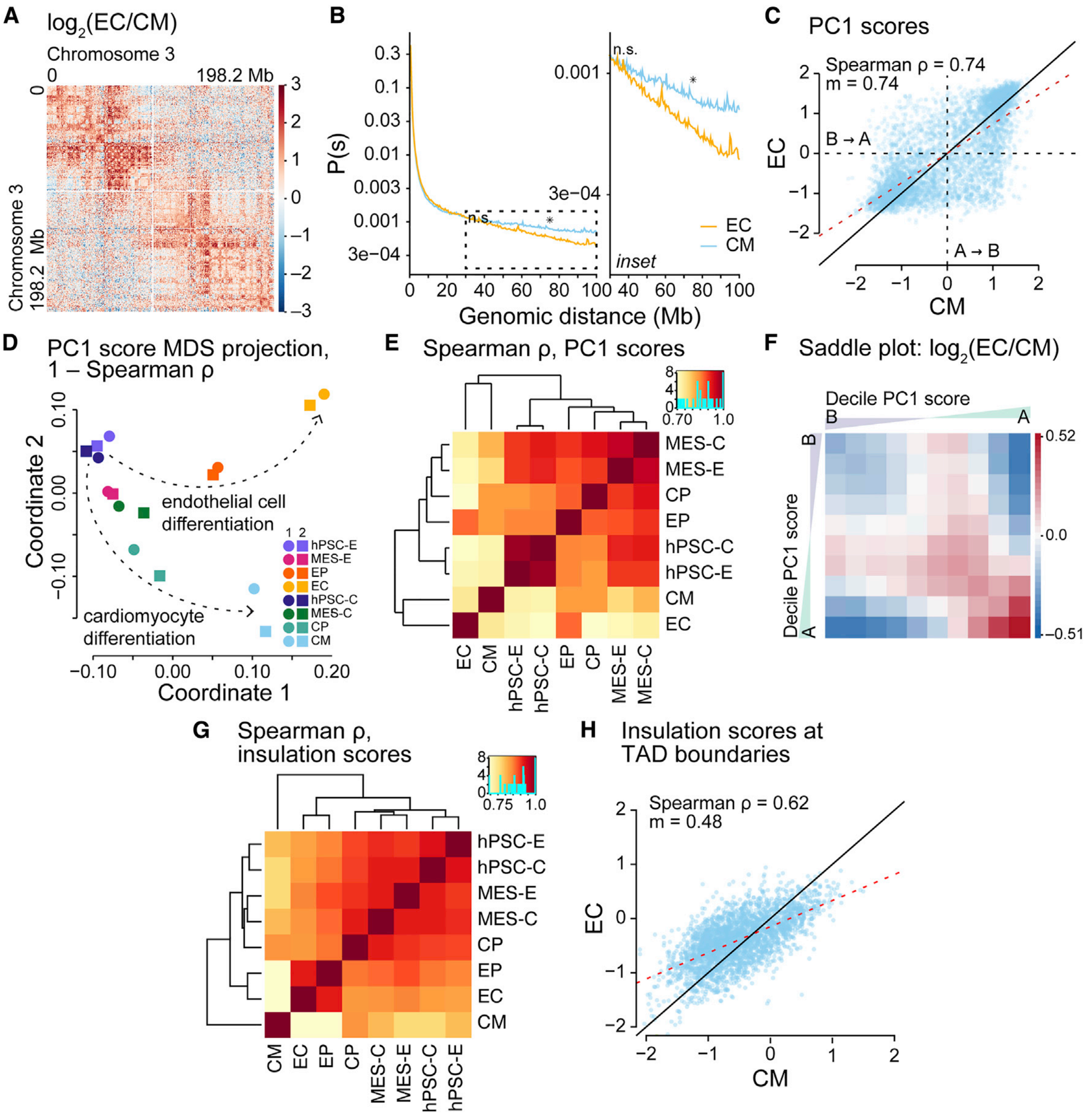

Figure 6. Chromatin organization reveals the developmental divergence of endothelial cells and cardiomyocytes

(A) Log_2 ratios of normalized Hi-C interaction frequencies (500-kb resolution, chromosome 3) for ECs versus cardiomyocytes (CMs). Red, interactions increased in ECs; blue, interactions increased in CMs.

(B) Left: *cis* interaction frequency probabilities p stratified by distance *s* (Mb) over 0–100 Mb for Hi-C samples (500-kb resolution, autosomes). Inset, right: p stratified by *s* over 30–100 Mb. The p-values at *s* of 30 and 75 Mb are from pairwise t tests between samples (two independent replicates each); when adjusted with Benjamini-Hochberg *post hoc* tests: $*p < 0.05$, n.s. (not significant).

(C) Scatterplot comparing PC1 scores from Hi-C samples (500-kb resolution, autosomes). ρ, Spearman correlation coefficient; m, regression slope; red dashed line, regression line; black solid line, x = y.

(D) MDS projection of PC1 scores for Hi-C samples (500-kb resolution, autosomes) taken from EC and CM differentiation. Similarity measure: $1 - \rho$. hPSC-E, human pluripotent stem cells from endothelial cell differentiation; MES-E, mesoderm cells from endothelial cell differentiation; hPSC-C, human pluripotent stem cells from cardiomyocyte differentiation; MES-C, mesoderm cells from cardiomyocyte differentiation; CP, cardiomyocyte progenitor cells.

(E) Hierarchically clustered heatmap of Spearman correlation coefficients (ρ) for PC1 scores from Hi-C samples (500-kb resolution, autosomes).

(F) Log_2 ratios of saddle plots for Hi-C samples (500-kb resolution, autosomes); red, interactions higher in EC; blue, interactions higher in CM.

(legend continued on next page)

apportioned samples into distinct groups related to time point and cell type (Figure S6A and Dataset S1). hPSC and MES cell types clustered into separate groups, and CM progenitor cells (CPs) were observed amid the MES samples. Increasingly differentiated cell types—EP, EC, and CM—occupied their own groups. Thus, *cis*-contact dynamism tightly correlates with the developmental divergence of the two cell types.

Given that, we investigated the influence of genomic compartments on EC and CM differentiation. Scatterplots for PC1 scores in EC versus CM revealed marked changes in compartment status in EC versus CM (Figures 6C and S6B). MDS of PC1 scores grouped replicates and outlined divergent differentiation trajectories (Figure 6D). Hierarchical clustering of Spearman correlation coefficients for PC1 scores paired replicates (Figure 6E) and revealed that the most differentiated samples, EC and CM, have the most distinct compartment profiles. We calculated and plotted the $\log_2$ ratio of EC and CM saddle plots, finding that *cis* interactions in B compartments are generally weaker in EC versus CM, while *cis* interactions between A compartments are generally stronger (Figures 6F and S6C). Taken together, these data reveal genomic compartmentalization as an important distinguishing feature of the two lineages. Considering their varying transcriptomes (Bertero et al., 2019), dynamic compartmentalization likely influences the two transcription programs.

Next, we asked whether TAD dynamics varied between EC and CM. Hierarchical clustering of TAD insulation scores grouped early cell states—hPSC, MES, and also CP—and separated out later cell states—EP, EC, and CM (Figure 6G). Of note, insulation scores in CMs were markedly different from all other cell types. Hierarchical clustering of TAD boundary set intersections (windows of ±40 and ±80 kb) apportioned samples similar to clustered insulation scores (Figure S6D and Dataset S1), separating early states from later states. We homed in on TAD dynamics across EC and CM by drawing scatterplots for insulation scores at boundaries, revealing strong changes in insulation scores in EC versus CM (Figures 6H and S6E) and a trend in which CM insulation scores tend to be higher when EC insulation scores are lower. These results suggest that the two lineages develop distinct chromatin topologies: one in which intra-TAD chromatin interactions are tightly bounded in ECs, serving to hinder longer-range chromatin interactions; on the other hand, CM TAD boundaries are less restrictive, and thus CM interactions are able to form longer *cis* chromatin interactions.

DISCUSSION

In this study, we advanced a model system in which hPSCs are differentiated to ECs, and we used this system to present a comprehensive look at chromatin organization on its own and with respect to the dynamic transcriptomes of differentiation (Note S4).

Our examination of genomic compartmentalization in EC differentiation revealed that ~25% of the genome is dynamic, transitioning from transcriptionally active, euchromatic A compartments to repressive, heterochromatic B compartments or vice versa. Regions that transition from B to A are enriched in upregulated DEGs related to endothelial specification; regions that transition from A to B are enriched in downregulated DEGs related to neurogenesis. We also observed the increasing strength of B compartments in differentiation, suggesting the compaction of chromatin (Bertero et al., 2019). Given the large changes in transcription that occur in EC differentiation, it is possible that dynamic, strengthening B compartments function to repress transcription through chromatin inaccessibility mechanisms. These findings are in line with those reported in recent Hi-C analyses of CMs and other models for differentiation (Bertero et al., 2019; Paulsen et al., 2019) and, taken together, indicate that dynamic genomic compartmentalization is an important, conserved regulator of cell-type-specific transcription.

In addition to genomic compartmentalization, we assessed TADs and PPIs, two other forms of chromatin organization. TADs and PPIs are similar in that both arise through a process of chromatin loop extrusion (Fudenberg et al., 2016; Nora et al., 2017; Sanborn et al., 2015). In loop extrusion, the multisubunit protein complex cohesin entraps small loops of chromatin inside its lumen; through progressive extrusion, the loops are enlarged, ceasing to grow when cohesin colocalizes with the transcription factor/insulator protein CTCF, the CTCF cofactor MAZ (Ortabozkoyun et al., 2022), and likely other “architectural proteins” (Rowley et al., 2016). It has been suggested that cohesin is subject to rapid turnover (Haarhuis et al., 2017; Nora et al., 2017), limiting the time span for cohesin-chromatin interactions and, thus, loop extrusion. Cohesins can also extend beyond CTCF-bound consensus sequences: under conditions where cohesin turnover is prolonged or stopped, cohesin appears to move beyond CTCF (Schwarzer et al., 2017; Tedeschi et al., 2013). We observed that most PPIs are unique to each stage of EC differentiation, with each successive stage in our time-point analysis

(G) Hierarchically clustered heatmap of Spearman correlation coefficients for insulation scores from Hi-C samples (40-kb resolution, autosomes).
(H) Scatterplot comparing insulation scores from Hi-C samples (40-kb resolution, autosomes). ρ, Spearman correlation coefficients; m, regression slope; black dashed line, regression line; black solid line, x = y. See also Figure S6 and Dataset S1.

seeing more and more PPIs. The EC stage exhibits the most PPIs in addition to the strongest TAD boundaries and highest number of intra-TAD contacts. Considering these findings together, we speculate that cohesin loading is increased and turnover is prolonged, allowing for strengthened TAD boundaries and increased numbers of PPIs in maturation. These findings are consistent with observations in a prior study (Niskanen et al., 2018), which reported high levels of TAD connectivity, identifying EC-specific long-range interactions between TADs enriched for histone H3 trimethylated at lysine 9 (H3K9me3), a marker of constitutive heterochromatin. While it remains to understand how the strengthened TADs and numerous PPIs arise in ECs, our findings indicate that these features are influenced by stable and dynamic compartments to regulate transcription necessary for differentiation (Note S4).

We also performed comparative analyses of dynamic chromatin organization in endothelial specification versus CM specification, observing that, although B compartments strengthen in EC differentiation, B compartments do not strengthen to the extent seen in CM differentiation (Bertero et al., 2019). Alongside this, insulation scores are generally lower at TAD boundaries in EC versus CM, indicating that *cis* chromatin interactions are less restrained in EC versus CM. These observations indicate that high levels of heterochromatinization and long-range chromatin contacts are prevalent features of genome organization in CM versus EC. The CM nuclear environment is notable for a *trans*-interaction network of *TTN*-associated genes facilitated by the muscle-specific splicing protein RBM20 (Bertero et al., 2019). Could it be that CM differentiation, with its preponderance of long-range interactions, sees the establishment of a nuclear environment that facilitates functionally significant regulatory *trans* interactions, while EC development sees the development of a nuclear environment that bounds long-range contacts and comes to support regulation through predominantly *cis* forms of chromatin organization, e.g., PPIs?

Attempting to address this and other questions will undoubtedly fuel further research. With this study, we provide a comprehensive analysis of 3D chromatin organization in a model of EC development that will be a key resource for studying EC biology in health and disease.

EXPERIMENTAL PROCEDURES

Details are provided in the supplemental experimental procedures.

Resource availability

Corresponding author

Further information and requests should be directed to and will be fulfilled by co-lead contact Charles E. Murry (murry@uw.edu).

Materials availability

This study did not generate new unique reagents.

Data and code availability

Data have been deposited in the 4DN data portal and are publicly available at https://data.4dnucleome.org/Alavattam-Mitzelfelt-endothelial-differentiation-chromatin; previously published CM differentiation data are available from GEO: GSE106690 (Bertero et al., 2019). Source code for analyses performed in this study is available at github.com/Noble-Lab/2020_kga0_endothelial-diff.

Statistics

No statistical methods were used to predetermine sample sizes. No data were excluded from analyses. The experiments were not randomized and investigators were not blinded to allocation during experiments and assessment. Where appropriate, the mean is reported as a measurement of central tendency, and the SEM is used as a measure of precision. Statistical significance was thresholded at $\alpha = 0.05$; $p < \alpha$ are considered significant. Sample and replicate numbers are reported in figure and supplemental figure captions where appropriate. Statistical analyses were performed using base R (version 4.1) or various software packages (supplemental experimental procedures). Strategies for stratification, sampling, and enrichment are described in the supplemental experimental procedures, as are statistical tests used in this study.

SUPPLEMENTAL INFORMATION

Supplemental information can be found online at https://doi.org/10.1016/j.stemcr.2022.11.003.

AUTHOR CONTRIBUTIONS

K.G.A., K.A.M., W.S.N., and C.E.M. wrote the manuscript with edits provided by all other authors. K.G.A., K.A.M., G.B., W.S.N., and C.E.M. designed the experiments. K.A.M. conducted wet-lab experiments; K.G.A., G.B., P.A.F., and X.Y. conducted dry-lab experiments. K.G.A., K.A.M., X.Y., L.P., A.B., N.J.P., H.S.C., W.S.N., and C.E.M. interpreted the results. W.S.N. and C.E.M. obtained resources and were the overall supervisors of this work.

ACKNOWLEDGMENTS

We thank Dr. Choli Lee and the lab of Dr. Jay Shendure (Department of Genome Sciences, University of Washington) for assistance with sequencing, Drs. Nicole Zeinstra and Ying Zheng (Department of Bioengineering, University of Washington) for expertise and advice regarding endothelial cells, and the University of Washington Center for Nuclear Organization and Function Group for feedback and discussion. We gratefully acknowledge the Tom and Sue Ellison Stem Cell Core of the Institute for Stem Cell and Regenerative Medicine for use of cell culture space and equipment. This research was supported by the Cell Analysis Facility Flow Cytometry and Imaging Core in the Department of Immunology at the University of Washington. This work was funded in part by NIH awards UM1HG011531 (W.S.N.), U54 DK107979 (W.S.N. and C.E.M.), R01 HL146868 (C.E.M.), and HL148081 (C.E.M.), as well as a grant from the Fondation Leducq Transatlantic Network of Excellence (C.E.M.).

CONFLICT OF INTERESTS

C.E.M. is an employee of and equity holder in Sana Biotechnology.

Received: August 27, 2022
Revised: November 7, 2022
Accepted: November 7, 2022
Published: December 8, 2022

REFERENCES

Ashburner, M., Ball, C.A., Blake, J.A., Botstein, D., Butler, H., Cherry, J.M., Davis, A.P., Dolinski, K., Dwight, S.S., Eppig, J.T., et al.; The Gene Ontology Consortium (2000). Gene ontology: tool for the unification of biology. Nat. Genet. *25*, 25–29.

Bertero, A., Fields, P.A., Ramani, V., Bonora, G., Yardimci, G.G., Reinecke, H., Pabon, L., Noble, W.S., Shendure, J., and Murry, C.E. (2019). Dynamics of genome reorganization during human cardiogenesis reveal an RBM20-dependent splicing factory. Nat. Commun. *10*, 1538.

Bonev, B., Mendelson Cohen, N., Szabo, Q., Fritsch, L., Papadopoulos, G.L., Lubling, Y., Xu, X., Lv, X., Hugnot, J.-P., Tanay, A., et al. (2017). Multiscale 3D genome rewiring during mouse neural development. Cell *171*, 557–572.e24.

Chen, J., Bardes, E.E., Aronow, B.J., and Jegga, A.G. (2009). ToppGene Suite for gene list enrichment analysis and candidate gene prioritization. Nucleic Acids Res. *37*, W305–W311. https://doi.org/10.1093/nar/gkp427.

Crane, E., Bian, Q., McCord, R.P., Lajoie, B.R., Wheeler, B.S., Ralston, E.J., Uzawa, S., Dekker, J., and Meyer, B.J. (2015). Condensin-driven remodelling of X chromosome topology during dosage compensation. Nature *523*, 240–244.

De Val, S., and Black, B.L. (2009). Transcriptional control of endothelial cell development. Dev. Cell *16*, 180–195.

Dixon, J.R., Selvaraj, S., Yue, F., Kim, A., Li, Y., Shen, Y., Hu, M., Liu, J.S., and Ren, B. (2012). Topological domains in mammalian genomes identified by analysis of chromatin interactions. Nature *485*, 376–380.

Dixon, J.R., Jung, I., Selvaraj, S., Shen, Y., Antosiewicz-Bourget, J.E., Lee, A.Y., Ye, Z., Kim, A., Rajagopal, N., Xie, W., et al. (2015). Chromatin architecture reorganization during stem cell differentiation. Nature *518*, 331–336.

Fudenberg, G., Imakaev, M., Lu, C., Goloborodko, A., Abdennur, N., and Mirny, L.A. (2016). formation of chromosomal domains by loop extrusion. Cell Rep. *15*, 2038–2049.

Gallardo-Vara, E., Blanco, F.J., Roqué, M., Friedman, S.L., Suzuki, T., Botella, L.M., and Bernabeu, C. (2016). Transcription factor KLF6 upregulates expression of metalloprotease MMP14 and subsequent release of soluble endoglin during vascular injury. Angiogenesis *19*, 155–171.

Gene Ontology Consortium (2021). The Gene Ontology resource: enriching a GOld mine. Nucleic Acids Res. *49*, D325–D334.

Gorkin, D.U., Leung, D., and Ren, B. (2014). The 3D genome in transcriptional regulation and pluripotency. Cell Stem Cell *14*, 762–775.

Haarhuis, J.H.I., van der Weide, R.H., Blomen, V.A., Yáñez-Cuna, J.O., Amendola, M., van Ruiten, M.S., Krijger, P.H.L., Teunissen, H., Medema, R.H., van Steensel, B., et al. (2017). The cohesin release factor WAPL restricts chromatin loop extension. Cell *169*, 693–707.e14. https://doi.org/10.1016/j.cell.2017.04.013.

Horn, J.L. (1965). A rationale and test for the number of factors in factor analysis. Psychometrika *30*, 179–185.

Kruskal, J.B., and Wish, M. (1977). Multidimensional Scaling (Beverly Hills, CA: Sage Publications).

Lieberman-Aiden, E., van Berkum, N.L., Williams, L., Imakaev, M., Ragoczy, T., Telling, A., Amit, I., Lajoie, B.R., Sabo, P.J., Dorschner, M.O., et al. (2009). Comprehensive mapping of long-range interactions reveals folding principles of the human genome. Science *326*, 289–293.

Love, M.I., Huber, W., and Anders, S. (2014). Moderated estimation of fold change and dispersion for RNA-seq data with DESeq2. Genome Biol. *15*, 550.

McCracken, I.R., Dobie, R., Bennett, M., Passi, R., Beqqali, A., Henderson, N.C., Mountford, J.C., Riley, P.R., Ponting, C.P., Smart, N., et al. (2022). Mapping the developing human cardiac endothelium at single cell resolution identifies MECOM as a regulator of arteriovenous gene expression. Cardiovasc. Res., cvac023. https://doi.org/10.1093/cvr/cvac023.

Mirny, L.A., Imakaev, M., and Abdennur, N. (2019). Two major mechanisms of chromosome organization. Curr. Opin. Cell Biol. *58*, 142–152.

Niskanen, H., Tuszynska, I., Zaborowski, R., Heinäniemi, M., Ylä-Herttuala, S., Wilczynski, B., and Kaikkonen, M.U. (2018). Endothelial cell differentiation is encompassed by changes in long range interactions between inactive chromatin regions. Nucleic Acids Res. *46*, 1724–1740.

Nora, E.P., Lajoie, B.R., Schulz, E.G., Giorgetti, L., Okamoto, I., Servant, N., Piolot, T., van Berkum, N.L., Meisig, J., Sedat, J., et al. (2012). Spatial partitioning of the regulatory landscape of the X-inactivation centre. Nature *485*, 381–385.

Nora, E.P., Goloborodko, A., Valton, A.-L., Gibcus, J.H., Uebersohn, A., Abdennur, N., Dekker, J., Mirny, L.A., and Bruneau, B.G. (2017). Targeted degradation of CTCF decouples local insulation of chromosome domains from genomic compartmentalization. Cell *169*, 930–944.e22. https://doi.org/10.1016/j.cell.2017.05.004.

Ortabozkoyun, H., Huang, P.-Y., Cho, H., Narendra, V., LeRoy, G., Gonzalez-Buendia, E., Skok, J.A., Tsirigos, A., Mazzoni, E.O., and Reinberg, D. (2022). CRISPR and biochemical screens identify MAZ as a cofactor in CTCF-mediated insulation at Hox clusters. Nat. Genet. *54*, 202–212.

Palpant, N.J., Pabon, L., Roberts, M., Hadland, B., Jones, D., Jones, C., Moon, R.T., Ruzzo, W.L., Bernstein, I., Zheng, Y., et al. (2015). Inhibition of β-catenin signaling respecifies anterior-like endothelium into beating human cardiomyocytes. Development *142*, 3198–3209.

Palpant, N.J., Pabon, L., Friedman, C.E., Roberts, M., Hadland, B., Zaunbrecher, R.J., Bernstein, I., Zheng, Y., and Murry, C.E. (2017). Generating high-purity cardiac and endothelial derivatives from patterned mesoderm using human pluripotent stem cells. Nat. Protoc. *12*, 15–31.

Paulsen, J., Liyakat Ali, T.M., Nekrasov, M., Delbarre, E., Baudement, M.-O., Kurscheid, S., Tremethick, D., and Collas, P. (2019). Long-range interactions between topologically associating domains shape the four-dimensional genome during differentiation. Nat. Genet. *51*, 835–843.

Rajendran, P., Rengarajan, T., Thangavel, J., Nishigaki, Y., Sakthisekaran, D., Sethi, G., and Nishigaki, I. (2013). The vascular endothelium and human diseases. Int. J. Biol. Sci. *9*, 1057–1069.

Ramani, V., Cusanovich, D.A., Hause, R.J., Ma, W., Qiu, R., Deng, X., Blau, C.A., Disteche, C.M., Noble, W.S., Shendure, J., et al. (2016). Mapping 3D genome architecture through in situ DNase Hi-C. Nat. Protoc. *11*, 2104–2121.

Rao, S.S.P., Huntley, M.H., Durand, N.C., Stamenova, E.K., Bochkov, I.D., Robinson, J.T., Sanborn, A.L., Machol, I., Omer, A.D., Lander, E.S., et al. (2014). A 3D map of the human genome at kilobase resolution reveals principles of chromatin looping. Cell *159*, 1665–1680. https://doi.org/10.1016/j.cell.2014.11.021.

Rowley, M.J., Corces, V.G., and Corces, V.G. (2016). The three-dimensional genome: principles and roles of long-distance interactions. Curr. Opin. Cell Biol. *40*, 8–14. https://doi.org/10.1016/j.ceb.2016.01.009.

Sanborn, A.L., Rao, S.S.P., Huang, S.-C., Durand, N.C., Huntley, M.H., Jewett, A.I., Bochkov, I.D., Chinnappan, D., Cutkosky, A., Li, J., et al. (2015). Chromatin extrusion explains key features of loop and domain formation in wild-type and engineered genomes. Proc. Natl. Acad. Sci. USA *112*, E6456–E6465. https://doi.org/10.1073/pnas.1518552112.

Schwarzer, W., Abdennur, N., Goloborodko, A., Pekowska, A., Fudenberg, G., Loe-Mie, Y., Fonseca, N.A., Huber, W., Haering, C.H., Mirny, L., et al. (2017). Two independent modes of chromatin organization revealed by cohesin removal. Nature *551*, 51–56. https://doi.org/10.1038/nature24281.

Tedeschi, A., Wutz, G., Huet, S., Jaritz, M., Wuensche, A., Schirghuber, E., Davidson, I.F., Tang, W., Cisneros, D.A., Bhaskara, V., et al. (2013). Wapl is an essential regulator of chromatin structure and chromosome segregation. Nature *501*, 564–568. https://doi.org/10.1038/nature12471.

Xu, Y. (2014). Transcriptional regulation of endothelial dysfunction in atherosclerosis: an epigenetic perspective. J. Biomed. Res. *28*, 47–52.

Yang, T., Zhang, F., Yardımcı, G.G., Song, F., Hardison, R.C., Noble, W.S., Yue, F., and Li, Q. (2017). HiCRep: assessing the reproducibility of Hi-C data using a stratum-adjusted correlation coefficient. Genome Res. *27*, 1939–1949.

Stem Cell Reports, Volume *18*

Supplemental Information

Dynamic chromatin organization and regulatory interactions in human endothelial cell differentiation

Kris G. Alavattam, Katie A. Mitzelfelt, Giancarlo Bonora, Paul A. Fields, Xiulan Yang, Han Sheng Chiu, Lil Pabon, Alessandro Bertero, Nathan J. Palpant, William S. Noble, and Charles E. Murry

Supplemental Information

Supplemental figures

Figure S1. Validation of stem cell line and the purity of endothelial cells, and transcriptomes are highly dynamic in endothelial cell specification, related to Figure 1

Figure S2. Insulation-score consistency indicates Hi-C data are of high quality, related to Figure 2

Figure S3. Dynamic compartmentalization reveals an endothelial cell differentiation trajectory that resembles the transcriptome-identified trajectory, related to Figure 3

Figure S4. In differentiation, TAD boundaries converge on an endothelial cell state, and gained boundaries tend to be associated with repressive chromatin environments, related to Figure 4

Figure S5. PPI anchors are enriched at sites of DEGs, and examples of PPIs associated with gene repression, related to Figure 5

Figure S6. Chromatin topologies differ in endothelial cells versus cardiomyocytes, related to Figure 6

Supplemental datasets

Dataset S1. RNA-seq and Hi-C dataset metrics, stratum-adjusted correlation coefficients, proportions of overlapping TAD boundaries, and numbers of DEGs associated with PPI anchors, related to Figures 1, 2, 4–6

Dataset S2. GO terms for PC loadings, analyses of differentially expressed genes (DEGs), DEGs associated with B-to-A and A-to-B compartment transitions, DEGs associated with shared TAD boundaries in stable A compartments, and DEGs associated with PPI anchors in A and B compartments, related to Figures 1, 3–5

Supplemental notes

Note S1. Transcriptomes undergo overt, cell type-relevant changes in endothelial cell differentiation, related to Figure 1

Note S2. Hi-C datasets are of high quality as indicated by read alignment metrics, analyses of insulation-score consistency, and evaluation of stratum-adjusted correlation coefficients, related to Figure 2

Note S3. In differentiation, endothelial cell genes associate with PPIs in both eu- and heterochromatic regions, related to Figure 5

Note S4. Additional comments and interpretation, related to Discussion

Supplemental experimental procedures

Cell culture

Flow cytometry

Hi-C: Sample preparation, library generation, and sequencing

RNA-seq: Sample preparation, library generation, and sequencing

Hi-C: Data-sourcing, alignment, processing, and quality control

Hi-C: Generation and visualization of Hi-C heatmaps

Hi-C: *Cis* contact-decay curve analyses

Hi-C: Genomic compartment analyses

Hi-C: Multidimensional scaling

Hi-C: Analyses of topologically associating domains

Hi-C: Enrichment of topologically associating domain boundaries with respect to genomic compartments

Hi-C: Analyses of pairwise point interactions

RNA-seq: Sourcing, alignment, and gene-level quantification of alignments

RNA-seq: Principal component analysis

RNA-seq: Gene expression analysis

RNA-seq: Differential gene expression analysis

RNA-seq: Gene Ontology analyses

RNA-seq: Enrichment of differentially expressed genes with respect to genomic compartments

RNA-seq: Enrichment of differentially expressed genes with respect to topologically associating domain boundaries

RNA-seq: Enrichment of differentially expressed genes with respect to pairwise point interactions

Statistics

Figure preparation

Supplemental references

Supplemental figures

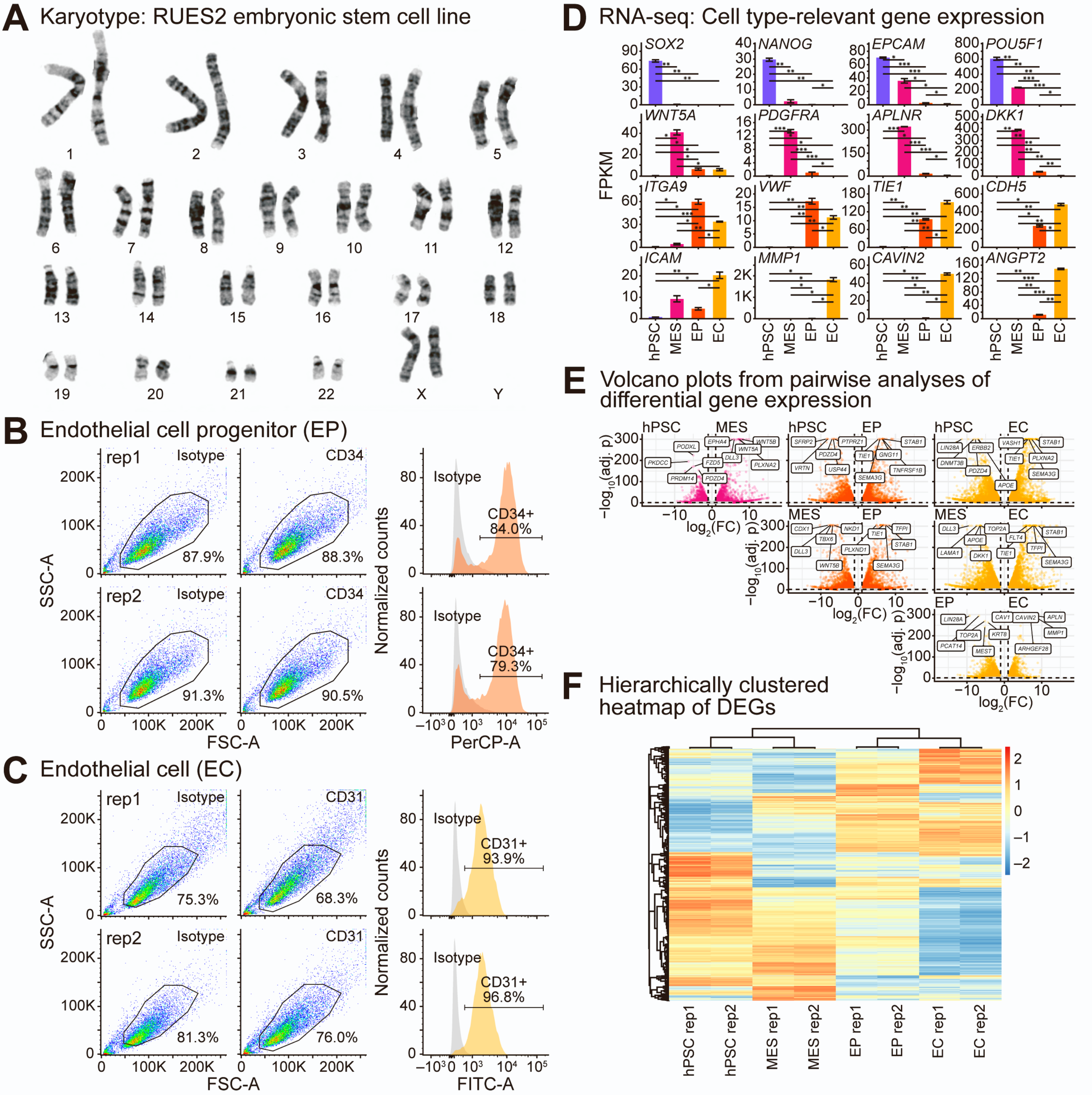
A Karyotype: RUES2 embryonic stem cell line
1
2
3
4
5
6
7
8
9
10
11
12
13
14
15
16
17
18
19
20
21
22
X
Y
B Endothelial cell progenitor (EP)
rep1
rep2
Isotype
CD34
87.9%
88.3%
91.3%
90.5%
CD34+ 84.0%
CD34+ 79.3%
SSC-A
FSC-A
Normalized counts
PerCP-A
C Endothelial cell (EC)
rep1
rep2
Isotype
CD31
75.3%
68.3%
81.3%
76.0%
CD31+ 93.9%
CD31+ 96.8%
SSC-A
FSC-A
Normalized counts
FITC-A
D RNA-seq: Cell type-relevant gene expression
FPKM
SOX2
NANOG
EPCAM
POU5F1
WNT5A
PDGFRA
APLNR
DKK1
ITGA9
VWF
TIE1
CDH5
ICAM
MMP1
CAVIN2
ANGPT2
hPSC
MES
EP
EC
E Volcano plots from pairwise analyses of differential gene expression
hPSC
MES
EP
EC
$-\log_{10}$(adj. p)
$\log_2$(FC)
F Hierarchically clustered heatmap of DEGs
hPSC rep1
hPSC rep2
MES rep1
MES rep2
EP rep1
EP rep2
EC rep1
EC rep2

Figure S1. Validation of stem cell line and the purity of endothelial cells, and transcriptomes are highly dynamic in endothelial cell specification, related to Figure 1

A. Karyotype analysis of RUES2 human embryonic stem cells demonstrating a normal 46,XX pattern.

B. Left: flow cytometry scatter plot for endothelial progenitor cells (EP) via immunostaining against human anti-IgG antibody, a negative control. Middle: flow cytometry scatter plot indicating EP purity via immunostaining against mouse anti-human CD34-PerCP antibody. Right: overlapping histograms for EP stained against the IgG isotype (gray) and CD34 (orange). Counts were normalized to modes. Top: replicate 1; bottom: replicate 2.

C. Left: flow cytometry scatter plot for endothelial cells (EC) as in panel **A**. Middle: flow cytometry scatter plot indicating EC purity via immunostaining against mouse anti-human CD31-FITC antibody. Right: overlapping histograms of EC stained against the IgG isotype (gray) and CD31 (yellow). Counts were normalized to modes. Top: replicate 1; bottom: replicate 2.

D. Bar charts for RNA-seq expression levels (FPKM) for genes relevant to time point-specific cell functions in endothelial cell differentiation. hPSC: human pluripotent stem cells; MES: mesoderm cells; EP: endothelial progenitor cells; EC: endothelial cells. P-values from pairwise t-tests between samples (two independent replicates each) adjusted with Benjamini-Hochberg post-hoc tests: * < 0.05, ** < 0.01, *** < 0.001. Bar: mean; error bars: standard error of the mean (SEM).

E. Volcano plots showing magnitude of change ($\log_2$ fold change, or “FC”) versus significance ($-\log_{10}$ adjusted p-values) for differentially expressed genes (DEGs) from pairwise DESeq2 analyses (Love et al., 2014) (adjusted p-value < 0.05, absolute $\log_2$ fold change > 1) of RNA-seq samples from endothelial cell differentiation: human pluripotent stem cells (hPSC) versus mesoderm cells (MES), endothelial progenitor cells (EP), and endothelial cells (EC; top row); MES versus EP and EC (middle row); and EP versus EC (bottom row). Labels: the top five down- and upregulated DEGs per analysis.

F. Hierarchically clustered heatmap of DEGs from pairwise analyses as in panel **A**. Color scale: $\log_2$-transformed, z score-normalized expression (FPKM).

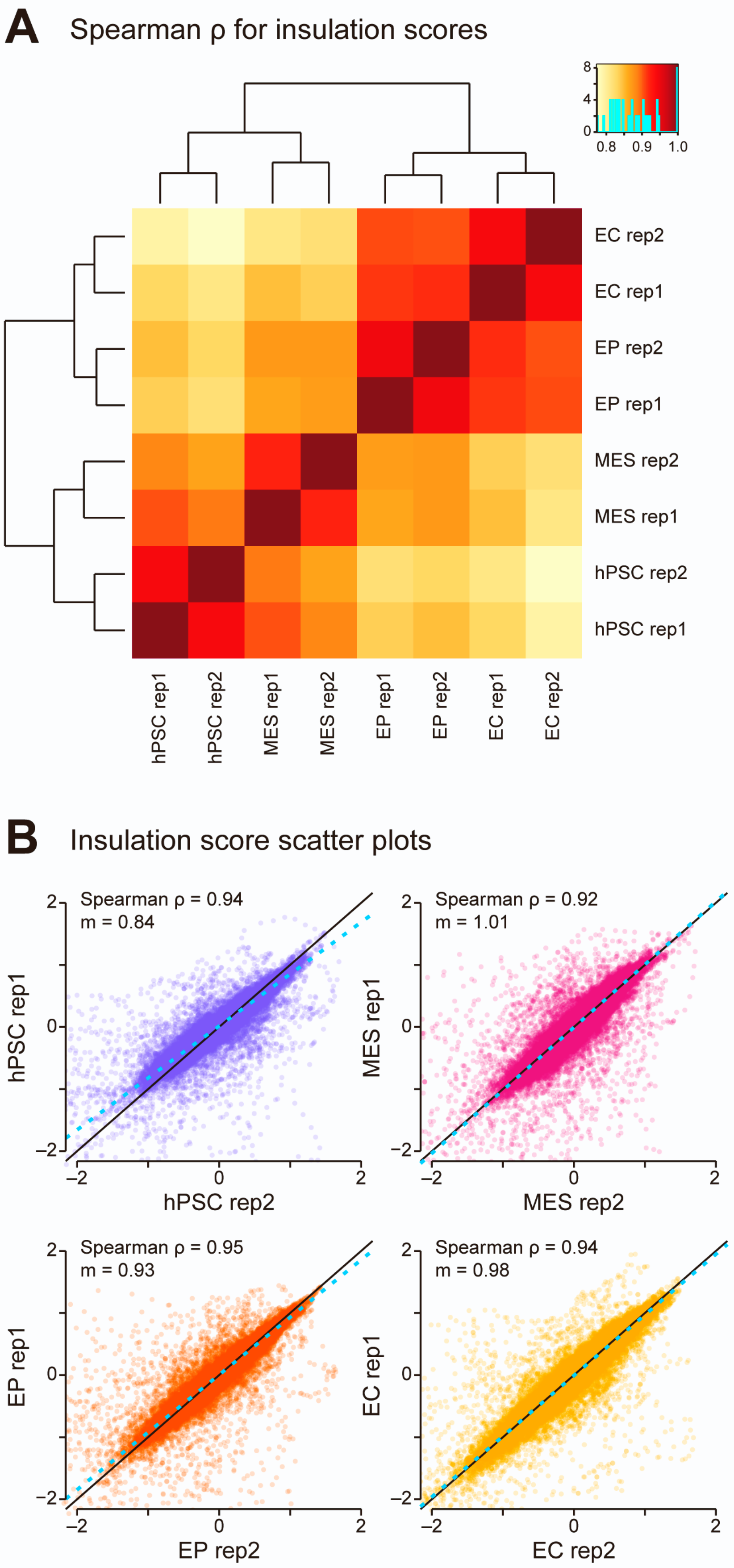

Alavattam, Mitzelfelt et al. – Figure S2

Figure S2. Insulation-score consistency indicates Hi-C data are of high quality, related to Figure 2

A. Hierarchically clustered heatmap of Spearman correlation coefficients (ρ) for insulation scores from Hi-C sample replicates (40-kb resolution, autosomes) taken across endothelial cell differentiation.

B. Scatter plots for Hi-C sample replicate (40-kb resolution, autosomes) insulation scores: hPSC (top left), MES (top right), EP (bottom left), and EC (bottom right). ρ: Spearman correlation coefficients; m: regression slope; blue dashed line: regression line; black solid line: x = y.

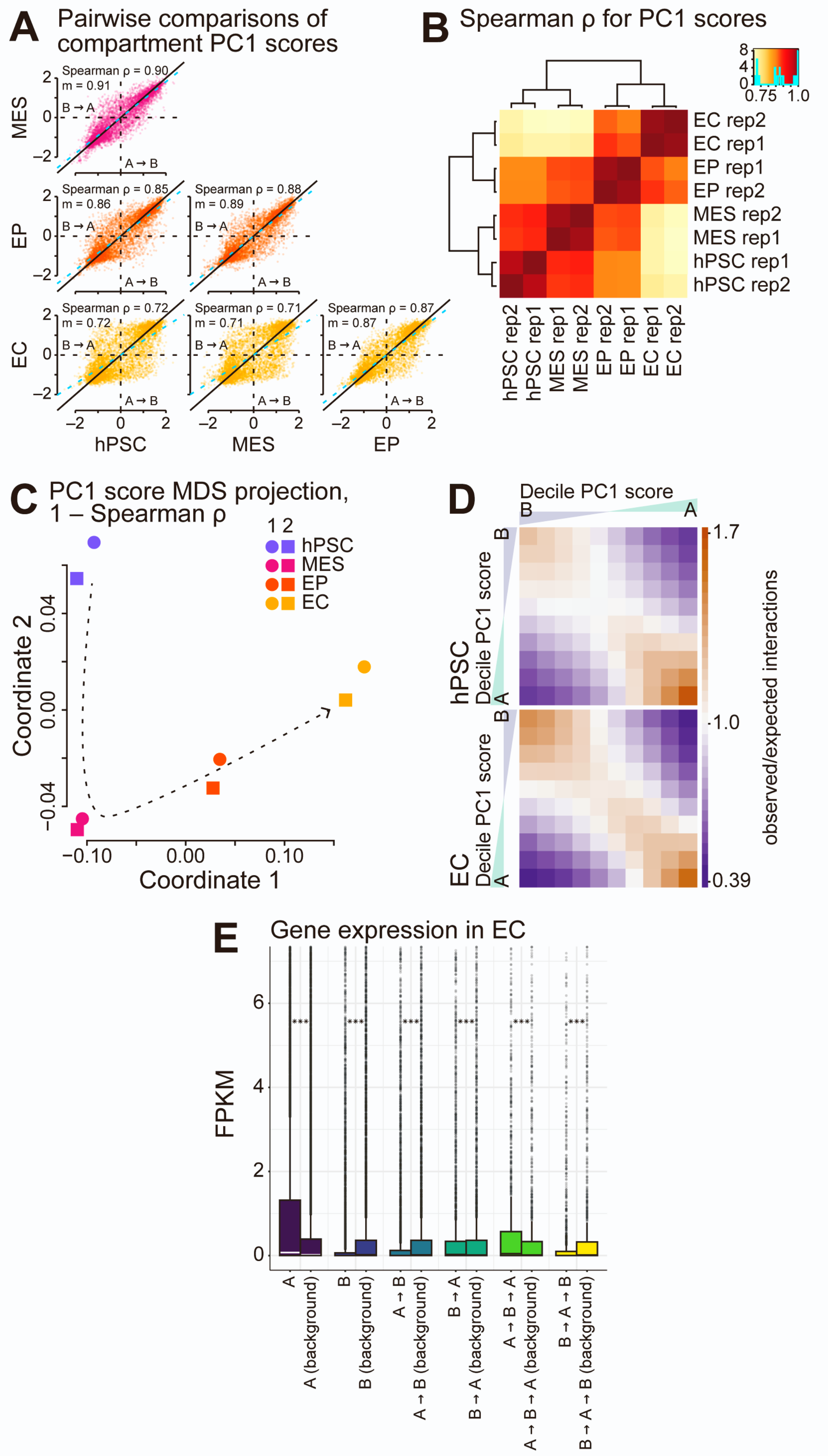
A
Pairwise comparisons of compartment PC1 scores
Spearman ρ = 0.90
m = 0.91
Spearman ρ = 0.85
m = 0.86
Spearman ρ = 0.88
m = 0.89
Spearman ρ = 0.72
m = 0.72
Spearman ρ = 0.71
m = 0.71
Spearman ρ = 0.87
m = 0.87
B → A
A → B
MES
EP
EC
hPSC
MES
EP
B
Spearman ρ for PC1 scores
EC rep2
EC rep1
EP rep1
EP rep2
MES rep2
MES rep1
hPSC rep1
hPSC rep2
C
PC1 score MDS projection, 1 – Spearman ρ
1 2
hPSC
MES
EP
EC
Coordinate 2
Coordinate 1
D
Decile PC1 score
hPSC Decile PC1 score
EC Decile PC1 score
observed/expected interactions
1.7
1.0
0.39
E
Gene expression in EC
FPKM
A
A (background)
B
B (background)
A → B
A → B (background)
B → A
B → A (background)
A → B → A
A → B → A (background)
B → A → B
B → A → B (background)

Figure S3. Dynamic compartmentalization reveals an endothelial cell differentiation trajectory that resembles the transcriptome-identified trajectory, related to Figure 3

A. Scatter plots for Hi-C sample (500-kb resolution, autosomes) PC1 scores: hPSC versus MES (top row), hPSC versus EP, MES versus EP (middle row); hPSC versus EC, MES versus EC, EP versus EC (bottom row). ρ: Spearman correlation coefficient; m: regression slope; blue dashed line: regression line; black solid line: x = y.

B. Hierarchically clustered heatmap of Spearman correlation coefficients (ρ) for PC1 scores from Hi-C samples (500-kb resolution, autosomes).

C. MDS projection of PC1 scores for Hi-C samples (500-kb resolution, autosomes); similarity measure: 1 – Spearman correlation coefficient (ρ). Arrow: differentiation trajectory.

D. Saddle plots for EC and hPSC Hi-C samples (500-kb resolution, autosomes). Gold-to-purple color bar: observed/expected interactions.

E. Box-and-whisker plots showing EC gene expression (FPKM; averaged from two independent replicates) in stable (A, B) and dynamic (A-to-B, B-to-A, A-to-B-to-A, B-to-A-to-B) compartments. Box-and-whisker plots represent the 25th percentile, median, and 75th percentile; whiskers extend to 1.5 times the interquartile range. P-values from pairwise Kolmogorov-Smirnov tests between observed and corresponding background distributions adjusted with Benjamini-Hochberg post-hoc tests: *** < 0.001.

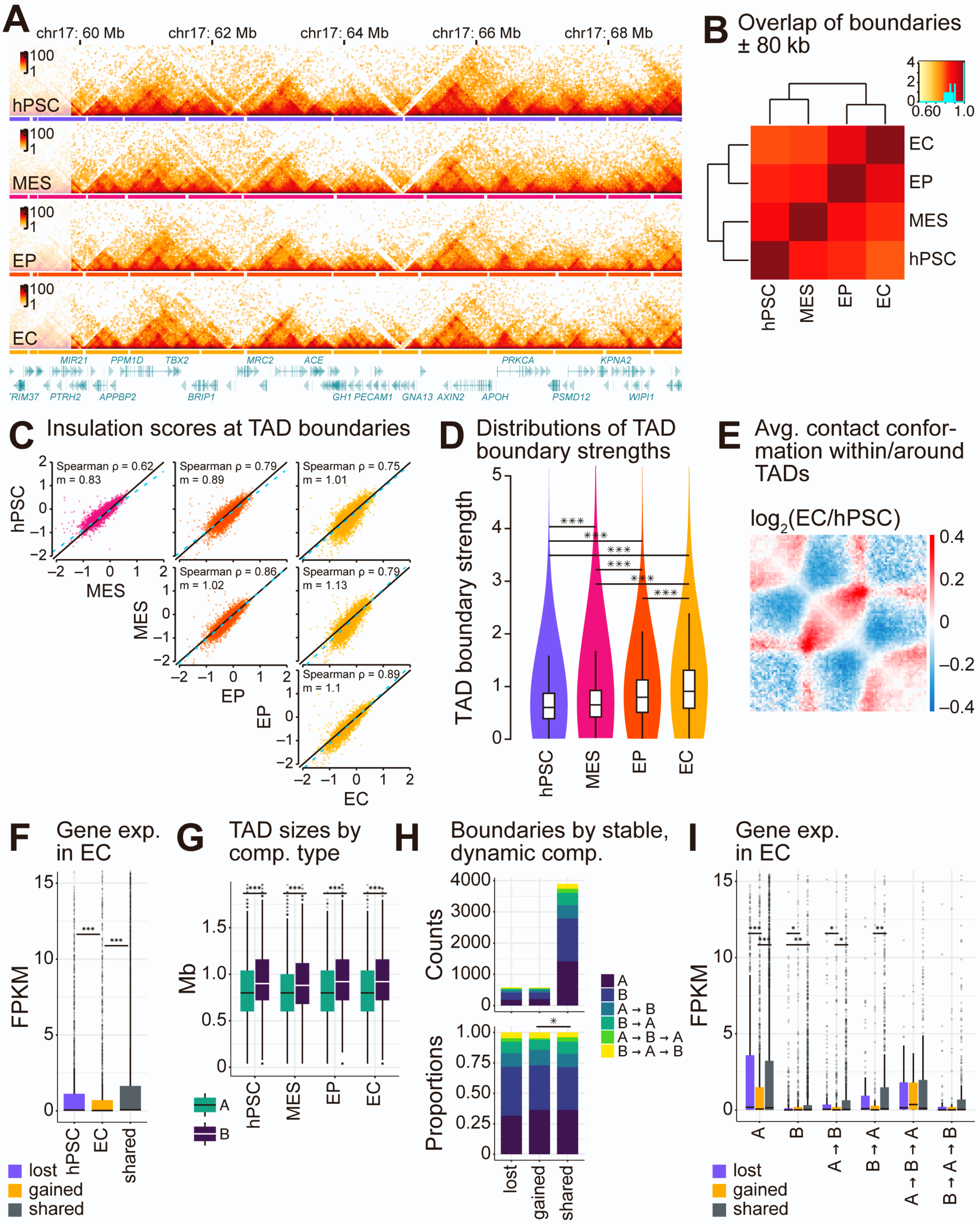
A
chr17: 60 Mb
chr17: 62 Mb
chr17: 64 Mb
chr17: 66 Mb
chr17: 68 Mb
hPSC
MES
EP
EC
MIR21
PPM1D
TBX2
MRC2
ACE
PRKCA
KPNA2
PTRH2
APPBP2
BRIP1
GH1 PECAM1
GNA13 AXIN2
APOH
PSMD12
WIPI1
B
Overlap of boundaries ± 80 kb
C
Insulation scores at TAD boundaries
Spearman ρ = 0.62 m = 0.83
Spearman ρ = 0.79 m = 0.89
Spearman ρ = 0.75 m = 1.01
Spearman ρ = 0.86 m = 1.02
Spearman ρ = 0.79 m = 1.13
Spearman ρ = 0.89 m = 1.1
D
Distributions of TAD boundary strengths
TAD boundary strength
E
Avg. contact conformation within/around TADs
log2(EC/hPSC)
F
Gene exp. in EC
FPKM
lost
gained
shared
G
TAD sizes by comp. type
Mb
H
Boundaries by stable, dynamic comp.
Counts
Proportions
A
B
A → B
B → A
A → B → A
B → A → B
I
Gene exp. in EC
FPKM

Figure S4. In differentiation, TAD boundaries converge on an endothelial cell state, and gained boundaries tend to be associated with repressive chromatin environments, related to Figure 4

A. Hi-C interaction heatmaps (40-kb bins, chromosome 17, approximately 59–69 Mb) showing dynamics of local interactions and TADs in hPSC, MES, EP, and EC. Horizontal solid bars: TADs; gaps in horizontal solid bars: TAD boundaries; bottom row: genes (green).

B. Hierarchically clustered heatmap for TAD-boundary set intersections for Hi-C samples using a window of ± 80 kb (40-kb resolution, autosomes).

C. Scatter plots for Hi-C sample (40-kb resolution, autosomes) insulation scores at TAD boundaries: hPSC versus MES, EP, and EC (top row); MES versus EP and EC (middle row); and EP versus EC (bottom row). ρ: Spearman correlation coefficients; m: regression slope; blue dashed line: regression line; black solid line: x = y.

D. Box-and-whisker plots imposed over violin plots showing TAD boundary strength distributions for Hi-C samples (40-kb resolution, autosomes). Box-and-whisker plots represent the 25th percentile, median, and 75th percentile; whiskers extend to 1.5 times the interquartile range. P-values from pairwise Kolmogorov-Smirnov tests adjusted with Benjamini-Hochberg post-hoc tests: * < 0.05, ** < 0.01, *** < 0.001.

E. $\log_2$ ratio heatmap for an EC aggregate TAD plot over an hPSC aggregate TAD plot (see Figure 3C); red: interaction frequency higher in EC; blue: interaction frequency higher in hPSC.

F. Box-and-whisker plots showing FPKM-normalized EC gene expression at TAD boundaries lost in differentiation (hPSC-specific), gained in differentiation (EC-specific), and shared between time points (hPSC and EC); FPKM values are averaged from two independent replicates. P-values from pairwise Kolmogorov-Smirnov tests adjusted with Benjamini-Hochberg post-hoc tests: *** < 0.001; if no asterisks, then not significant.

G. Box-and-whisker plots depicting TAD size distributions within A and B compartments across differentiation. P-values from intra-sample Kolmogorov-Smirnov tests adjusted with Benjamini-Hochberg post-hoc tests: *** < 0.001.

H. Stacked bar charts showing the absolute (top) and relative (bottom) numbers of lost, gained, and shared TAD boundaries stratified by stable (A, B) and dynamic (A-to-B, B-to-A, A-to-B-to-A, B-to-A-to-B) compartments. P-values from pairwise chi-squared contingency table tests: * < 0.05; if no asterisks, then not significant.

I. Box-and-whisker plots showing EC gene expression (FPKM; averaged from two independent replicates) distributions at lost, gained, and shared TAD boundaries stratified by stable and dynamic compartments. P-values from pairwise Kolmogorov-Smirnov tests and were adjusted with Benjamini-Hochberg post-hoc tests: * < 0.05, ** < 0.01, *** < 0.001; if no asterisks, then not significant.

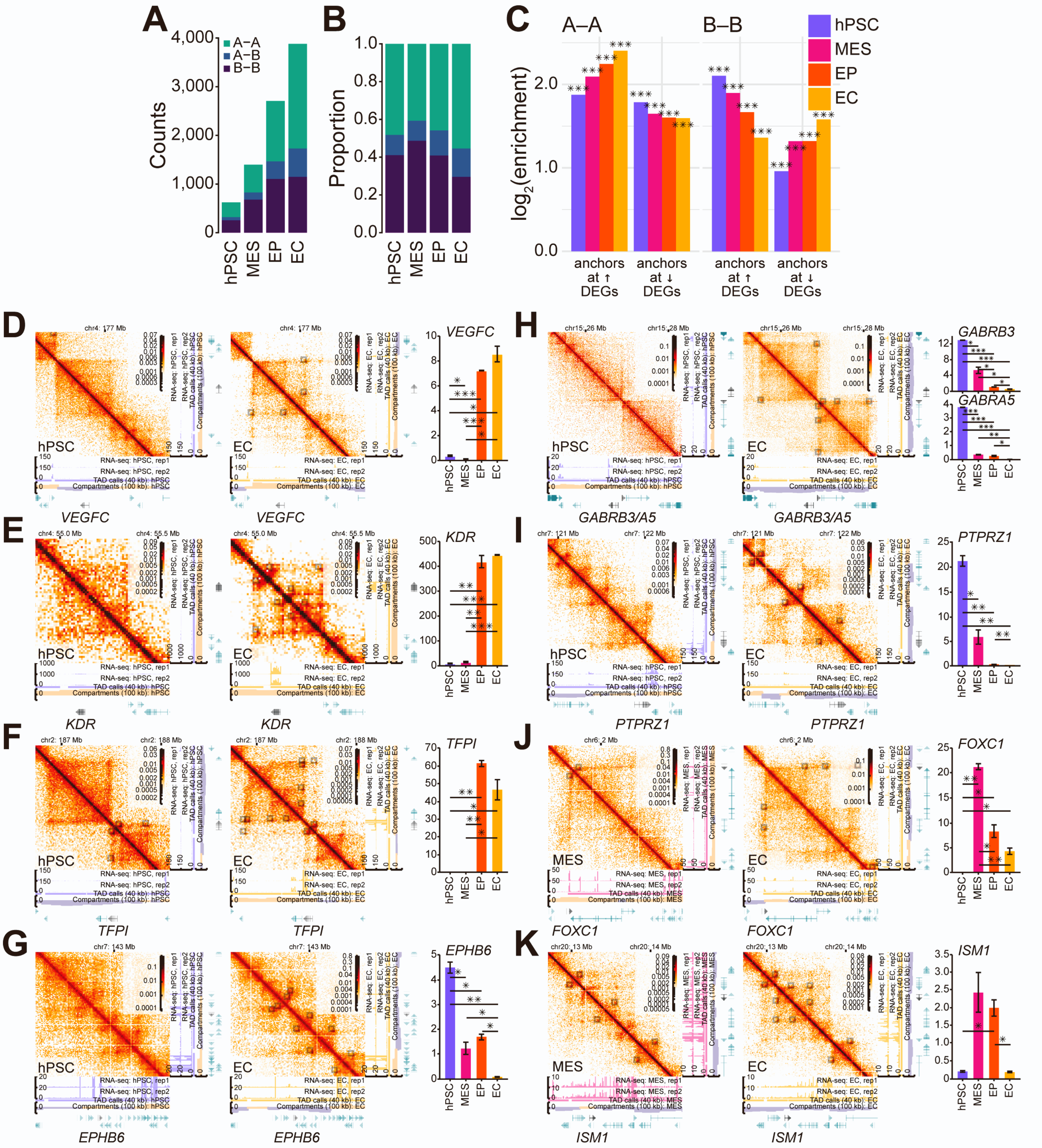

Alavattam, Mitzelfelt et al. – Figure S5

Figure S5. PPI anchors are enriched at sites of DEGs, and examples of PPIs associated with gene repression, related to Figure 5

A, B. Stacked bar charts showing the absolute (**A**) and relative (**B**) numbers of PPIs for Hi-C samples (10-kb resolution, autosomes). Bars are stratified by PPI-anchor compartment (100-kb resolution, autosomes) of origin; A–A: both anchors in A compartments; A–B: one anchor in A compartment, other anchor in B compartment; B–B, both anchors in B compartments.

C. Bar plots showing the $\log_2$ enrichment of PPI anchors at up- and downregulated differentially expressed genes (DEGs) stratified by compartment type in which anchors are found (A–A, A–B, and B–B; 100-kb resolution, autosomes) for Hi-C samples (10-kb resolution, autosomes). DEGs from DESeq2 analysis (Love et al., 2014) (adjusted p-value < 0.05, absolute $\log_2$ fold change > 1) of EC versus hPSC. Enrichment significantly different via chi-squared tests with Yates corrections: *** < 0.001.

D–K. Left: visualization of PPIs associated with *VEGFC* (**D**), *KDR* (**E**), *TFPI* (**F**) *EPHB6* (**G**), *GABRB3* and *GABRA5* (**H**), *PTPRZ1* (**I**), *FOXC1* (**J**), and *ISM1* (**K**) in hPSC or MES (left), and EC (right) Hi-C samples (10-kb bins, autosomes). Heatmaps of normalized Hi-C interaction frequencies (10-kb resolution), RNA-seq signal (unadjusted), TADs (40-kb resolution; see Methods), genomic compartments (100-kb resolution; gold: A compartment; purple: B compartment), and genes (green and black); solid squares overlying the heatmaps: PPIs. Right: bar plots for the RNA-seq expression levels (FPKM) of the above genes. P-values from pairwise t-tests between samples (two independent replicates each) adjusted with Benjamini-Hochberg post-hoc tests: * < 0.05, ** < 0.01, *** < 0.001. Bar: mean; error bars: standard error of the mean (SEM).

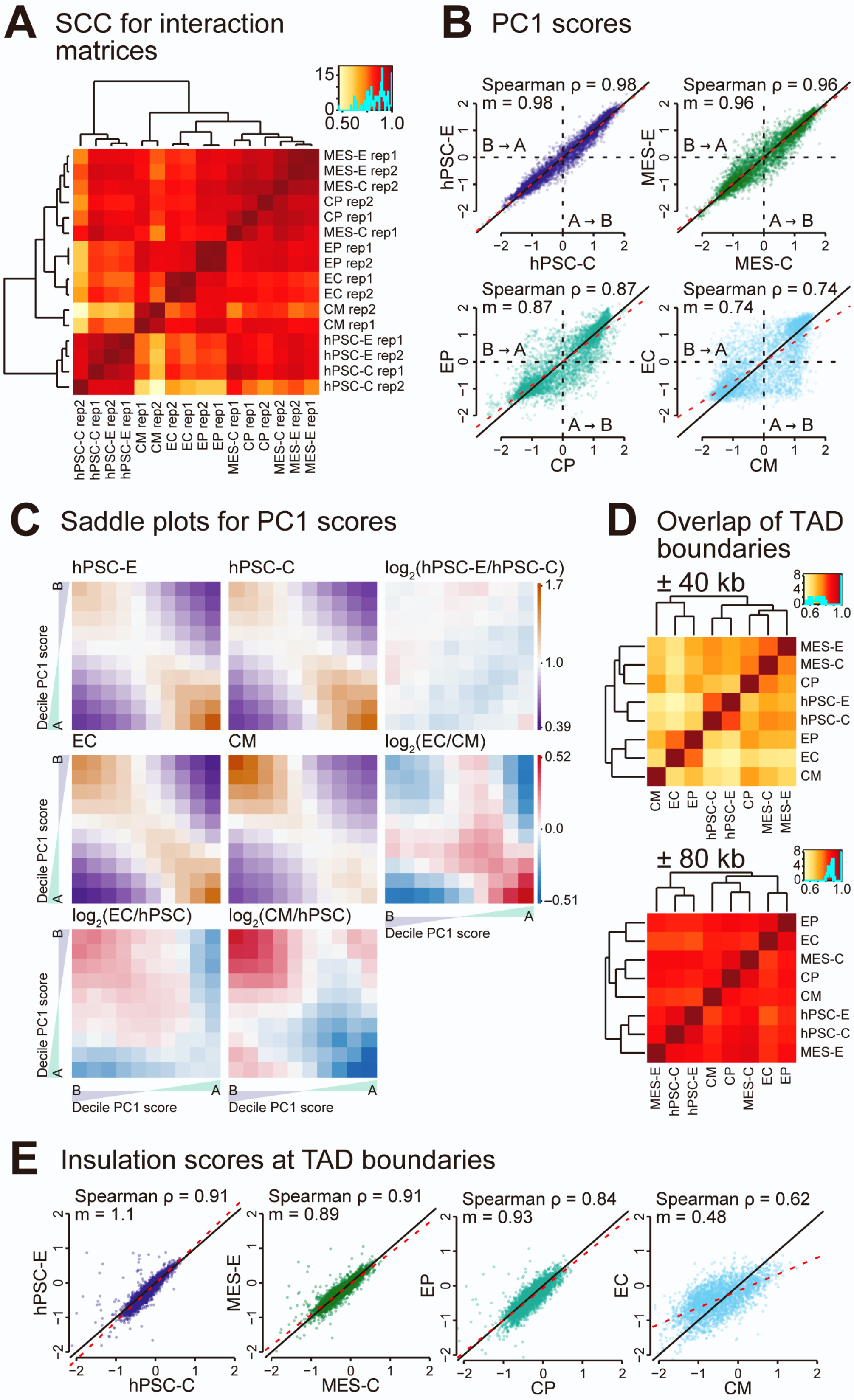
A SCC for interaction matrices
MES-E rep1
MES-E rep2
MES-C rep2
CP rep2
CP rep1
MES-C rep1
EP rep1
EP rep2
EC rep1
EC rep2
CM rep2
CM rep1
hPSC-E rep1
hPSC-E rep2
hPSC-C rep1
hPSC-C rep2
B PC1 scores
Spearman ρ = 0.98
m = 0.98
Spearman ρ = 0.96
m = 0.96
Spearman ρ = 0.87
m = 0.87
Spearman ρ = 0.74
m = 0.74
B → A
A → B
hPSC-E
hPSC-C
MES-E
MES-C
EP
CP
EC
CM
C Saddle plots for PC1 scores
hPSC-E
hPSC-C
log2(hPSC-E/hPSC-C)
EC
CM
log2(EC/CM)
log2(EC/hPSC)
log2(CM/hPSC)
Decile PC1 score
D Overlap of TAD boundaries
± 40 kb
± 80 kb
E Insulation scores at TAD boundaries
Spearman ρ = 0.91
m = 1.1
Spearman ρ = 0.91
m = 0.89
Spearman ρ = 0.84
m = 0.93
Spearman ρ = 0.62
m = 0.48

Figure S6. Chromatin topologies differ in endothelial cells versus cardiomyocytes, related to Figure 6

A. Hierarchically clustered heatmap of stratum-adjusted correlation coefficients (SCC) for Hi-C sample replicates (500-kb resolution, autosomes). hPSC-E: human pluripotent stem cells from endothelial cell differentiation; MES-E: mesoderm cells from endothelial cell differentiation; EP: endothelial progenitor cells ; EC: endothelial cells; hPSC-C: human pluripotent stem cells from cardiomyocyte differentiation; MES-C: mesoderm cells from cardiomyocyte differentiation; CP: cardiomyocyte progenitor cells; CM: cardiomyocytes.

B. Scatter plots for Hi-C sample (500-kb resolution, autosomes) PC1 scores from comparable time points in endothelial cell and cardiomyocyte differentiation: hPSC-E versus hPSC-C (top left), MES-E versus MES-C (top right), EP versus CP (bottom left), and EC versus CM (bottom right). ρ: Spearman correlation coefficient; m: regression slope; red dashed line: regression line; black solid line: x = y.

C. Saddle plots and $\log_2$ ratios of saddle plots for hPSC-E, hPSC-C, EC, and CM Hi-C samples (500-kb resolution, autosomes). Gold-to-purple color bar: observed/expected interactions. Red-to-blue color bar: $\log_2$ ratios of saddle plots; red: interactions higher in numerator; blue: interactions higher in denominator.

D. Hierarchically clustered heatmaps for TAD-boundary set intersections using windows of ± 40 kb (top) and ± 80 kb (bottom) around boundaries (Hi-C samples: 40-kb resolution, autosomes).

E. Scatter plots for Hi-C sample (40-kb resolution, autosomes) insulation scores from comparable time points in endothelial cell and cardiomyocyte differentiation: (left to right) hPSC-E versus hPSC-C, MES-E versus MES-C, EP versus CP, and EC versus CM. ρ: Spearman correlation coefficient; m: regression slope; red dashed line: regression line; black solid line: x = y.

Supplemental datasets

Dataset S1. RNA-seq and Hi-C dataset metrics, stratum-adjusted correlation coefficients, proportions of overlapping TAD boundaries, and numbers of DEGs associated with PPI anchors, related to Figures 1, 2, 4–6

- Tab 01 (related to Figure 1): Metrics for RNA-seq experiments.
- Tab 02 (related to Figure 2): Metrics for Hi-C experiments.
- Tab 03 (related to Figure 2): Stratum-adjusted correlation coefficients (SCCs) for endothelial cell Hi-C sample replicates (500-kb resolution, autosomes).
- Tab 04 (related to Figure 4): TAD boundary ± 40 kb (1 bin) intersections (top) and non-intersections (bottom) for endothelial cell Hi-C samples (40-kb resolution, autosomes).
- Tab 05 (related to Figure 4): Same as tab 04 except for TAD boundaries ± 80 kb (2 bins).
- Tab 06 (related to Figure 5): Counts for up- and downregulated DEGs associated with PPIs with anchors in A compartments, both compartments, and B compartments.
- Tab 07 (related to Figure 6): SCCs for both endothelial cell and cardiomyocyte Hi-C sample replicates.
- Tab 08 (related to Figure 6): Same as tab 04 except for both endothelial cell and cardiomyocyte Hi-C samples (40-kb resolution, autosomes).
- Tab 09 (related to Figure 6): Same as tab 08 except for TAD boundaries ± 80 kb.

Dataset S2. GO terms for PC loadings, analyses of differentially expressed genes (DEGs), DEGs associated with B-to-A and A-to-B compartment transitions, DEGs associated with shared TAD boundaries in stable A compartments, and DEGs associated with PPI anchors in A and B compartments, related to Figures 1, 3–5

Gene Ontology (GO) terms (molecular function, biological process, and cellular component) associated with the below data types. P-values from hypergeometric tests adjusted with Bonferroni corrections. List filtered for terms with adjusted p-value < 0.05.

- Tabs 01–04 (related to Figure 1): Top 500 PC1 negative loadings (tab 01), top 500 PC1 positive loadings (tab 02), top 500 PC2 negative loadings (tab 03), and top 500 PC2 positive loadings (tab 04)
- Tabs 05–16 (related to Figure 1): The up- and downregulated differentially expressed genes (DEGs) between…
  - mesoderm cells (MES) versus human pluripotent stem cells (hPSC; tabs 05, 06)
  - endothelial progenitor cells (EP) versus hPSC (tabs 07, 08)
  - endothelial cells (EC) versus hPSC (tabs 09, 10)
  - EP versus MES (tabs 11, 12)
  - EC versus MES (tabs 13, 14)
  - EC versus EP (tabs 15, 16)
- Tabs 17, 18 (related to Figure 3): The up- (tab 17) and downregulated (tab 18) DEGs between EC versus hPSC in regions that undergo, respectively, B-to-A and A-to-B compartment transitions
- Tabs 19, 20 (related to Figure 4): The up- (tab 19) and downregulated (tab 20) DEGs between EC versus hPSC in the vicinity (± 80 kb) of TAD boundaries in stable A compartments
- Tabs 21, 22 (related to Figure 5): The up- (tab 21) and downregulated (tab 22) DEGs between EC versus hPSC at, respectively, EC A–A and EC B–B PPI anchors

Supplemental notes

Note S1. Transcriptomes undergo overt, cell type-relevant changes in endothelial cell differentiation, related to Figure 1

We analyzed a panel of genes relevant to time point-specific cell functions and observed gene expression patterns consistent with endothelial specification (Figure S1D). Global analyses support this finding: Transcriptomes undergo overt changes in specification, with large amounts of differential expression (Figure S1E, F), and Gene Ontology (GO) analyses (Ashburner et al., 2000; Chen et al., 2009; Gene Ontology Consortium, 2021) of differentially expressed genes (DEGs) revealed biological processes highly relevant to corresponding cell types (Dataset S2). These results support the efficacy and reproducibility of our differentiation protocol, and underscore the dynamism of gene expression in endothelial cell development.

Note S2. Hi-C datasets are of high quality as indicated by read alignment metrics, analyses of insulation-score consistency, and evaluation of stratum-adjusted correlation coefficients, related to Figure 2

We observed the alignment of Hi-C reads at high rates (68–74%; Dataset S1) and high, comparable levels of *cis* interactions across differentiation (64–80%, Dataset S1). To further assess data quality, we calculated "insulation scores" (Crane et al., 2015) (Supplemental Experimental Procedures) for all replicates and compared these values within and between samples. Insulation scores are used to identify a form of chromatin organization known as "topologically associating domains" (discussed below). It is expected that topologically associating domains are harder to detect in noisy, low-quality datasets and, intuitively, such datasets should have a low degree of insulation-score consistency. Hierarchical clustering of Spearman correlation coefficients (ρ) for insulation scores showed high levels of concordance between replicates (Figure S2A); likewise, scatter plots revealed Spearman coefficients ≥0.92 and regression slopes ranging 0.84–1.01 (Figure S2B). Finally, we used the HiCRep method (Yang et al., 2017) to evaluate the consistency of biological replicates. Stratum-adjusted correlation coefficients (SCC) (Yang et al., 2017) revealed high levels of reproducibility between biological replicates (Dataset S1; Figure 2D, E). Together, these results indicate that our Hi-C datasets are of high quality.

Note S3. In differentiation, endothelial cell genes associate with PPIs in both eu- and heterochromatic regions, related to Figure 5

Examples of endothelial cell genes that associate with PPIs as their transcription increases in development include *VEGFC* (vascular endothelial growth factor C; Figure S5D), which codes for a protein critical for angiogenesis, endothelial cell growth, and blood vessel permeability in vascular and lymphatic vessels (Joukov et al., 1996; Jussila and Alitalo, 2002); *KDR* (kinase insert domain receptor; Figure S5E), which encodes a VEGF receptor (Terman et al., 1992); and *TFPI* (tissue factor pathway inhibitor; Figure S5F), a gene that encodes a serine protease inhibitor with anti-coagulative effects (Broze et al., 1990; Dahm et al., 2008; Ndonwi et al., 2010).

Numerous genes repressed in endothelial cell differentiation are associated with B-compartment PPIs, including genes associated with tissue patterning and neuronal development. These include *EPHB6* (ephrin type-B receptor 6; Figure S5G), which codes for a pseudokinase member of the Eph receptor family (Liang et al., 2021; Lisabeth et al., 2013; Nievergall et al., 2012; Wilkinson, 2014); *GABRB3* (gamma-aminobutyric acid type A receptor subunit beta 3) and *GABRA5* (gamma-aminobutyric acid type A receptor subunit alpha 3; Figure S5H), both of which encode receptor subunits for the neurotransmitter GABA; and *PTPRZ1* (protein tyrosine phosphatase receptor type Z1; Figure S5I), which codes for a

member of the receptor protein tyrosine phosphatase family that is largely restricted to the central nervous system in development (Wang et al., 2010).

Additionally, we observed examples of B-compartment PPI anchors associating with genes that code for factors with anti-angiogenic properties. Examples include *FOXC1* (forkhead box C1; Figure S5J), whose protein product has known antagonistic roles in vascular development (Koo and Kume, 2013); and *ISM1* (isthmin 1; Figure S5K), which codes for a secreted protein that functions as an endogenous angiogenesis inhibitor (Xiang et al., 2011).

Note S4. Additional comments and interpretation, related to Discussion

Contextualizing the increase in long-range cis contacts during endothelial cell differentiation. We observed a general, gross increase in long-range *cis* chromatin contacts as endothelial cells mature—a finding consistent with a number of studies of Hi-C (and Hi-C-like) data using *in vitro* and *in vivo* models of differentiation. These studies include stem cell differentiation to cells with neural identities (Bonev et al., 2017; Choi et al., 2020; Dixon et al., 2015; Fraser et al., 2015), studies of stem cell differentiation to cardiomyocytes (Bertero et al., 2019a, 2019b; Zhang et al., 2019), and studies of early mammalian development (Du et al., 2017; Ke et al., 2017), among others. However, in hematopoiesis, a form of cell lineage specification developmentally related to endothelial cell specification, there are decreases in the probabilities of long-range *cis* contacts in the development of megakaryocyte-erythrocyte progenitors from multipotent progenitors, megakaryocytes from megakaryocyte progenitors, and granulocytes from granulocyte-macrophage progenitors (Zhang et al., 2020). A drop in long-range *cis* contacts is also observed when germ cells mature into meiosis (Du et al., 2020; Wang et al., 2019), although long-range *cis* interactions increase as post-mitotic male germ cells mature (Alavattam et al., 2019; Vara et al., 2019; Wang et al., 2019). (Consistent with this, a marked drop occurs when proliferating cells enter into mitosis (Gibcus et al., 2018; Naumova et al., 2013).) Thus, the finding that there is an increase in long-range *cis* contacts during endothelial cell differentiation informs our collective assessment of the characteristics of differentiating cells.

TAD and PPI function is dependent on compartment type. While work remains to understand how TADs strengthen and PPIs arise in endothelial cells, our findings indicate that these features are influenced by stable and dynamic compartments to regulate transcription necessary for differentiation: strengthened TAD boundaries are accompanied by increased numbers of intra-TAD contacts, nascent TAD boundaries in A compartments are enriched for expressed genes and upregulated DEGs, and PPI anchors strongly correlate with upregulated DEGs in the A compartment, consistent with mounting evidence that loop-extruded features of chromatin organization play an important role in the regulation of gene expression (Bonev et al., 2017; Freire-Pritchett et al., 2017; Gorkin et al., 2014). We showed a number of examples of PPIs forming in A compartments coincident with the upregulation of genes essential to endothelial cell biology. On the other hand, PPI anchors and nascent TAD boundaries in B compartments are associated with lowly expressed and repressed genes, and downregulated DEGs. These findings indicate that the function of TADs and PPIs, two interrelated forms of chromatin organization, is contextual, dependent on compartment type; in stable and dynamic A compartments, they promote gene expression necessary for endothelial function.

Supplemental experimental procedures

Cell culture

To differentiate endothelial cells from human pluripotent stem cells, a modified version of a published protocol was followed (Palpant et al., 2015, 2017). Human pluripotent stem cells (hPSCs) from the RUES2 line (RUESe002-A; WiCell) were maintained on recombinant human Laminin-521 matrix (rhLaminin521; Biolamina) in Essential 8 (E8) media (ThermoFisher) at a density of 0.5 μg/cm^2. Cells were passaged with Versene (ThermoFisher) and seeded overnight with 10 μM Y-27632 (ROCK inhibitor; Tocris). Karyotyping was performed by Diagnostic Cytogenetics Incorporated, Seattle, WA, and cells were found to contain no clonal abnormalities (Figure S1A).

Prior to directed endothelial cell differentiation (day –1), hPSCs were re-seeded at a density of 3.0 × 10^5 cells per well of a 12-well plate coated with 2 μg/cm^2 rhLaminin521; then, the cells were immersed in E8 supplemented with 10 μM Y-27632. The following day (day 0), differentiation was induced with 7 μM CHIR99021 (a GSK3 inhibitor; Cayman) in RPMI (ThermoFisher) supplemented with 500 μg/mL BSA (Sigma, A9418) and 213 μg/mL ascorbic acid (Sigma, A8960). Seventy-two hours later (day 3), the media was switched to Stempro34 (Invitrogen) containing 300 ng/mL VEGF (Peprotech), 5 ng/mL bFGF (R&D), 10 ng/mL BMP4 (Preprotech), 4 × 10^{-4} M monothioglycerol (Sigma), 50 μg/mL ascorbic acid (Sigma), 2 mM L-glutamine (Invitrogen), and penicillin-streptomycin (Invitrogen). Forty-eight hours later (day 5), cells were passaged with 0.25% Trypsin (ThermoFisher) and re-seeded onto 0.2% gelatin-coated 10-cm dishes at a density of 5.0 × 10^5 cells per dish in Endothelial Cell Growth Medium (EGM; Lonza) containing 20 ng/mL VEGF, 20 ng/mL bFGF, and 1 μM CHIR99021. Cells were maintained on these dishes in EGM plus above-described factors until day 14 (Figure 1A).

Flow cytometry

To evaluate the purity of differentiating cells, cell aliquots were collected at days 6 and 14 of differentiation (Figure 1A). The cells were washed in Dulbecco's phosphate-buffered saline (DPBS) with 5% fetal bovine serum (FBS) and resuspended in a solution of Dulbecco's Modified Eagle's Medium (DMEM; Corning) containing mouse anti-human CD34-PerCP (BD Biosciences 340430, 1:5) and mouse anti-human CD31-FITC (BD Biosciences 555445, 1:5) antibodies, or a solution of DMEM and antibody-appropriate isotype controls. All staining was performed on live cells. Staining was performed on ice for 45 minutes in darkness. The stained cells were washed in DPBS and fixed with 4% paraformaldehyde (Affymetrix) prior to flow cytometry analyses. Flow cytometry was performed using a FACS Canto II cell analysis instrument (BD Biosciences); flow cytometry data were analyzed using FlowJo Software (FlowJo, LLC). Gates were set such that isotype controls contained 5% positive cells (Figure S1B, 1C).

Hi-C: Sample preparation, library generation, and sequencing

In situ DNase Hi-C (Ramani et al., 2016) was performed on 2–3 × 10^6 cells from two independent differentiations at the following time points: day 0, a pluripotent cell type (hPSC); day 2, a mesodermal cell type (MES); day 6, an endothelial progenitor cell type (EP); and day 14, an endothelial cell type (EC; Figure 1A). To prepare samples for Hi-C benchwork, plated cells were washed three times with DPBS and then fixed with a mixture of fresh RPMI containing 2% formaldehyde (diluted from a 37% formaldehyde solution); fixation took place for 10 minutes at room temperature with orbital rotation. Formaldehyde was quenched with 1% 2.5 M glycine for 5 minutes at room temperature and then 15 minutes at 4 °C. Afterwards, cells were treated with 0.25% trypsin for 5 minutes at 37 °C, washed with RPMI containing 10% FBS, and subsequently scraped off their plates. Cells were washed once with DPBS, flash frozen in liquid nitrogen, and stored at −80 °C until the time of Hi-C benchwork.

To perform *in situ* DNase Hi-C, frozen samples were thawed and lysed in 500 µL lysis buffer comprised of 10 mM Tris-HCl (pH 8.0), 10 mM NaCl, 0.2% Igepal CA-630, 1× protease inhibitor, and double-distilled water (ddH_2O). Then, nuclei were resuspended in 300 µL DNase buffer with 0.2% SDS and $MnCl_2$, and incubated at 37 °C for 60 minutes with periodic vortexing. Afterwards, nuclei were subjected to an additional 300 µL DNase buffer containing 2% Triton X-100 and RNase A, and incubated for another 10 minutes. Six units of DNase (ThermoFisher, EN0525) were added and incubated for 7 minutes at room temperature. The reaction was stopped with 30 µL of 0.5 M EDTA and 15 µL of 10% SDS. Nuclei were collected and resuspended in 150 µL water and combined with 300 µL AMPure XP beads (Beckman). DNA-end repair with T4 DNA Polymerase (ThermoFisher, EP0062) and Klenow (ThermoFisher, EP0052) was performed *in situ*, as was subsequent dA-tailing with Klenow Exo- (ThermoFisher, EP0422). Then, biotinylated oligonucleotides (adapters) were ligated to DNA at 16 °C overnight. To remove unbound adapters, nuclei were washed once with AMPure buffer (20% PEG in 2.5 M NaCl), then twice with 80% ethanol. To carry out adapter phosphorylation and ligation, PNK treatment was performed for 4 hours at room temperature. To de-crosslink the DNA, samples were treated with Proteinase K overnight at 62 °C. The next day, DNA precipitation was performed with 0.055 mg/mL glycogen, 10% volume 3 M NaOAc (pH 5.2), and 100% volume isopropanol for 2 hours at −80 °C. To purify the DNA, it was resuspended in 100 µL water and combined with 100 µL of AMPure beads. The pull-down of biotin adapter-containing DNA was performed using MyOne C1 Beads (ThermoFisher, 65001) for 30 minutes at room temperature with rotation. Afterwards, samples were washed four times with bind-and-wash buffer (5 mM Tris-HCl pH 8.0, 0.5 mM EDTA, 1 M NaCl, and 0.05% Tween-20) followed by two elution-buffer washes. On-bead DNA underwent end repair using the reagents in a Fast DNA End Repair Kit (ThermoFisher, K0771), and this was followed by dA-tailing using Klenow Exo- (ThermoFisher, EP0422); between each reaction, the DNA was washed four times with bind-and-wash buffer and twice with Tris-EDTA buffer.

Sequencing Y-adapters were ligated at room temperature for 60 minutes. To amplify the Hi-C libraries, the DNA underwent 10 PCR cycles using Kapa HiFi ReadyStart Master Mix (Roche, KK2602) with barcode-containing primers. Libraries were purified with 0.8× volumes of Ampure XP beads and quantified with a Qubit prior to sequencing. The libraries were paired-end sequenced using a NextSeq 500 (Illumina) in a high-output run with 150 cycles, 75 cycles for each end.

RNA-seq: Sample preparation, library generation, and sequencing

Cell samples from the same two independent differentiations were collected in Buffer RLT (QIAGEN) at the time points described above (Figure 1A). Samples were stored at –80 °C prior to RNA purification, which was performed with an RNeasy Mini Kit (QIAGEN) with on-column DNase digestion. RNA-seq libraries were prepared from total RNA (≥200 nucleotides in length) using the TruSeq Stranded Total RNA Ribo-Zero H/M/R kit (Illumina, RS-122-2201). Libraries were paired-end sequenced on a NextSeq 500 (Illumina) in a high-output run with 150 cycles, 75 cycles for each end.

Hi-C: Data-sourcing, alignment, processing, and quality control

In situ DNase Hi-C datasets for cardiomyocyte samples differentiated from RUES2 hPSCs were obtained from published work (GEO GSE106690) (Bertero et al., 2019a). As with the endothelial cell data generated for this study, the cardiomyocyte data are comprised of samples from two independent differentiations at the following time points: day 0, hPSC; day 2, MES; day 5, a cardiomyocyte progenitor cell type (CP); and day 14, a cardiomyocyte cell type (CM).

Reads were aligned to a *Homo sapiens* reference genome (Ensembl 83) with BWA-MEM (0.7.13-r1126) (Li, 2013; Li and Durbin, 2009) using default parameters, and each read-pair end was aligned individually. The Hi-C datasets exhibit high percentages of unique, paired alignments (Dataset S1). Primary alignments were extracted and sorted with Samtools (version 1.2) (Li et al., 2009). Then, the

alignments were processed with HiC-Pro (version 2.7.6) (Servant et al., 2015), filtering for MAPQ scores ≥ 30 and excluding read pairs that mapped within 1 kb of each other; PCR duplicates, defined as sequence matches with the exact same starts and ends, were excluded. HiC-Pro `allValidPairs` files and unbalanced matrices were generated at the following resolutions: 40, 100, and 500 kb.

Hi-C data quality were assessed with three metrics: proportions of *cis* interactions, insulation-score (described below) consistency, and contact-matrix similarity. Processed Hi-C data exhibited consistently high levels of *cis* interactions across differentiation: 64–80% (Dataset S1). Insulation score analyses of replicate samples revealed high levels of concordance between replicates (Figure S2A, B). To assess contact-matrix similarity, HiCRep analyses (Yang et al., 2017) were performed with the following parameters: `resol = 500000, ubr = 5000000, h = 1`; all other parameters were set to default values. HiCRep stratum-adjusted correlation coefficients revealed high levels of reproducibility between biological replicates (Dataset S1; Figures 2D, E).

To facilitate genomic binning at resolutions finer than 40 kb, biological replicates were merged using HiC-Pro, thereby increasing the sequencing depth for each sample. The merged Hi-C datasets are comprised of 111.4 million unique, valid read pairs for hPSC from endothelial cell differentiation; 120.0 million for MES from endothelial cell differentiation; 128.7 million for EP; 132.9 million for EC; 138.5 million for hPSC from cardiomyocyte differentiation; 143.3 million MES from cardiomyocyte differentiation; 161.6 million for CP; and 185.0 million for CM. HiC-Pro `allValidPairs` files and unbalanced matrices were generated for pooled replicates at the following resolutions: 10, 20, 40, 100, and 500 kb.

Using cooler (Abdennur and Mirny, 2019) and HiCExplorer (Ramírez et al.; Wolff et al., 2018, 2020) `hicConvertFormat`, HiC-Pro matrices were converted to the cooler format (`.cool`). Read pairs not aligned to autosomes or chromosome X were excluded from `.cool` files with HiCExplorer `hicAdjustMatrix`. Cooler-formatted matrices were balanced using Sinkhorn balancing (Sinkhorn and Knopp, 1967) such that the sum of every row and column is equal; to do so, the cooler `balance` command was called with default parameters.

Hi-C: Generation and visualization of Hi-C heatmaps

To generate and visualize chromatin-contact heatmaps, HiCExplorer `hicPlotMatrix` was used with contact matrices. To aid visualization, matrix values were log-transformed. To generate and visualize differential interactions between samples, matrices of $\log_2$ ratios matrices were generated with HiCExplorer `hicCompareMatrices` using `.cool` files from two datasets; the matrices of $\log_2$ ratios were plotted with `hicPlotMatrix`. To generate and visualize Pearson correlation coefficient heatmaps, contact matrices were input into HiCExplorer `hicPCA` for conversion to distance-normalized matrices (Lieberman-Aiden et al., 2009) (i.e., matrices taken from dividing observed interactions by expected interactions) and then Pearson correlation coefficient matrices; `hicPlotMatrix` was used to visualize the Pearson correlation coefficient matrices.

Hi-C: *Cis* contact-decay curve analyses

Cis contact-decay curves were generated by aggregating normalized counts as a function of distance at 500-kb intervals using HiCExplorer `hicPlotDistVsCounts`. Hi-C matrices were scaled to match the sum of the smallest matrix in the set. P-values were obtained from pairwise t-tests between samples (Figures 2C; Figure 6B) and were adjusted for false discovery rate with the Benjamini-Hochberg method.

Hi-C: Genomic compartment analyses

To calculate principal component (PC) scores for genomic compartment assignments, contact matrices were distance-normalized, transformed into Pearson correlation coefficient matrices, and eigen-decomposed using HOMER (Heinz et al., 2010; Lin et al., 2012). HiC-Pro `validPairs` files were

used as inputs. Eigenvectors were generated for contact matrices at 100- and 500-kb resolutions. Eigenvectors were assessed, and the first PC (PC1) was found to represent the genomic compartment profile (data not shown); subsequent principal components represented profiles distinct from genomic compartments (data not shown). Per convention (Lieberman-Aiden et al., 2009), PC1 orientation and binwise genomic-compartment assignments were based on biological features: bins with higher gene densities and increased enrichment for transcription were assigned to the "A" compartment type; all other bins were assigned to the "B" compartment type. Using oriented PC1 scores, sample similarity was evaluated via Spearman correlation coefficient and multidimensional scaling analyses (described below; Figure S3B, C; Figure 6D, E).

Genomic compartment transitions were defined using the following two criteria: (i) for a given bin, PC1 scores were available for each sample (i.e., no sample is represented by NA at that bin); and (ii) for a given bin, at least one sample had a mean PC1 score greater than 0 and at least one sample had a mean PC1 score less than 0. A-to-B-to-A-to-B and B-to-A-to-B-to-A transitions represented less than 1% of genomic compartment-switch regions (Figure 3B) and were combined, respectively, with A-to-B- and B-to-A-transitioning regions for downstream analyses.

Saddle plots were generated by ranking each bin, assigning it to its corresponding percentile value, and then dividing the genome into deciles. Each interaction (observed) was normalized to the average score at the corresponding distance for *cis* interactions (expected), then assigned to a decile pair based on the two bins. Plots represent the $\log_2$ average observed/expected values for pairs of deciles. Changes between one sample and another (e.g., EC/hPSC in Figure 3I) are represented by a $\log_2$ transformation of the quotient.

Hi-C: Multidimensional scaling

To perform multidimensional scaling (MDS) (Kruskal and Wish, 1977) of Hi-C interaction matrices, one minus the stratum-adjusted correlation coefficient (SCC), a statistic calculated by HiCRep (Yang et al., 2017), was used as input. To perform MDS of PC1 scores, one minus the Spearman correlation coefficient was used as input.

Hi-C: Analyses of topologically associating domains

Using the insulation score method (Crane et al., 2015), topologically associating domains (TADs) were called for Hi-C at 40-kb resolution. To do so, cworld (github.com/dekkerlab/cworld-dekker) `matrix2insulation.pl` was called with the following parameters: `--is 520001 --ids 320001 --ss 160001 --nt 0.01 --im mean`. Insulation scores are defined as the average number of chromatin contacts across a given bin, and TAD boundaries are called at local insulation score minima, which represent areas where the average number of chromatin contacts across a given bin are few (Crane et al., 2015). Aggregate TAD heatmaps were generated using FAN-C (Kruse et al., 2020) `fanc aggregate` with the following parameters: `--tads-imakaev --vmin 0.02 --vmax 0.075`. A given boundary was categorized as "shared" if its midpoint was observed at the same location ± 40 kb or 80 kb, i.e., within up to ± one or two bins of the boundary; if boundaries did not meet these conditions, then they were categorized as sample-specific and described as "lost" if unique to the sample from earlier in differentiation (e.g., hPSC) and "gained" if unique to the later sample (e.g., EC).

TADs were categorized as being in A or B compartments if they occupied one of the two compartment types (Figure S4G); if a given TAD overlapped multiple compartments, then it was assigned to the compartment type that it overlapped the most.

Hi-C: Enrichment of topologically associating domain boundaries with respect to genomic compartments

To calculate the enrichment of TAD boundary types (lost, gained, and shared) with respect to stable (A, B) and dynamic (A-to-B, B-to-A, A-to-B-to-A, B-to-A-to-B) compartment categories, the following calculation was performed:

$$\frac{\frac{x}{y}}{\frac{z}{a}},$$

where *x* is the number of a given boundary type within a given compartment category, *y* is the number of the given boundary type across all compartment categories, *z* is the number of all boundaries within the given compartment category, and *a* is the number of all boundaries across all compartment categories. P-values were obtained from chi-squared tests with Yates corrections.

Hi-C: Analyses of pairwise point interactions

Pairwise point interactions (PPIs) were called at 10-, 20-, and 40-kb resolutions using HiCCUPS (Rao et al., 2014), which was invoked with the following parameters: `-m 512 -r 10000,20000,40000 -k KR -f .1,.1,.1 -p 4,2,1 -i 8,4,2 -t 0.02,1.5,1.75,2 -d 20000,40000,80000 --cpu`. UpSet plots were generated to score the overlap of loop anchors among samples (Conway et al., 2017; Lex et al., 2014).

RNA-seq: Sourcing, alignment, and gene-level quantification of alignments

Reads were mapped to hg38 using HISAT2 (version 2.1.0) (Pertea et al., 2016) with default settings. The RNA-seq datasets exhibit high percentages of unique, paired alignments (Dataset S1). Files in `.sam` format were converted into coordinate-sorted `.bam` files using sambamba (version 0.6.6) (Tarasov et al., 2015) with default parameters. The program featureCounts from the Subread package (version 1.6.3) (Liao et al., 2019) was used to quantify gene expression levels with the following parameters specified: `-p -B -a gtf_file -t exon -g gene_id`.

RNA-seq: Principal component analysis

Principal component analysis (PCA) was performed with the software PCAtools (version 3.15; github.com/kevinblighe/PCAtools). An unfiltered RNA-seq counts matrix for the eight endothelial cell replicates—two independent replicates for each of four samples—was normalized with the DESeq2 function `rlog` (Love et al., 2014) prior to performing PCA. To determine significant principal components (PCs), Horn's parallel analysis (Horn, 1965) was performed using the PCAtools function `parallelPCA` with default settings. To determine the top positive and negative loading vectors for PCs 1 and 2, a PCAtools-generated loadings matrix was ordered by sign and magnitude prior to selecting the top 10 (Figure 1D) and top 500 (Figure 1E) component loadings.

RNA-seq: Gene expression analysis

To evaluate the expression of selected genes in endothelial cell differentiation (Figures 3–5; Figures S1, S5), the RNA-seq counts matrix was FPKM (fragments per kilobase of transcript per million mapped fragments)-normalized. Bar charts were plotted for genes of interest, and statistical significance was assigned through pairwise t-tests with false discovery rate adjustment (Benjamini-Hochberg method). Distributions of FPKM-normalized gene expression were plotted for genes associated with stable (A, B) and dynamic (B-to-A, A-to-B, A-to-B-to-A, B-to-A-to-B) compartment categories (Figure S3E); TAD boundary types, i.e., boundaries lost, gained, and shared in endothelial cell differentiation (Figure S4F); and TAD boundary types stratified by stable and dynamic compartment categories (Figure S4I).

Background distributions for FPKM-normalized gene expression were plotted for numbers of randomly sampled (non-repeating) genes equal to the numbers of genes in corresponding observed distributions (Figure S3E). P-values were obtained from pairwise Kolmogorov-Smirnov tests adjusted with Benjamini-Hochberg post-hoc tests; for Figure S3E, only p-values from comparisons between observed and background distributions are shown.

RNA-seq: Differential gene expression analysis

Differential gene expression analysis was performed with DESeq2 (version 1.32.0) (Love et al., 2014). Genes exhibiting a false discovery rate-adjusted p-value (Benjamini-Hochberg method) < 0.05 and an absolute $\log_2$ fold change > 1 were categorized as DEGs. EnhancedVolcano (version 1.14; github.com/kevinblighe/EnhancedVolcano) was used to generate labeled volcano plots (Figure S1E). A hierarchically clustered heatmap for $\log_2$ FPKM-normalized gene expression was generated for all non-redundant differentially expressed genes identified from pairwise analyses of endothelial cell samples (Figure S1F).

RNA-seq: Gene Ontology analyses

Gene Ontology (Ashburner et al., 2000; Chen et al., 2009; Gene Ontology Consortium, 2021) term enrichment analyses were performed using the ToppGene Suite ToppFun application (Chen et al., 2009); default settings were used, and the full gene set for each category was used as the background set. P-values were obtained from hypergeometric tests and adjusted for family-wise error rate with Bonferroni corrections.

RNA-seq: Enrichment of differentially expressed genes with respect to genomic compartments

To calculate enrichment scores for DEGs with respect to stable (A, B) and dynamic (A-to-B, B-to-A, A-to-B-to-A, B-to-A-to-B) compartment categories, the following calculation was performed:

$$\frac{\frac{x}{y}}{\frac{d}{g}},$$

where *x* is the number of DEGs within a given compartment category, *y* is the number of genes within the given compartment category, *d* is the number of all DEGs, and *g* is the number of all genes. P-values were obtained from chi-squared tests with Yates corrections.

RNA-seq: Enrichment of differentially expressed genes with respect to topologically associating domain boundaries

To calculate enrichment scores for DEGs with respect to TAD boundary types (lost, gained, and shared), the following calculation was performed:

$$\frac{\frac{x}{y}}{\frac{d}{g}},$$

where *x* is the number of DEGs within ± 80 kb (i.e., ± two bins) of a given boundary type, *y* is the number of genes within ± 80 kb of the given boundary type, *d* is the number of all DEGs, and *g* is the number of all genes.

To calculate enrichment scores for up- and downregulated DEGs with respect to TAD boundary types stratified by stable and dynamic compartment categories, the above calculation was performed except *x* is the number of up- or downregulated DEGs within ± 80 kb of a given boundary type within a

given compartment category, *y* is the number of up- or downregulated genes within ± 80 kb of the given boundary type within the given compartment category, *d* is the number of all up- or downregulated DEGs, and *g* is the number of all genes. P-values were obtained from chi-squared tests with Yates corrections.

RNA-seq: Enrichment of differentially expressed genes with respect to pairwise point interactions

To calculate enrichment scores for genes that overlap PPI anchors, the following calculation was performed:

$$\frac{\frac{x}{d}}{\frac{y}{g}},$$

where *x* is the number of DEGs overlapping anchors, *d* is the total number of DEGs, *y* is the number of genes overlapping anchors, and *g* is the total number of genes.

We used the following equation to calculate enrichment scores for anchors that overlap genes:

$$\frac{x}{a \cdot \frac{d}{g}},$$

where *x* is the number of anchors overlapping DEGs, *a* is the total number of anchors, *d* is the total number of DEGs, and *g* is the total number of genes. P-values were obtained from chi-squared tests with Yates corrections.

Statistics

Strategies for stratification, sampling, and enrichment are described in the following *Supplemental Experimental Procedures* subsections: for statistical analyses of features with respect to stratified genomic compartments (i.e., those that remain stable or undergo one of four transitions in differentiation), see *Hi-C: Genomic compartment analyses*; for statistical analyses of features with respect to stratified TAD boundaries (i.e., those that are lost, gained, and shared in differentiation), see *Hi-C: Analyses of topologically associating domains*; for statistical analyses of observed versus background gene expression distributions, see *RNA-seq: Gene expression analysis*; for statistical analyses of DEGs, see *RNA-seq: Differential gene expression analysis*; and for statistical analyses of Gene Ontology term enrichment, see *RNA-seq: Gene Ontology analyses*. Statistical analyses of enrichment of (a) general gene expression and (b) DEGs with respect to stratified genomic compartments, TAD boundary types, PPIs, and combinations thereof, are found in the following subsections: *RNA-seq: Gene expression analysis*, *RNA-seq: Enrichment of differentially expressed genes with respect to genomic compartments*, *RNA-seq: Enrichment of differentially expressed genes with respect to topologically associating domain boundaries*, and *RNA-seq: Enrichment of differentially expressed genes with respect to pairwise point interactions*.

Specific statistical tests are described in the *Results* and *Supplemental Experimental Procedures* sections, and figure and supplemental figure captions. An overview of statistical tests used in this study follows. Student's t-tests were performed for Figures 2C, 3G, 3H, 4I, 4J, 5F, 6B, S1D, and S5D–K; t-tests were adjusted for false discovery rate using the Benjamini-Hochberg method. Chi-squared tests with Yates corrections were performed for Figures 3D, 4D–F, 5C, S4H, and S5C. Kolmogorov-Smirnov tests were performed for Figures S3E, S4D, S4F, S4G, and S4I; Kolmogorov-Smirnov tests were adjusted for false discovery rate using the Benjamini-Hochberg method. Hypergeometric tests were performed for Figures 1E, 3E, 3F, 4G, 4H, 5D, and 5E; hypergeometric tests were adjusted for family-wise error rate with Bonferroni corrections.

Figure preparation

Plots were generated with, alone or in combination, Excel (version 16.60, Microsoft), base R (version 4.1), the R software package ggplot2 (version 3.3.4), and various plotting programs employed by the other software packages used in this study. Illustrator (version 26.0.2, Adobe) was used for composing figures.

Supplemental references

Abdennur, N., and Mirny, L.A. (2019). Cooler: scalable storage for Hi-C data and other genomically labeled arrays. Bioinformatics https://doi.org/10.1093/bioinformatics/btz540.

Alavattam, K.G., Maezawa, S., Sakashita, A., Khoury, H., Barski, A., Kaplan, N., and Namekawa, S.H. (2019). Attenuated chromatin compartmentalization in meiosis and its maturation in sperm development. Nat. Struct. Mol. Biol. *26*, 175–184.

Ashburner, M., Ball, C.A., Blake, J.A., Botstein, D., Butler, H., Cherry, J.M., Davis, A.P., Dolinski, K., Dwight, S.S., Eppig, J.T., et al. (2000). Gene ontology: tool for the unification of biology. The Gene Ontology Consortium. Nat. Genet. *25*, 25–29.

Bertero, A., Fields, P.A., Ramani, V., Bonora, G., Yardimci, G.G., Reinecke, H., Pabon, L., Noble, W.S., Shendure, J., and Murry, C.E. (2019a). Dynamics of genome reorganization during human cardiogenesis reveal an RBM20-dependent splicing factory. Nat. Commun. *10*, 1538.

Bertero, A., Fields, P.A., Smith, A.S.T., Leonard, A., Beussman, K., Sniadecki, N.J., Kim, D.-H., Tse, H.-F., Pabon, L., Shendure, J., et al. (2019b). Chromatin compartment dynamics in a haploinsufficient model of cardiac laminopathy. J. Cell Biol. *218*, 2919–2944.

Bonev, B., Mendelson Cohen, N., Szabo, Q., Fritsch, L., Papadopoulos, G.L., Lubling, Y., Xu, X., Lv, X., Hugnot, J.-P., Tanay, A., et al. (2017). Multiscale 3D Genome Rewiring during Mouse Neural Development. Cell *171*, 557–572.e24.

Broze, G.J., Jr, Girard, T.J., and Novotny, W.F. (1990). Regulation of coagulation by a multivalent Kunitz-type inhibitor. Biochemistry *29*, 7539–7546.

Chen, J., Bardes, E.E., Aronow, B.J., and Jegga, A.G. (2009). ToppGene Suite for gene list enrichment analysis and candidate gene prioritization. Nucleic Acids Research *37*, W305–W311. https://doi.org/10.1093/nar/gkp427.

Choi, W.-Y., Hwang, J.-H., Lee, J.-Y., Cho, A.-N., Lee, A.J., Jung, I., Cho, S.-W., Kim, L.K., and Kim, Y.-J. (2020). Chromatin Interaction Changes during the iPSC-NPC Model to Facilitate the Study of Biologically Significant Genes Involved in Differentiation. Genes *11*. https://doi.org/10.3390/genes11101176.

Conway, J.R., Lex, A., and Gehlenborg, N. (2017). UpSetR: an R package for the visualization of intersecting sets and their properties. Bioinformatics *33*, 2938–2940. https://doi.org/10.1093/bioinformatics/btx364.

Crane, E., Bian, Q., McCord, R.P., Lajoie, B.R., Wheeler, B.S., Ralston, E.J., Uzawa, S., Dekker, J., and Meyer, B.J. (2015). Condensin-driven remodelling of X chromosome topology during dosage compensation. Nature *523*, 240–244.

Dahm, A.E.A., Sandset, P.M., and Rosendaal, F.R. (2008). The association between protein S levels and anticoagulant activity of tissue factor pathway inhibitor type 1. J. Thromb. Haemost. *6*, 393–395.

Dixon, J.R., Jung, I., Selvaraj, S., Shen, Y., Antosiewicz-Bourget, J.E., Lee, A.Y., Ye, Z., Kim, A., Rajagopal, N., Xie, W., et al. (2015). Chromatin architecture reorganization during stem cell differentiation. Nature *518*, 331–336.

Du, Z., Zheng, H., Huang, B., Ma, R., Wu, J., Zhang, X., He, J., Xiang, Y., Wang, Q., Li, Y., et al. (2017). Allelic reprogramming of 3D chromatin architecture during early mammalian development. Nature *547*, 232–235.

Du, Z., Zheng, H., Kawamura, Y.K., Zhang, K., Gassler, J., Powell, S., Xu, Q., Lin, Z., Xu, K., Zhou, Q., et al. (2020). Polycomb Group Proteins Regulate Chromatin Architecture in Mouse Oocytes and Early

Embryos. Mol. Cell *77*, 825–839.e7.

Fraser, J., Ferrai, C., Chiariello, A.M., Schueler, M., Rito, T., Laudanno, G., Barbieri, M., Moore, B.L., Kraemer, D.C.A., Aitken, S., et al. (2015). Hierarchical folding and reorganization of chromosomes are linked to transcriptional changes in cellular differentiation. Mol. Syst. Biol. *11*, 852.

Freire-Pritchett, P., Schoenfelder, S., Várnai, C., Wingett, S.W., Cairns, J., Collier, A.J., García-Vílchez, R., Furlan-Magaril, M., Osborne, C.S., Fraser, P., et al. (2017). Global reorganisation of cis-regulatory units upon lineage commitment of human embryonic stem cells. Elife *6*. https://doi.org/10.7554/eLife.21926.

Gene Ontology Consortium (2021). The Gene Ontology resource: enriching a GOld mine. Nucleic Acids Res. *49*, D325–D334.

Gibcus, J.H., Samejima, K., Goloborodko, A., Samejima, I., Naumova, N., Nuebler, J., Kanemaki, M.T., Xie, L., Paulson, J.R., Earnshaw, W.C., et al. (2018). A pathway for mitotic chromosome formation. Science *359*, eaao6135.

Gorkin, D.U., Leung, D., and Ren, B. (2014). The 3D genome in transcriptional regulation and pluripotency. Cell Stem Cell *14*, 762–775.

Heinz, S., Benner, C., Spann, N., Bertolino, E., Lin, Y.C., Laslo, P., Cheng, J.X., Murre, C., Singh, H., and Glass, C.K. (2010). Simple Combinations of Lineage-Determining Transcription Factors Prime cis-Regulatory Elements Required for Macrophage and B Cell Identities. Molecular Cell *38*, 576–589. https://doi.org/10.1016/j.molcel.2010.05.004.

Horn, J.L. (1965). A rationale and test for the number of factors in factor analysis. Psychometrika 30, 179–185.

Joukov, V., Pajusola, K., Kaipainen, A., Chilov, D., Lahtinen, I., Kukk, E., Saksela, O., Kalkkinen, N., and Alitalo, K. (1996). A novel vascular endothelial growth factor, VEGF-C, is a ligand for the Flt4 (VEGFR-3) and KDR (VEGFR-2) receptor tyrosine kinases. The EMBO Journal *15*, 290–298. https://doi.org/10.1002/j.1460-2075.1996.tb00359.x.

Jussila, L., and Alitalo, K. (2002). Vascular growth factors and lymphangiogenesis. Physiol. Rev. *82*, 673–700.

Ke, Y., Xu, Y., Chen, X., Feng, S., Liu, Z., Sun, Y., Yao, X., Li, F., Zhu, W., Gao, L., et al. (2017). 3D Chromatin Structures of Mature Gametes and Structural Reprogramming during Mammalian Embryogenesis. Cell *170*, 367–381.e20.

Koo, H.-Y., and Kume, T. (2013). FoxC1-dependent regulation of vascular endothelial growth factor signaling in corneal avascularity. Trends Cardiovasc. Med. *23*, 1–4.

Kruse, K., Hug, C.B., and Vaquerizas, J.M. (2020). FAN-C: a feature-rich framework for the analysis and visualisation of chromosome conformation capture data. Genome Biology *21*. https://doi.org/10.1186/s13059-020-02215-9.

Kruskal J.B., and Wish M. (1977). Multidimensional Scaling. Sage Publications, Beverly Hills, CA.

Lex, A., Gehlenborg, N., Strobelt, H., Vuillemot, R., and Pfister, H. (2014). UpSet: Visualization of Intersecting Sets. IEEE Transactions on Visualization and Computer Graphics *20*, 1983–1992. https://doi.org/10.1109/tvcg.2014.2346248.

Li, H. (2013). Aligning sequence reads, clone sequences and assembly contigs with BWA-MEM. https://doi.org/10.48550/ARXIV.1303.3997.

Li, H., and Durbin, R. (2009). Fast and accurate short read alignment with Burrows-Wheeler transform.

Bioinformatics *25*, 1754–1760. https://doi.org/10.1093/bioinformatics/btp324.

Li, H., Handsaker, B., Wysoker, A., Fennell, T., Ruan, J., Homer, N., Marth, G., Abecasis, G., Durbin, R., and 1000 Genome Project Data Processing Subgroup (2009). The Sequence Alignment/Map format and SAMtools. Bioinformatics *25*, 2078–2079.

Liang, L.-Y., Roy, M., Horne, C.R., Sandow, J.J., Surudoi, M., Dagley, L.F., Young, S.N., Dite, T., Babon, J.J., Janes, P.W., et al. (2021). The intracellular domains of the EphB6 and EphA10 receptor tyrosine pseudokinases function as dynamic signalling hubs. Biochem. J *478*, 3351–3371.

Liao, Y., Smyth, G.K., and Shi, W. (2019). The R package Rsubread is easier, faster, cheaper and better for alignment and quantification of RNA sequencing reads. Nucleic Acids Research *47*, e47–e47. https://doi.org/10.1093/nar/gkz114.

Lieberman-Aiden, E., van Berkum, N.L., Williams, L., Imakaev, M., Ragoczy, T., Telling, A., Amit, I., Lajoie, B.R., Sabo, P.J., Dorschner, M.O., et al. (2009). Comprehensive mapping of long-range interactions reveals folding principles of the human genome. Science *326*, 289–293.

Lin, Y.C., Benner, C., Mansson, R., Heinz, S., Miyazaki, K., Miyazaki, M., Chandra, V., Bossen, C., Glass, C.K., and Murre, C. (2012). Global changes in the nuclear positioning of genes and intra- and interdomain genomic interactions that orchestrate B cell fate. Nature Immunology *13*, 1196–1204. https://doi.org/10.1038/ni.2432.

Lisabeth, E.M., Falivelli, G., and Pasquale, E.B. (2013). Eph Receptor Signaling and Ephrins. Cold Spring Harbor Perspectives in Biology *5*, a009159–a009159. https://doi.org/10.1101/cshperspect.a009159.

Love, M.I., Huber, W., and Anders, S. (2014). Moderated estimation of fold change and dispersion for RNA-seq data with DESeq2. Genome Biol. *15*, 550.

Naumova, N., Imakaev, M., Fudenberg, G., Zhan, Y., Lajoie, B.R., Mirny, L.A., and Dekker, J. (2013). Organization of the mitotic chromosome. Science *342*, 948–953.

Ndonwi, M., Tuley, E.A., and Broze, G.J. (2010). The Kunitz-3 domain of TFPI-α is required for protein S–dependent enhancement of factor Xa inhibition. Blood *116*, 1344–1351.

Nievergall, E., Lackmann, M., and Janes, P.W. (2012). Eph-dependent cell-cell adhesion and segregation in development and cancer. Cell. Mol. Life Sci. *69*, 1813–1842.

Palpant, N.J., Pabon, L., Roberts, M., Hadland, B., Jones, D., Jones, C., Moon, R.T., Ruzzo, W.L., Bernstein, I., Zheng, Y., et al. (2015). Inhibition of β-catenin signaling respecifies anterior-like endothelium into beating human cardiomyocytes. Development *142*, 3198–3209.

Palpant, N.J., Pabon, L., Friedman, C.E., Roberts, M., Hadland, B., Zaunbrecher, R.J., Bernstein, I., Zheng, Y., and Murry, C.E. (2017). Generating high-purity cardiac and endothelial derivatives from patterned mesoderm using human pluripotent stem cells. Nat. Protoc. *12*, 15–31.

Pertea, M., Kim, D., Pertea, G.M., Leek, J.T., and Salzberg, S.L. (2016). Transcript-level expression analysis of RNA-seq experiments with HISAT, StringTie and Ballgown. Nature Protocols *11*, 1650–1667. https://doi.org/10.1038/nprot.2016.095.

Ramani, V., Cusanovich, D.A., Hause, R.J., Ma, W., Qiu, R., Deng, X., Blau, C.A., Disteche, C.M., Noble, W.S., Shendure, J., et al. (2016). Mapping 3D genome architecture through in situ DNase Hi-C. Nat. Protoc. *11*, 2104–2121.

Ramírez, F., Bhardwaj, V., Villaveces, J., Arrigoni, L., Grüning, B.A., Lam, K.C., Habermann, B., Akhtar, A., and Manke, T. High-resolution TADs reveal DNA sequences underlying genome organization in flies. https://doi.org/10.1101/115063.

Rao, S.S.P., Huntley, M.H., Durand, N.C., Stamenova, E.K., Bochkov, I.D., Robinson, J.T., Sanborn, A.L., Machol, I., Omer, A.D., Lander, E.S., et al. (2014). A 3D Map of the Human Genome at Kilobase Resolution Reveals Principles of Chromatin Looping. Cell *159*, 1665–1680. https://doi.org/10.1016/j.cell.2014.11.021.

Servant, N., Varoquaux, N., Lajoie, B.R., Viara, E., Chen, C.-J., Vert, J.-P., Heard, E., Dekker, J., and Barillot, E. (2015). HiC-Pro: an optimized and flexible pipeline for Hi-C data processing. Genome Biology *16*. https://doi.org/10.1186/s13059-015-0831-x.

Sinkhorn, R., and Knopp, P. (1967). Concerning nonnegative matrices and doubly stochastic matrices. Pacific Journal of Mathematics *21*, 343–348. https://doi.org/10.2140/pjm.1967.21.343.

Tarasov, A., Vilella, A.J., Cuppen, E., Nijman, I.J., and Prins, P. (2015). Sambamba: fast processing of NGS alignment formats. Bioinformatics *31*, 2032–2034. https://doi.org/10.1093/bioinformatics/btv098.

Terman, B.I., Dougher-Vermazen, M., Carrion, M.E., Dimitrov, D., Armellino, D.C., Gospodarowicz, D., and Böhlen, P. (1992). Identification of the KDR tyrosine kinase as a receptor for vascular endothelial cell growth factor. Biochem. Biophys. Res. Commun. *187*, 1579–1586.

Vara, C., Paytuví-Gallart, A., Cuartero, Y., Le Dily, F., Garcia, F., Salvà-Castro, J., Gómez-H, L., Julià, E., Moutinho, C., Aiese Cigliano, R., et al. (2019). Three-Dimensional Genomic Structure and Cohesin Occupancy Correlate with Transcriptional Activity during Spermatogenesis. Cell Rep. *28*, 352–367.e9.

Wang, V., Davis, D.A., Veeranna, R.P., Haque, M., and Yarchoan, R. (2010). Characterization of the activation of protein tyrosine phosphatase, receptor-type, Z polypeptide 1 (PTPRZ1) by hypoxia inducible factor-2 alpha. PLoS One *5*, e9641.

Wang, Y., Wang, H., Zhang, Y., Du, Z., Si, W., Fan, S., Qin, D., Wang, M., Duan, Y., Li, L., et al. (2019). Reprogramming of Meiotic Chromatin Architecture during Spermatogenesis. Mol. Cell *73*, 547–561.e6.

Wilkinson, D.G. (2014). Regulation of cell differentiation by Eph receptor and ephrin signaling. Cell Adh. Migr. *8*, 339–348.

Wolff, J., Bhardwaj, V., Nothjunge, S., Richard, G., Renschler, G., Gilsbach, R., Manke, T., Backofen, R., Ramírez, F., and Grüning, B.A. (2018). Galaxy HiCExplorer: a web server for reproducible Hi-C data analysis, quality control and visualization. Nucleic Acids Research *46*, W11–W16. https://doi.org/10.1093/nar/gky504.

Wolff, J., Rabbani, L., Gilsbach, R., Richard, G., Manke, T., Backofen, R., and Grüning, B.A. (2020). Galaxy HiCExplorer 3: a web server for reproducible Hi-C, capture Hi-C and single-cell Hi-C data analysis, quality control and visualization. Nucleic Acids Research *48*, W177–W184. https://doi.org/10.1093/nar/gkaa220.

Xiang, W., Ke, Z., Zhang, Y., Cheng, G.H.-Y., Irwan, I.D., Sulochana, K.N., Potturi, P., Wang, Z., Yang, H., Wang, J., et al. (2011). Isthmin is a novel secreted angiogenesis inhibitor that inhibits tumour growth in mice. J. Cell. Mol. Med. *15*, 359–374.

Yang, T., Zhang, F., Yardımcı, G.G., Song, F., Hardison, R.C., Noble, W.S., Yue, F., and Li, Q. (2017). HiCRep: assessing the reproducibility of Hi-C data using a stratum-adjusted correlation coefficient. Genome Res. *27*, 1939–1949.

Zhang, C., Xu, Z., Yang, S., Sun, G., Jia, L., Zheng, Z., Gu, Q., Tao, W., Cheng, T., Li, C., et al. (2020). tagHi-C Reveals 3D Chromatin Architecture Dynamics during Mouse Hematopoiesis. Cell Rep. *32*, 108206.

Zhang, Y., Li, T., Preissl, S., Amaral, M.L., Grinstein, J.D., Farah, E.N., Destici, E., Qiu, Y., Hu, R., Lee, A.Y., et al. (2019). Transcriptionally active HERV-H retrotransposons demarcate topologically associating domains in human pluripotent stem cells. Nat. Genet. *51*, 1380–1388.
